# mHealth Series: mHealth project in Zhao County, rural China – Description of objectives, field site and methods

**DOI:** 10.7189/jogh.03.020401

**Published:** 2013-12

**Authors:** Michelle Helena van Velthoven, Ye Li, Wei Wang, Xiaozhen Du, Qiong Wu, Li Chen, Azeem Majeed, Igor Rudan, Yanfeng Zhang, Josip Car

**Affiliations:** 1 Global eHealth Unit, Department of Primary Care and Public Health, Imperial College London, London, UK; 2 Department of Integrated Early Childhood Development, Capital Institute of Paediatrics, Beijing, China; 3 Centre for Population Health Sciences and Global Health Academy, University of Edinburgh Medical School, Edinburgh, Scotland, UK

## Abstract

**Background:**

We set up a collaboration between researchers in China and the UK that aimed to explore the use of mHealth in China. This is the first paper in a series of papers on a large mHealth project part of this collaboration. This paper included the aims and objectives of the mHealth project, our field site, and the detailed methods of two studies.

**Field site:**

The field site for this mHealth project was Zhao County, which lies 280 km south of Beijing in Hebei Province, China.

**Methods:**

We described the methodology of two studies: (i) a mixed methods study exploring factors influencing sample size calculations for mHealth–based health surveys and (ii) a cross–over study determining validity of an mHealth text messaging data collection tool. The first study used mixed methods, both quantitative and qualitative, including: (i) two surveys with caregivers of young children, (ii) interviews with caregivers, village doctors and participants of the cross–over study, and (iii) researchers’ views. We combined data from caregivers, village doctors and researchers to provide an in–depth understanding of factors influencing sample size calculations for mHealth–based health surveys. The second study, a cross–over study, used a randomised cross–over study design to compare the traditional face–to–face survey method to the new text messaging survey method. We assessed data equivalence (intrarater agreement), the amount of information in responses, reasons for giving different responses, the response rate, characteristics of non–responders, and the error rate.

**Conclusions:**

This paper described the objectives, field site and methods of a large mHealth project part of a collaboration between researchers in China and the UK. The mixed methods study evaluating factors that influence sample size calculations could help future studies with estimating reliable sample sizes. The cross–over study comparing face–to–face and text message survey data collection could help future studies with developing their mHealth tools.

The use of mobile devices in health care, also known as mHealth or mobile health [1], has increasingly gained attention over the past years worldwide [2-8] and in China [9,10]. The different functions of mobile phones, such as calling, messaging, camera and apps, can be used for various health care–related purposes. A promising use of mHealth is data collection, both in high–income countries [11-19], and in low– and middle–income countries [20-30].

There are now almost as many mobile phones subscriptions (6.8 billion) as people in the world [31]. Mobile phones are a particularly interesting example of information and communication technology as they became the first to have more users in low– and middle–income countries than in high–income countries [32]. The growth in mobile phone subscriptions is led by China and India, which have over 30% of the world’s subscribers [33]. mHealth can be used in low– and middle–income countries to improve health systems and to reach the Millennium Development Goals [34-37].

However, though mHealth has the potential to improve health care, the current use of mHealth interventions in health care remains relatively low. Frequently mentioned barriers are methodological challenges and in result a lack of strong evidence for the use of mHealth [38-49].

In China, there were around 1.2 billion mobile phone subscriptions in 2013 [33]. Mobile phones are widely used both in urban and rural areas. While a relatively low proportion of households in rural areas have internet access or have a functioning landline telephone, nearly all households use at least one mobile phone [50].

We set up a collaboration between researchers in China and the UK, thereby combining our expertise in child health in China (Capital Institute of Pediatrics in Beijing), international child health and global burden of disease measurement (University of Edinburgh) and global mHealth (Imperial College London). The aim of this collaboration was to explore the use of mHealth in China. The Chinese researchers in this collaboration have a strong connection with the local health workers in Zhao County, Hebei Province in China, in which they have completed several child health studies during recent years [50-52]. Therefore, we selected Zhao County as a field site to conduct our mHealth research on child health data collection.

This is the first paper in a series of papers on a large mHealth project in Zhao County in rural China that is part of our collaboration. This paper included the aims and objectives of the mHealth data collection project, our field site, and the detailed methods of two studies that we conducted in Zhao County.

## AIMS AND OBJECTIVES

The aims and objectives of our mHealth project in Zhao County in China were the following.

### Aim 1: to advance mHealth data collection methodology

The first aim was to advance the mHealth methodology. We did this by two studies: (i) a mixed methods study exploring factors that influence the sample size of mHealth–based health surveys and (ii) a cross–over study determining the validity of an mHealth text messaging survey data collection tool. The methodology of these two studies was explained in detail in the methods section of this paper.

**Objective 1: explore factors influencing the sample size of mHealth–based health surveys.** Realistic sample size calculations are essential to conduct mHealth–based health surveys. There are several steps in the recruitment and follow–up of participants in mHealth studies where participants may be lost, from collecting mobile phone numbers to completing data collection. In text messaging data collection studies, an important issue affecting sample size calculation is the response rate of participants. Previous studies have reported variable response rates [11-13,15-17,19,23,27], but no studies have evaluated this issue and other problems in depth. The first study, a mixed methods study, explored factors influencing the sample size of mHealth–based health surveys. This will help future mHealth studies with estimating their sample sizes.

**Objective 2: determine validity of an mHealth text message data collection tool.** The validity of an mHealth data collection tool needs to be determined, because the mode of data collection can have great effects on data quality, especially when there are different modes of administration (interviewer–administered vs self–administered) [53]. While there are several studies that have compared mHealth text messaging data collection with other methods of data collection [11,14-16,19,23], most of these studies have only made within–group comparisons, used small samples, or only assessed properties of the used scale. The second study, a cross–over study, compared text messaging vs face–to–face interviews to determine validity of an mHealth text messaging survey data collection tool. This will help future mHealth studies with developing their mHealth tools.

### Aim 2: explore promising areas for mHealth data collection implementation

The second aim was to show how the advancements in mHealth methodology from the first aim could be used for three mHealth implementation areas: (i) to replace cross–sectional health surveys, (ii) to monitor program implementation, and (iii) to measure burden of disease in a community. The first and third promising areas of mHealth implementation were shown in this mHealth series [54] and the second implementation area will be presented elsewhere (unpublished).

**Objective 1: Explore the use of mHealth to replace cross–sectional health surveys.** In the first paper, we explain how mHealth text messaging surveys could replace cross–sectional surveys [54]. Large cross–sectional health surveys are required to provide valid estimates of health [55-58] and to measure coverage of health interventions [59]. However, conducting large scale interviewer–administered surveys are costly, time–consuming and can be difficult to perform. Pen–and–paper data collection are often the standard method in low– and middle income countries [60]. Using text messaging could be a more effective way for large–scale surveys, because it may decrease the number of field visits, include hard–to–reach populations, increase the survey sample size, eliminate interviewer bias and reduce recall bias.

**Objective 2: explore the use of mHealth to monitor program implementation.** In the second paper, we will show how mHealth could be used to monitor program implementation (unpublished). Planning and management are essential for health programs to achieve high coverage of key interventions and monitoring is crucial for process evaluation of intervention programs. However, often program monitoring data collection is difficult to perform, expensive and provides out–of–date and inaccurate results. mHealth data collection could facilitate monitoring, reduce costs and provide real–time data that could inform program management and planning [61].

**Objective 3: explore the use of mHealth to measure burden of disease in a community.** In the third paper, we explore how mHealth could be used to measure the burden of disease in a community (our unpublished results). There are very limited data available on the burden of childhood diseases and care–seeking for those diseases in developing countries [62-66]. Data are needed as appropriate health care strategies require a clear understanding of the burden of diseases [67]. mHealth could be a promising tool to measure the burden of disease, because the ubiquity of mobile phones allows mHealth tools to be easily scaled–up and used in different settings.

## GENERAL INFORMATION ABOUT ZHAO COUNTY

**Figure 1:**
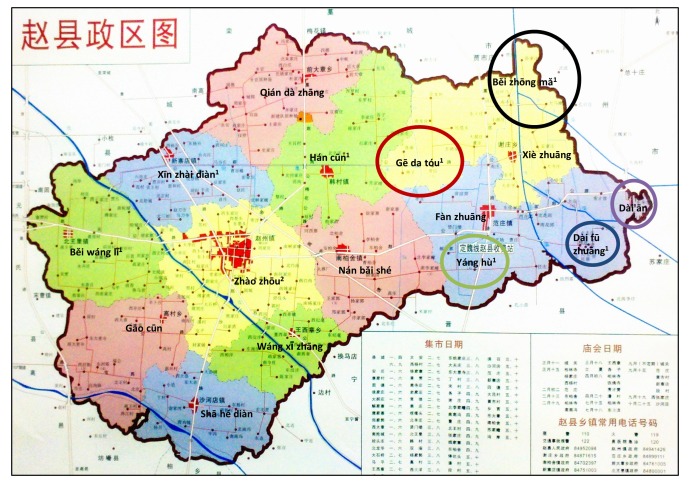
Map of Zhào County赵县. The map shows 11 coloured areas. For the nine townships on the left side of the map, these nine areas correspond with the nine townships: Běi wáng lǐ 北王里 (green left upper area), Gāo cūn 高村 (red left middle area), Shā hé dian 沙河店 (blue left lower area), Xīn zhai diàn 新寨店 (blue middle upper area), Zhao zhōu 赵州 (yellow central area), Wáng xī zhāng 王西章 (green middle lower area), Qián da zhāng 前大章 (red left upper area), Hán cūn 韩村 (green right upper area), Nán bǎi shé 南柏舍 (red right lower area). However, the two areas on the right side of the map correspond with seven townships: the two township areas (Xiè zhuāng 谢庄 (yellow right upper area) and Fan zhuāng 范庄 (blue right lower area)) and five townships that are marked with circles. The five circles correspond with the areas of the five townships and the names of the townships are written in the middle of the circles. Superscripts above the townships indicate when they are covered in survey 1 or in the remaining study. Townships for survey 1: Hán cūn 韩村 (green right upper area), Yáng hů 杨户 (around the green circle), Běi zhōng mǎ 北中马 (around the black circle), Běi wáng lǐ 北王里 (green left upper area), Xīn zhai dian 新寨店 (blue middle upper area), Gē da tóu 圪瘩头 (around the red circle), Dai fū zhuāng 大夫庄 (around the blue circle). Township for remaining study: Zhào zhōu 赵州 (yellow central area). Figure is the courtesy of Shuyi Zhang, personal collection.

Our field site was Zhao County in Hebei Province, China (**Figure 1**). This county has served as a field site for researchers from the Capital Institute of Pediatrics in Beijing since 2010 because of the following reasons: (i) previous survey data indicated low quality of care for children and high levels of inappropriate feeding practices; (ii) few maternal and child projects had been implemented in the county over the past 20 years; (iii) the socioeconomic development of Zhao County is similar to Hebei Province, which is similar to the national average; and (iv) the Zhao County Health Bureau and Zhao County Maternal and Child Health Hospital showed strong willingness to support quality improvement and good cooperation in research projects.

In China, the administrative levels are national, provincial, prefectural city, county, township and village. Hebei province is located in the northern part of the North China Plain with an area of 190 000 km^2^ (for comparison, the size of the UK is 250 000 km^2^), bordering the capital city Beijing. Hebei Province has a total population of 70.3 million, of which the urban population accounts for 43.7% and the rural population accounts for 56.3%. Shijiazhuang City is the Provincial capital of Hebei Province and administers Zhao County, which is one of the 114 counties in Hebei Province. Zhao County covers an area of 675 km^2^ and is located in the middle–south part of North China Plain, 40 km south of Shijiazhuang City [68]. Zhao County has 16 townships and 281 villages (ranging between 7 and 46 villages per township) [69]. The total population in Zhao County was 571 000, with 518 000 people (90.7%) living in rural areas in 2010. The female illiteracy rate was 3.76% in 2010 and the main ethnic group is Han (99.9%) in Zhao County (data from 2010 provided by the Zhao County Statistics Bureau, unpublished). The annual per capita net income of rural residents in Zhao County was ¥ 6464 (about £ 615, € 739, US$ 953), which is higher than the average for residents of Hebei province which was ¥ 5958 (about Ł 567, € 681, US$ 879) in 2010 (close to the national average of ¥ 5919) [70,71].

Zhao County is known for its agriculture, including Xuehua “snowflake” pears, wheat and corn. There are a number of famous historical sites in the county: Zhŕo zhōu “Arch” (Anji) Bridge赵州桥, Tuóluóníjīng Tower 陀罗尼经塔, and Bailin (Cypress Grove) Temple 柏林禅寺 (Online Supplementary Document(Online Supplementary Document), Field site photographs) [69].

### Health care structure in Zhao County

In China, there is usually a general hospital and a maternal and child health hospital at county level, one hospital in each township and one clinic in each village. All these health care facilities serve as primary health facilities where people can go to without referral [72].

Zhao County has four hospitals at county level including a public general hospital, a public maternal and child health hospital, a public traditional Chinese medicine hospital and a private general hospital. The county has 16 townships with each a public township hospital and 281 villages with each a village clinic. The government set that the basic public health services for maternal and child health care should mainly be provided at township level in this county. However, in practice women often seek this care at county or higher level hospitals [52]. Village clinics are privately–owned by village doctors who receive small subsidies from the government for providing public health services. Village doctors provide primary health care at village level and are trained and supervised by staff at township and county level [73,74]. Education and training of village doctors varies, but usually they have at least primary school or junior high school and short basic medical training. Village doctors live in the communities they serve and have a good relationship with villagers.

### Specific townships in Zhao County for different parts of the study

Zhao County has the following 16 townships: Hancun, Yanghu, Beizhongma, Beiwangli, Xinzhaidian, Gedatou, Daifuzhuang, Zhaozhou, Fanzhuang, Nanbaishe, Daian, Qiandazhang, Gaocun, Xiezhuang, Wangxizhang and Shahedian. The different parts of the study took part in eight of these townships: one survey that was part of the mixed methods study, survey 1, took place in seven townships and all the remaining parts of the mixed methods and cross–over study took place in one other township.

Survey 1 was undertaken in January 2013 with caregivers in the following seven townships: Hancun, Yanghu, Beizhongma, Beiwangli, Xinzhaidian, Gedatou, and Dajfuzhuang.

Survey 1 was part of a randomised controlled trial aiming to assess the effectiveness of infant feeding information sent via QQ (Tencent QQ), an instant messaging programme, in reducing anaemia prevalence (registered at China Ethics Committee for Registering Clinical Trials; registration number ChiECRCT–2012033). These seven townships were chosen for the QQ randomised controlled trial, because the other nine townships were not suitable. In those nine townships, other studies took place that could have introduced bias in the trial: in seven townships (Zhaozhou, Fanzhuang, Nanbaishe, Daian, Qiandazhang, Gaocun, and Xiezhuang) a study evaluating integrated management of childhood illnesses and in two townships (Wangxizhang and Shahedian) an mHealth study that aimed to evaluate the use of text messaging to monitor anaemia medication. In the seven townships included in survey 1, there were 107 villages, with an estimated total population of 206 600, under–five population of 12 700, and 3600 children aged 6–23 months [68].

All the remaining study parts for the mixed methods study and the cross–over study took place in Zhaozhou Township from January to March 2013. The nine townships that were excluded for survey 1 were eligible for the remaining study parts, because the two previously described studies finished in 2012. Of those nine townships, Zhaozhou Township was chosen, because this is the largest township with 46 villages and an estimated under–five population of 4170, and it has both an urban and rural population [68].

## METHODS

In this section we described the methodology of the two studies by which we aimed to advance mHealth methodology: the first study aimed to explore factors influencing the sample size of mHealth–based health surveys and the second study aimed to determine the validity of an mHealth text messaging data collection tool.

### Study 1: Factors influencing sample size calculations for mHealth–based health surveys, a mixed methods study

**Figure 2:**
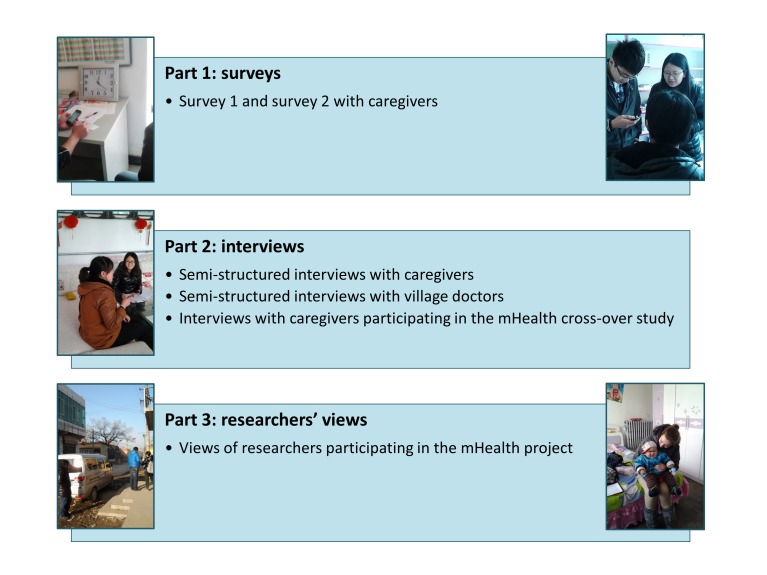
Overview of mixed methods study. Photographs are the courtesy of Michelle Helena van Velthoven and Wei Wang, personal collections.

**Overview of methods.** The aim of the first study was to explore factors that influence sample size calculations for mHealth–based health surveys and used mixed methods, both quantitative and qualitative. The methods of this study were described in three parts (**Figure 2**).

Part 1 described two surveys aiming to explore general characteristics of caregivers in Zhao County and their use of mobile phones in general and for health care; survey 1 was done with participants of the QQ randomised controlled trial (briefly described in the field site section) and survey 2 with participants of the cross–over study (described in the second part of this methods section). Part 2 described semi–structured interviews with caregivers about their general use of mobile phones and use for health care, semi–structured interviews with village doctors about recruitment of caregivers for the cross–over study and interviews with participants of the cross–over study about mHealth survey data collection methods. Part 3 described researchers’ views on mHealth survey data collection in China.

By combining quantitative and qualitative data from caregivers, village doctors and researchers, we aimed to provide an in–depth understanding of factors influencing sample size calculations in different stages of mHealth–based health surveys. The results of this study will be presented elsewhere, and in the sections below we described in detail the methodology. We structured the study parts according to the methodology used and started by describing the methods for the surveys in the section part 1: surveys [75], then the methods for the three different interviews in the section part 2: interviews [76], and finally how we combined these with views from researchers’ in the section part 3: researchers’ views.

**Part 1: Surveys.** The first part of the mixed methods study described two surveys: (i) a survey with participants of the QQ randomised controlled trial and (ii) a survey with participants of the cross–over study. The surveys aimed to explore general characteristics of caregivers in Zhao County and their use of mobile phones in general and for health care.

***1. Sample:*** The first survey was part of a baseline survey for the QQ randomised controlled trial (see field site section). We calculated that a sample of 816 had sufficient power for the main outcome of this trial, which was anaemia prevalence. We calculated that a sample size of 408 children aged 6–23 months in the intervention and 408 in the control group (816 children in total) was sufficient to show a between–group difference for all key indicators, with 80% power and a 5% significance level. We assumed an anaemia prevalence of 61.4% and we aimed to detect a difference in the decrease in prevalence of 10% between the intervention and control group. Based on the national trends in anaemia prevalence (anaemia prevalence in children declines when they grow up), we expected the anaemia prevalence to decrease from 61.4% to 47.4% in the control group and from 61.4 to 37.4% in the intervention group.

Participants in the trial had to use the QQ program and we expected that 50% of caregivers used QQ. Therefore, we doubled the 816 participants that had to be included in the survey and we aimed for a sample size of 1632 participants. While the sample size calculation for the mHealth survey was not specifically calculated for mobile phone use indicators, the trial’s sample size was more than enough to provide information on caregivers’ mobile phone use. The seven townships had an estimated 3600 children aged between six months and two years on the name list (see field site section). We expected that an estimated 2400 caregivers (70%) on the name list were willing to participate and complete the survey, which was sufficient for the trial’s sample size, even if fewer caregivers were willing to participate.

For the second survey with caregivers participating in the mHealth cross–over study, we aimed for a sample size of 1095 participants. This was described in the sample size section for the cross–over study in the second part of this methods section, because it required an explanation of the cross–over study design.

***2. Participants:*** Survey participants were eligible for both surveys when they were a caregiver of a child aged between six months and two years. Caregivers were excluded if they had a child of a different age, if they were not willing to participate, or if they were unable to read or understand the informed consent materials. We gave caregivers a towel (worth ¥ 5, about £ 0.52, € 0.62, US$ 0.82) for their time to complete the face–to–face survey.

***3. Recruitment:*** A doctor from Zhao County Maternal and Child Health Hospital (XS) was our local information person and helped us during the fieldwork. He had good connections with local people at different levels of the health care system and was experienced with health care research.

XS obtained a list with names of children in each village in the seven townships from the township hospital doctors. We decided to ask the village doctors to help us with recruiting caregivers in the village clinics, because many caregivers were familiar with their village doctor. Before the study started, village doctors were contacted on three occasions. First, the township hospital doctors contacted all doctors in the villages about the study and asked them to participate. Second, two days before the study started the township hospital doctors asked the village doctors when it was convenient to visit their villages, to check the township hospital name list, and to inform caregivers when they should come to the village clinic. The township hospital doctors informed XS during these steps. Third, half an hour before the interviewers arrived in the village, a township hospital doctor or XS asked the village doctor to start recruitment of caregivers.

We used a number of recruitment strategies to encourage caregivers to come to village clinics and these included the following: using loudspeakers in the villages, making phone calls to caregivers, visiting caregivers in their homes, asking caregivers to ask their neighbours, asking people on the street, going to places that caregivers visit, such as a market, and to gatherings of people such as a wedding. When possible, we used loudspeakers that were available in the villages and we asked the village doctor make the following announcement: “We are from the Capital Institute of Pediatrics and Zhao County Maternal and Child Health Hospital, if you are a parent of a child aged over six months and younger than two years, you can take the child to the village clinic, it is best if the mother comes, we will do a survey and then get a free test for anaemia, you can wait to get the result”. For some of the villages, the hospital doctor was able to obtain a list of phone numbers from the local immunization service centre that we used to call caregivers. For other villages, the hospital doctor was unable to obtain this list as these villages belonged to an immunization service centre that was not willing to provide the phone numbers.

Village doctors were the main contacts for the recruitment of caregivers, because based on previous experiences we knew that caregivers were more willing to participate if their village doctor asked them. We expected that village doctors were familiar with all births in their village, because they reported newborns to the township hospital each month. Therefore, we expected them to be able to recruit a significant number of caregivers from their own records. However, we anticipated that not all village doctors were willing to help us, but that they did not tell this to the township hospital doctor, XS or to us in advance. We expected that less willing or busy village doctors made fewer efforts to find caregivers of children on the name list. Therefore, we gave village doctors a small financial incentive for their efforts to increase their willingness to participate. For survey 1, we gave village doctors ¥ 50 per village (about £ 5.3, € 6.2, US$ 8.2). For survey 2, we used a different approach to incentivize village doctors. We told village doctors that they received ¥ 50 for recruiting 55 caregivers, or fewer caregivers when their village had a smaller number of children under five. When village doctors recruited more caregivers, the amount they received increased with ¥ 10 (¥ 60 for 55–65 caregivers, ¥ 70 for 66–75 and so on).

When the interviewers arrived in the village clinics, they informed eligible caregivers about the study procedures, asked them to read the information sheet and gave them the opportunity to ask questions. The interviewers informed caregivers that the study results were not used to assess the health of their child, and that if they have any concerns about the health of their child, they should contact a health worker. Also, interviewers told caregivers that they could decide to withdraw from the study at any moment and that this did not influence the health care they received. Interviewers asked caregivers who were willing to participate if they understood what participation in the study included and to sign the informed consent form.

***4. Interviewers:*** The supervisors for survey 1 (WW, YL, and BL) and survey 2 (WW and XD) were all experienced in supervising surveys in Zhao County. For both survey 1 and 2, the interviewers were medical students from local universities. The supervisors trained the students thoroughly on survey techniques the day before the study started. The training included: introduction to the survey aims, obtaining informed consent, use of a smartphone for recording the answers, a detailed explanation of every question, and practising interviewing. The students practised with a partner in pairs through role play and discussed their experiences with the whole group. The supervisors carefully monitored the students, gave constructive comments, and validated the students asking of questions. We tested the students at the end of the training; two supervisors played as actors, one took the role of an interviewer and another took the role of a caregiver. The supervisors were experienced in role–play and we used this as the “gold standard”. The supervisors asked all students to record what the “caregiver” answered with a smartphone provided for the study. The supervisors compared the recorded data of all the students with the “gold standard”.

For survey 1 with caregivers participating in the randomised controlled trial, there were a total of 347 questions in our survey instrument and the overall agreement for all questions for all the students was more than 97%.

The results of the training for second survey with caregivers participating in the mHealth cross–over study was described in the interviewers section in the second part of this methods section.

***5. Data collection:*** Interviewers used a smartphone to record answers of caregivers to the survey questions in the village clinics with reasonable privacy. We validated smartphone data collection for the Chinese maternal, newborn and child health (MNCH) survey in Zhao County; compared to pen–and–paper data collection, smartphone data collection can avoid data recording and entry errors, has a similar interrater reliability and takes the same amount of time per interview [50]. The first survey was carried out by three teams of interviewers; there were two large groups of ten interviewers and one smaller group of seven interviewers (27 interviewers and 3 supervisors in total). Data collection for the second survey with caregivers participating in the mHealth cross–over study was described in the data collection section in the second part of this methods section.

***6. Questionnaires:*** Survey 1 consisted of an identification, mobile phone, QQ and household module (Online Supplementary Document(Online Supplementary Document), Survey 1 on demographics and mobile phone use – English version and Survey on demographics and mobile phone use – Chinese version). We selected demographic questions from the identification and household modules of the World Health Organization’s (WHO) MNCH Survey. We adapted questions from these modules to the local context in Zhao County and used them in previous research [50]. The questionnaire for the QQ randomised controlled trial included additional questions for the identification, QQ and household modules and four other modules relevant to the trial.

For the second part of the survey, a researcher fluent in English (MV) developed the mobile phone related questions in discussion with the Chinese researchers (YZ, YL, and WW) and an mHealth expert (JC). Then two Chinese researchers (WW and YL) translated the mobile phone related questions independently from English into Chinese (Mandarin). They compared the translations and disagreements were discussed with a third Chinese researcher (YZ). Then, a bilingual (Chinese and English) translator (EC) checked whether the meaning of the translated questions was comparable between Chinese and English. We tested the mobile phone questions with caregivers Zhaozhou Township and made minor changes in the questions to ensure that the questions were understandable and appropriate.

Survey 2 (see Online Supplementary Document(Online Supplementary Document), for the English and Chinese versions of Survey 2 on demographics and mobile phone use) was a simplified and slightly altered version of survey 1. We selected the most important survey questions to reduce the workload for interviewers and participants. The questionnaire for the cross–over study included two other modules relevant to the study.

***7. Data management:*** When interviewers completed the face–to–face questionnaire, the data was wirelessly and securely uploaded into an Excel database via an internet server. The data was also saved on the memory card of the smartphone as an encrypted file. The data could only be decrypted with special software. The supervisors collected the smartphones at the end of each field work day. They returned the smartphones cleared from the data that was entered during the previous day to the interviewers in the morning. Only the supervisors were able to enter databases, and no changes could be made to databases. Each participant was given an identification number and the databases with participant information linked to the identification numbers could only be accessed by the researchers. We anonymised data for analysis and reporting.

***8. Data analysis and outcomes:*** We used SPSS version 16.0 [77] for the statistical analysis of the quantitative data. We calculated proportions, medians (Q2), 25 (Q1) and 75 (Q3) percentiles for the demographic, and mobile phone use indicators. We did not impute missing data.

**Part 2: Interviews.** The second part of the mixed methods study described three types of interviews: (i) semi–structured interviews with caregivers about their general use of mobile phones and use for health care, (ii) semi–structured interviews with village doctors about recruitment of caregivers for the cross–over study, and (iii) interviews with participants of the cross–over study about mHealth survey methods.

***1. Methodological orientation and theory:*** For all the interviews, we used thematic analysis [78], which is a method for identifying and analysing themes within data. We aimed to provide a rich thematic description of the entire data set that reflected the important themes. As an alternative to thematic analysis, we considered using grounded theory, which is a useful method for investigating an under–researched area [79]. We felt that grounded theory was the most appropriate analysis approach for our research, because there was no literature on the research topic and we were interested in knowing caregiver’s perspectives and experiences. However, due to fieldwork and time constraints, a thorough grounded theory analysis was not feasible. Therefore, we chose a thematic analysis and used principles of grounded theory where possible. We used an inductive or bottom coding method by which the themes identified had to be strongly linked to the data themselves, which was somewhat similar to a grounded theory approach. We used a realist approach which assumes that motivations, experience and meaning are directly related to language. This allowed reporting meaning and experiences in a straightforward way.

***2. Sample size:*** In *semi–structured interviews with caregivers,* we aimed for the sample size to be large enough to cover the diverse views of caregivers and to reach saturation of themes. Saturation is reached when no new themes emerge from the interviews. Between 12 and 60 interviews is generally enough [80] and saturation is commonly reached within 20 interviews [81]. Initially we planned to interview 15 to 20 caregivers. We analysed data from the first round of 15 to 20 interviews and used the saturation principle to determine the final number of participants. Additional interviews were held if saturation was not reached within the first 15 to 20 interviews.

We selected villages in semi–urban and rural Zhaozhou Township for recruitment of participants. We aimed to select the sample based on characteristics that we considered to be relevant: type of caregiver, age, number of children, urban or rural residence, education and type of mobile phone (low–end mobile phone or smartphone). Also, we aimed to look for dissonant cases to gain insights from people who were unusual in some way. We used snowballing and asked participants if they knew any other caregivers who were able to participate.

In *semi–structured interviews with village doctors*, we conducted semi–structured interviews with village doctors who participated in the cross–over study. We used a similar approach for the semi–structured interviews with village doctors as the approach that we used for the interviews with caregivers. We planned to interview between 10 and 20 village doctors (out of the 46 village doctors in total). We aimed to obtain a variety of views from village doctors and we selected both female and male village doctors from different villages. We recruited them based on their willingness to participate and the available time that interviewers had when they visited villages during the cross–over study.

In *interviews with caregivers participating in the mHealth cross–over study*, we undertook interviews with participants of the cross–over study within one week after completion of the cross–over study. We interviewed three groups of participants of the cross–over study: (i) participants who completed the text message survey; (ii) participants who responded to at least one text message question; and (iii) participants who did not respond to any text message questions. We aimed to interview 50 participants in each group. We anticipated that 50% of the participants who we asked were willing to participate. Therefore, we randomly selected a sample of 100 participants for each group. We considered that participants who completed the text messaging part of the study may have been more likely to participate in the interviews. However, we also considered that participants who did not complete the text messaging part could have been more willing to answer a phone call than respond via text messaging. Therefore, we did not adjust the numbers and we used a similar number of participants for each group.

***3. Participants and recruitment:*** In *semi–structured interviews with caregivers*, all interview participants were eligible if they were a caregiver of a child younger than five years of age and used a mobile phone. Two days before the study started, the Maternal and Child Health Hospital doctor asked the village doctors when it was convenient to visit their villages. When we arrived in the village clinic, we asked village doctors to find caregivers that were willing to participate. We used an approach similar to the surveys for obtaining informed consent and we explained caregivers the aims of the semi–structured interviews.

In *semi–structured interviews with village doctors*, we included village doctors that participated in the cross–over study. We excluded village doctors who were not willing to participate. The supervisors asked village doctors to participate when they visited villages during the cross–over study.

In *interviews with caregivers participating in the mHealth cross–over study*, we recruited caregivers for the cross–over study and this was described in detail in the sections “participants” and “recruitment” in the second part of this methods section.

***4. Interviewers and data collection:*** In *semi–structured interviews caregivers*, a native female Chinese researcher (YL) did the semi–structured interviews with caregivers in Chinese. A female researcher fluent in English (MV) with experience in qualitative research was present during the interview to help YL and to record any non–verbal communication and observations. MV trained YL on qualitative research, which included an explanation of qualitative methods and interview techniques (eg, how to ask open–ended questions), and practice with team members and caregivers in Zhao County. MV made several visits to China and visited Zhao County so that she had an understanding of the Chinese research context.

YL aimed to summarize her understanding of what the caregiver said two times during the interview to verify her understanding of the caregiver’s views. YL translated parts of the interview content several times during the interview to allow MV to ask additional questions. The use of an interpreter was not feasible as there was no person in the research team who was a native speaker both in Chinese and English. However, the use of an interpreter may have been less desirable as this could have influenced the flow of the interview. The researchers reflected after each interview and at the end of each fieldwork day, and recorded ideas. The interviews were carried out at a neutral and private location that was comfortable for the participants, often the participants’ home, or if that was not possible, a quiet room in the village clinic. We asked participants if we could interview them alone and if they could ask their family members and other people not to disturb us during the interview. When the participant gave permission, the interview was recorded with a digital recorder, notes were taken to record non–verbal communication and photographs were taken of the caregiver and child (with face not identifiable and with their verbal and written permission). The interviews took between 15 and 60 minutes. We did not carry out repeat interviews with the same participants to go further into depth, because this was not feasible.

In *semi–structured interviews with village doctors*, the two female supervisors in the cross–over study (WW and XD) conducted the semi–structured interviews with village doctors’ interviews in Chinese. MV trained WW and XD on qualitative research, but did not take part in the interviews. The interviews were carried out at a neutral and private location that was comfortable for the village doctor, usually a quiet room in the village clinic. When the village doctor gave permission, the interview was recorded, and notes were taken to record non–verbal communication. The supervisors and MV reflected after the interviews and noted ideas.

In *interviews with caregivers participating in the mHealth cross–over study*, we used telephone interviews and face–to–face interviews for the interviews with caregivers participating in the cross–over study. The two supervisors of the cross–over study (WW and XD) interviewed the second group of participants (those who responded to at least one text message question) face–to–face, because they were able to do this during the fieldwork. In this group, the supervisors also asked participants if they could check the mobile phone of the participant to confirm whether they received text messages (when the same person participated face–to–face and via text messaging and brought their mobile phone). We conducted telephone interviews with participants in the first group (those who completed the text message survey) and third group (those who did not respond to any text message questions), because we could not interview these participants face–to–face. Four team members (WW, XD, YL, and QW) conducted the telephone interviews. They called participants at a time convenient for participants and when the phone call was unanswered, they called participants back up to three times. The interviewers used a pen–and–paper questionnaire to record the interview.

***5. Questionnaires:*** All the interview guides were developed in a similar way as the mobile phone questions in the survey. In addition, we asked a Chinese sociologist (YQ) for advice for the semi–structured interviews with caregivers. We used probing questions (asking open–ended questions; questions starting with how, why, what etc.) to follow–up on the questions in the guide, because an in–depth understanding of topics usually comes from probing [82].

In *semi–structured interviews with caregivers*, we did not define specific research questions at the start of the interviews, but we had initial broad research questions. The initial aims of the interviews were to better understand: (i) how caregivers use their mobile phones and (ii) what caregivers’ experiences were when using a mobile phone for seeking health care (Online Supplementary Document(Online Supplementary Document), Topic guides for semi–structured interviews with caregivers, Topic guide 1). Halfway the interviews we felt that we reached saturation on these aims and we refined the questions into the following: (i) which factors influence whether caregivers respond to text messages and (ii) what caregivers’ experiences were with seeking information for their child’s health via their mobile phone (Online Supplementary Document(Online Supplementary Document), Topic guides for semi–structured interviews with caregivers, Topic guide 2).

In *semi–structured interviews with village doctors,* the aim of the interviews was to better understand willingness of village doctors to recruit caregivers. The interview guide (Online Supplementary Document(Online Supplementary Document), Topic guide for semi–structured interviews with village doctors) included questions around how village doctors found it to recruit caregivers for the mHealth cross–over study. We had the following research questions: (i) how many caregivers were village doctors able to find, (ii) what motivated them to find caregivers, and (iii) what they thought that influenced caregivers’ willingness to come to the clinic.

In *interviews with caregivers participating in the mHealth cross–over study*, the aim of the interviews was to explore factors that influence participation of caregivers in mHealth studies. We made three specific questionnaires for the three different groups of participants (Online Supplementary Document(Online Supplementary Document), Questionnaires for interviews with caregivers participating in the mHealth cross–over study). The questionnaire included open–ended questions about how caregivers found it to reply to text messages and which method of answering questions they preferred. Also, the questionnaire included closed–ended questions for which we asked participants to respond with a number, such as how many text messages caregivers were willing to answer at most on a day.

***6. Data management:*** In *semi–structured interviews with caregivers and village doctors*, local students transcribed the recorded data verbatim in Word 2007. These transcriptions were checked by students and rechecked by the same person who conducted the interviews by listening to the tapes. We kept the recorded data and transcripts on a secure computer and anonymised all the data. We sent transcripts to participants and asked them to return them, because the interviewer summarized her understanding of what the participants said during the interviews.

In *interviews with caregivers participating in the mHealth cross–over study*, two students transcribed the pen–and–paper form in an Excel database and a Chinese team member (YL) compared the data and completed the final database. Any discrepancies were addressed by discussing this with a team member (XD).

**7. *Data analysis and outcomes:*** In *semi–structured interviews with caregivers*, we used computer–aided qualitative data analysis software MAXQDA 11 to analyse the qualitative data (VERBI Software – Consult – Sozialforschung GmbH; Berlin, Germany). The interviews with caregivers were transcribed in Chinese and translated into English, because the team member fluent in English (MV) was involved in the fieldwork and analysis. The Chinese interviewer (YL) conducted the analysis in Chinese and MV did the analysis in English. To obtain the transcripts in English, YL translated the transcripts from Chinese into English. A second Chinese team member (WW) checked the translations and MV checked the English language and discussed the meaning of the transcripts with YL. Then YL analyzed the data in Chinese and MV analysed the data in the English.

We conducted our thematic analysis in six steps. First, two researchers read through the interviews several times in an active way (searching for meaning) to obtain an overview of the interview data. Second, initial codes were given to findings (units of texts). We coded for as many possible findings as possible (including context), gave full and equal attention to each data item and kept data which was different from the main story. The two researchers compared their coding after each interview. Third, we searched for themes (group of codes that are similar and capture something important about the data) and sorted codes into potential themes. New themes were added until no new themes emerged from the findings. We only looked for semantic (explicit) level themes; we did not look beyond what a participant said. The two researchers carried out this process independently and discussed their findings. Fourthly, we reviewed the themes on two levels: of the coded data extracts and in relation to the data set. We reviewed coded data extracts and constantly compared them in relation to the data set to consider validity of the themes. We continued reviewing this until we had a good idea of what the themes were, how they fitted and the overall story of the data. Fifthly, we defined and named themes. Sixthly, we related the different themes to each other to develop an explanation in relation to the research question [83], we choose vivid quotes which captured the essence of key points and we wrote the “story” (analysis).

YL translated the Chinese themes into English and MV compared these with the English themes. The bilingual translator (EC) translated the final English version of the themes back into Chinese and compared them with the original Chinese themes [84]. Throughout the analysis, we kept memos to capture our thought processes. We compared our fieldwork memos and observations with the analysed data. We discussed the analysis within the research team and with a qualitative researcher and Chinese sociologist (YQ) to verify the understanding of the interpretation. We did not ask participants for feedback, because this was not feasible in this research setting. We presented a narrative of the main findings.

In *semi–structured interviews with village doctors*, we used an approach similar to the one that we used for the semi–structured interviews with caregivers for the semi–structured interviews with village doctors. However, there was one important difference; these interviews were done by two Chinese researchers (YL and WW) and also analyzed by two Chinese researchers (YL and DX) in Chinese. After analyzing the data in Chinese, YL and DX independently translated the themes into English. They compared the two Chinese–English translations and discrepancies were solved by consulting a third Chinese team member (WW). EC translated the final English translation back into Chinese and compared with the original Chinese concepts [84].

In *interviews with caregivers participating in the mHealth cross–over study*, we used Excel version 2010 for the analysis of the interviews with participants of the mHealth cross–over study. We calculated proportions and medians (Q2) with 25 (Q1) and 75 (Q3) percentiles for the questions where the interviewers categorized the response of the caregiver and for questions where a number was asked.

For the open–ended questions, we did a simplified version of a thematic analysis, because telephone interviewing allowed less for in–depth probing. Two Chinese researchers (YL and WW) independently read through the data several times, identified the main themes in the data and summarized the results in Chinese. The approach for translation of the results was similar to the approach for translation of the results of semi–structured interviews with village doctors. We presented a narrative of the main findings.

**Part 3: Researchers’ views.** Researchers kept a log book during the fieldwork to capture ideas about factors influencing sample size calculation for mHealth–based health surveys. A team member (MV) wrote a narrative of these notes and all researchers involved in this project contributed their views. The views were compared with the analysis of the surveys and interviews and the findings were added to the narrative.

### Study 2: Comparison of text messaging vs face–to–face interviews for health surveys, a cross–over study

**Figure 3:**
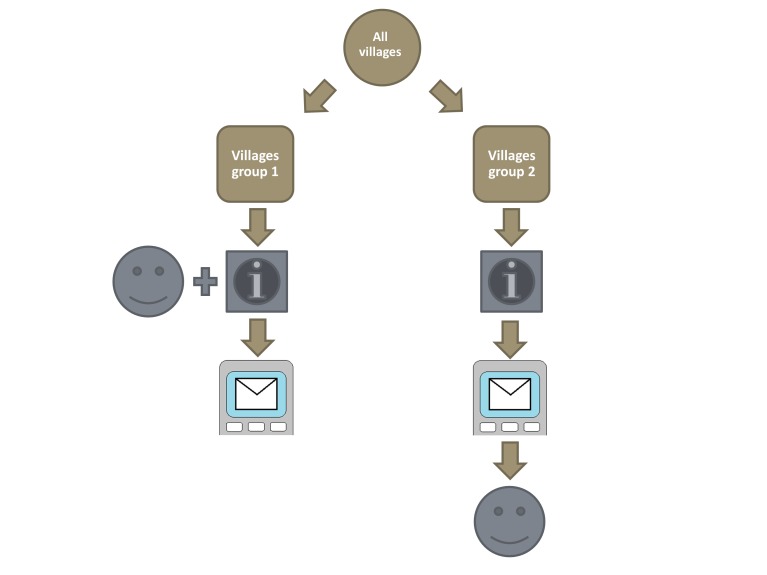
Design of randomised cross–over study. Letter clipart indicates informed consent and collection of demographic and mobile phone use information; face clipart indicates interviewer–administered face–to–face survey on childhood diarrhoea and pneumonia; mobile phone clipart indicates self–administered text messaging survey on childhood diarrhoea and pneumonia. Images were created in Microsoft PowerPoint 2010 or Paint.

**Overview of methods**. The aim of the second study was to determine the validity of an mHealth text messaging survey data collection tool. We used a randomised cross–over study design to compare the traditional face–to–face survey method to the new text messaging survey method (**Figure 3**). We randomised participants per village into two groups: group 1 first completed the face–to–face survey and then the text message survey, and for group 2 this order was reversed. Participants were caregivers taking care of a child younger than five years. We compared 18 questions on care–seeking for childhood diarrhoea and pneumonia signs and symptoms that we selected from the WHO MNCH household survey. The text messaging survey had two additional questions (19 questions in total): the first question asked about the agreement of the caregiver to participate and the second question asked about the relationship between the caregiver and the child. In addition to those two questions on agreement and relationship, participants had to answer a minimum of four text message questions about disease symptoms (diarrhoea, fever, cough, fast or difficult breathing). Depending on whether their children had these symptoms, we asked participants one or more of the 14 follow–up questions. We compared responses of caregivers between the face–to–face and text messaging methods and evaluated how similar responses were by data equivalence (intrarater agreement) and the amount of information for the open–ended questions 13 and 19 (places where care was sought), and participants’ reasons for differences. We analysed the overall response rate (proportion of completed interviews), item response rate (the proportion of responses for each question) differences between responders and non–responders, and the error rate of the text messaging method. We described the detailed methods of the cross–over study in the sections below and the results will be presented elsewhere (unpublished).

**Figure 4:**
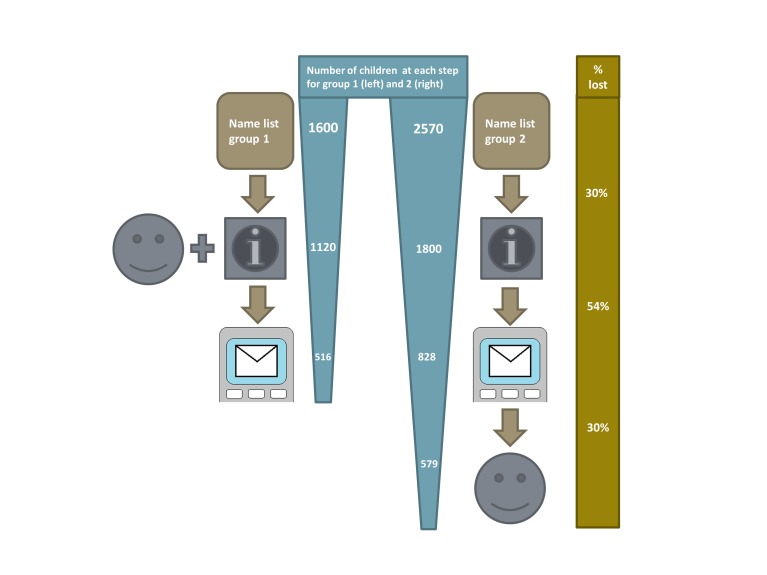
Rough estimation of the expected number of participants of cross–over study. Letter clipart indicates those expected to complete informed consent and collection of demographic and mobile phone use information; face clipart indicates those expected to complete interviewer–administered face–to–face survey on childhood diarrhoea and pneumonia; mobile phone clipart indicates those expected to complete at least one question from self–administered text messaging survey on childhood diarrhoea and pneumonia. Images were created in Microsoft PowerPoint 2010 or Paint.

***1. Sample size****:* We were unable to conduct a sample size calculation for the cross–over study, because we did not have accurate estimates from previous research that could inform a calculation. Therefore, we estimated a rough number of participants that we could recruit in the study setting (**Figure 4**). We aimed to include 1095 participants from Zhaozhou Township; 516 participants in group 1 and 579 participants in group 2. Zhaozhou Township had an estimated under–five population of 4170 children according to a name list provided by the township hospital. Based on previous experiences, we expected that 70% of caregivers approached participated, that 40% responded to at least one text message, and that 10% responded to a reminder text message (about 46% responded to either a text message or reminder). We expected to recruit 516 caregivers for group 1 based on these expected proportions; if we approached 1600 caregivers for group 1, 1120 participated face–to–face, 448 responded to at least one text message and 68 responded at least once after a reminder text message. For group 2, we expected that more participants dropped out, because a second visit to the clinic was required for the face–to–face interview. Therefore, we oversampled the number of caregivers that we planned to approach in group 2. We expected to recruit 828 caregivers for group 2; if we approached 2570 caregivers, 1800 agreed to participate, 720 responded to at least one text message, 108 responded at least once after a reminder text message, and 579 participants who responded to at least one text message also participated face–to–face.

***2. Randomisation:*** We used stratified randomisation, taking into account the size of the village, to divide villages into two groups. The aim of stratified randomisation was to avoid imbalances in baseline characteristics as the caregivers fell into obvious strata (the size of the village). Individual randomisation could have introduced bias, because participants from two groups living in one village could then have shown each other the text messages. This could have influenced the responses they gave and thereby have biased the results. Also, we could not randomise participants on an individual level for fieldwork organisational reasons; it was very difficult to double the number of visits to villages and at the same time keep the time interval between recruitment and the surveys the same (we visited caregivers in villages in group 1 once and caregivers in villages in group 2 twice and this was on set days to keep the time interval the same).

The villages had an estimated under–five population ranging between 20 and 335 children. As this population size per village was highly variable, we had to take the size of the village into consideration to prevent major imbalances in the groups. We did not have the option of randomising the villages according to a particular characteristic, because we did not have any information available. We used SAS version 9.2 (SAS Institute Inc, Marlow, UK) for the randomisation and we ranked villages based on their under–five population sample size into three strata of 15, 15 and 16 villages each. We chose a small number of strata, because we needed to ensure a sufficient number of individuals in each stratum. An independent statistician provided us with a list of random numbers to determine the strata that had 16 villages. The ranking meant that the size of the village was randomly used for allocation to one of the strata. For example, if stratum 1 had the smallest villages, stratum 2 had the medium sized villages, and stratum 3 the largest villages. Then we randomised the villages in each stratum into group 1 or group 2; we gave a random number to each village and assigned villages to the groups. We used block–randomisation with a ratio of 1:1.6 to allocate a larger proportion of participants to group 2 and to account for the expected higher drop–out (see sample size section).

***3. Recruitment:*** We used a similar strategy for recruitment as we described in the section “surveys” in the first part of this methods section. We made the following announcement: “We are from the Capital Institute of Pediatrics and Maternal and Child Health Hospital in Zhao County. We are doing a survey of children and we will ask you about your child's health in the past two weeks. If you are a parent of a child younger than five years, come to the village clinic around: *appropriate time*. You have to be able to receive and send text messages to participate. You do not need to bring the child, because we do not do physical examination. After the interview, you will get a towel to thank you for your time and effort to participate.”

***4. Participants:*** Caregivers were eligible if they took care of a child younger than five years of age, used a mobile phone and were able to send a text message. Caregivers were excluded if they were not willing to participate, if they were unable to read or understand the informed consent materials, if they did not have a mobile phone, or if they could not sent a text message. Based on our previous experiences, we knew that many grandparents were unable to text message and therefore we did not actively recruit grandparents. However, as many grandparents take care of the child(ren) of their son and some of them can text message, we also considered them for eligibility. We checked with care whether grandparents were able to text message. We asked them to send us a test message in which they had to write the name of their grandchild or spell the “immunization card” (five Chinese characters, spelled 预防接种证).

We gave caregivers a towel (worth ¥ 5, about £ 0.52, € 0.62, US$ 0.82) for their time to complete the face–to–face survey. We gave caregivers who participated in the text messaging survey ¥ 5 for completing the text message survey, and we paid back the costs of the text messages (sending a text message in China costs ¥ 0.1, about £ 0.10, € 0.12, US$ 0.16) by mobile phone credit payment within a week. We told caregivers that they received ¥ 0.1 per text message that they sent. However, for the payment we had to pay a minimum of ¥ 1 to each participant as the mobile payment could not be less than that. We also paid ¥ 1 to participants who responded “not willing” to the first question and we paid ¥ 5 to participants who almost completed the survey (the last question required a second text message response after a prompting question, but some participants responded only once to this last question; however, they may have felt that they completed the survey). The payment was made before the interviews aiming to explore reasons for not responding to text messages (see sections on “interviews with caregivers participating in the mHealth cross–over study”).

We informed caregivers in the first group that they were asked to participate in the text messaging survey the day after the face–to–face survey. We informed caregivers in the second group that they were asked to participate in the text messaging survey two days after signing the informed consent and that they had to visit the village clinic again in four days to complete the face–to–face survey.

***5. Interviewers:*** We performed face–to–face and text messaging surveys.

In the *face–to–face survey*, there were 14 interviewers for the face–to–face interviews: 10 undergraduate medical students from a local university, one postgraduate medical student, and two supervisors (WW and XD). After recruitment of participants in the first group, five students had to leave half way because of their studies and five new students were trained to interview participants in the second group. All interviewers were familiar with the dialect in Zhao County, which is slightly different from standard Chinese. The supervisors were experienced child health survey researchers who had done previous surveys in Zhao County.

During the training, we calculated inter–observer agreement, the proportion of agreement for each question between students and intra–observer agreement, the proportion of agreement for each student. There were a total of 50 questions and 100 variables (answer options) in our survey instrument. For the first round, the intra–observer agreement for all questions for eight students was more than 96%. Two students scored lower, for one it was 77% and for one 83%, because they misunderstood the principle of skipping some of the questions. Therefore, we explained the questions with wrong answers to the two students, and did the validation test again. Then the agreement increased to 98% for both students. The inter–observer agreement was 95% for the first round and 98% for the second round. For five students who replaced the five students that had to leave, the intra–observer reliability was more than 96% and inter–observer reliability was 98%. To further optimize the reliability, we discussed and explained all questions that posed problems and provided help to students who needed assistance. The supervisors checked whether each student was doing the survey correctly during fieldwork. The supervisor stood next to the interviewer and participant during the whole process on the first day for every interviewer.

In the *text messaging survey*, one researcher (YL, based in Beijing) sent the text messages and a second researcher (first QW, then by two trained students) checked the text messages. The first researcher trained the second researcher by giving an introduction to the study, explaining the algorithm and checking the text messages. To ensure that the second researcher understood the procedures, the initial checks that the second researcher did were rechecked by the first researcher. Any problems or inconsistencies were addressed appropriately.

We used a Chinese text message system (Shāng jī bǎo 商机宝) for sending text messages to participants and receiving text messages from them. We chose the best system based on experiences with three Chinese text message systems. We tested the chosen system during pilots and a previous study (unpublished). We checked the text messaging system for incoming response text messages (Online Supplementary Document(Online Supplementary Document), Description of sending text messages). We exported all the incoming text messages into an Excel file and prepared the appropriate follow–up text messages by following a protocol. This procedure could not be done automatically by the text message system. To prevent errors from occurring in this process, the second researcher checked the text messages that the first researcher prepared before they were sent. In case there was disagreement or confusion, a third researcher (MV) was consulted for advice.

***6. Questionnaires:*** The questionnaire in the *survey on demographics and mobile phone use*, was described in the questionnaires section for the first study in this methods section.

In the *face–to–face and text messaging questionnaire on care–seeking for childhood diarrhoea and pneumonia*, we selected 18 questions from the diarrhoea module and cough and fever module (used to assess pneumonia) from the WHO's MNCH Health survey. The child health survey experts from the Capital Institute of Pediatrics (YZ, LC, QW, YL and WW) translated the questions into Chinese, adapted them to the local context in Zhao County, tested the questions during pilot research and used them in large household surveys in 2010 and 2011 (unpublished data).

The text message survey included the same 18 selected questions from the diarrhoea and cough and fever modules that we used for the face–to–face survey. Of those 18 questions, 3 questions (10, 13, and 20) had follow–up questions (10a, 13a, and 20a). In addition, the text message survey had two additional questions: the first question asked about agreement to participate and the second question asked about the relationship between the caregiver and the child. In addition to the two questions on agreement and relationship, participants had to answer a minimum of four text message questions about disease symptoms (diarrhoea, fever, cough, fast or difficult breathing). Depending on whether their children had these symptoms, participants had to answer one or more of the 13 other questions.

We adapted the questions to make them fit into the text messages in an interdisciplinary team of child health (IR) and child health survey experts (YZ, LC, QW, YL and WW). During the development of the text message survey, we aimed that the text message questions had the same meaning as the face–to–face questions (Online Supplementary Document(Online Supplementary Document), Detailed description of development of text messaging survey). The adaption process included a local terminology study on diarrhoea and pneumonia signs and symptoms (Online Supplementary Document(Online Supplementary Document), Local terminology study) and cognitive interview study on understanding of the text message questions by caregivers (Online Supplementary Document(Online Supplementary Document), Guide for cognitive interviews). We used the final face–to–face and text messaging survey questions for the study (see Table S6 in Online Supplementary Document(Online Supplementary Document)).

In the *questionnaire on reasons for different responses*, the questionnaire (Online Supplementary Document(Online Supplementary Document), Questionnaire for interviews about reasons for different responses) included two questions to help the participant think about why they gave a different answer. The third question, “Why do you think the answer you gave for this question via text message is different from the response you gave face–to–face?”, had the following answer options: misunderstood text message question, misunderstood face–to–face question, changed mind, put wrong answer in text message, gave no response to text message question, or other.

***7. Data collection:*** We addressed the schedule of the fieldwork and procedures for data collection.

*Fieldwork schedule:* In group 1, first the interviewers obtained informed consent and interviewed the participants face–to–face on the demographic, mobile phone use and care–seeking for childhood diarrhoea and pneumonia; then after one day the text messages were sent. The one–day period between the surveys was a balance between memory and recall issues. This period was introduced so that participants were likely to have forgotten their answers, but still had a similar survey 2–week recall.

In group 2, on the first day we obtained informed consent, asked participants the demographic and mobile phone use questions and informed participants that they received text messages after 2 days. On the third and fourth day, the text message survey took place. There were two days between the informed consent and the first text message for logistical reasons, because the follow–up interviews could not coincide with the recruitment days (it was not feasible to do both recruitment and follow–up interviews on the same day). On the fifth day, we visited villages for the second time to ask the survey questions face–to–face. We sent a text message to participants and asked them to come to the clinic for the face–to–face interview on care–seeking for childhood diarrhoea and pneumonia. We only asked participants who responded to the first diarrhoea module question to participate in the face–to–face interview, because we aimed to compare the text messaging and face–to–face responses.

*Procedures:* In the *face–to–face survey on demographics and mobile phone use*, the interviewers administered the demographic questions and mobile phone use after participants signed the informed consent. They paid special attention to correctly recording the mobile phone numbers of participants, because it was essential for the study to have the correct mobile phone numbers. The interviewers called the participants on the mobile phone number they provided to validate the number (if the participant brought a mobile phone). Interviewers recorded responses of participants with a smartphone.

In the *face–to–face survey on care–seeking for childhood diarrhoea and pneumonia*, interviewers also recorded responses to the diarrhoea module and cough and fever module of participants with a smartphone. As required for the face–to–face interviews, the interviewers did not give the answers to participants, but selected the most appropriate answer based on the participant’s response.

In the *text message survey on care–seeking for childhood diarrhoea and pneumonia*, we sent the first text message (introduction text message which did not require a response) at 9 am in the morning and the second text message that asked about their willingness to participate directly after the first text message. We called participants who responded that they were not willing to participate and asked for their reasons. If the reason was that they misunderstood the question, we gave an explanation and asked them to reply again. When a participant was willing to participate, we sent the third text message with a question about the identity of the participant. We checked whether the identity of the participant was identical to the identity of the person who signed the informed consent and who participated in the face–to–face interview. When the identity was different, we called the mobile phone number and the person answering the phone was asked for their identity. If the person was related to the child on the name list, we asked the person to encourage the person who signed the informed consent and participated face–to–face to reply to the text messages.

The researcher sent the first survey question to all participants who were willing to respond and who were the same caregiver that participated face–to–face. First, we sent questions from the diarrhoea module; this included text messages 4 to 13. Second, we sent the cough and fever module; this included text messages 14 to 20. When we received a response to the text message question, we sent the appropriate follow–up question until the text message survey was completed. We followed the survey algorithm for sending the appropriate follow–up questions. Depending on the condition of the child, certain questions had to be answered or could be skipped. When participants completed the survey, then we sent an end text message and thanked participants for their cooperation. We sent text messages till 9 pm in the evening (Online Supplementary Document(Online Supplementary Document), Descriptions of sending text messages).

Participants were required to respond with an answer in Chinese characters. The answer options of the questions were provided in the text messages, because previous research showed that not giving participants the answer options resulted in unclear answers. We considered asking participants to reply with a number, but this was inconvenient and some participants ignored our request. However, this meant that we anticipated some unclear answers.

When a text message was empty we sent the following response: “your text message is empty” followed by the text message with question. When an answer phrase was unclear we sent the following text message: “there is a problem with your text message, please respond again” followed by the text message with question. When there was a question from the participant, we called the participant. When participants said that they did not want to continue, we sent them the following text message: “We are sorry to hear you want to discontinue, you will not receive text messages from us anymore. Thank you for participating.” and stopped sending text messages.

**Figure 5:**
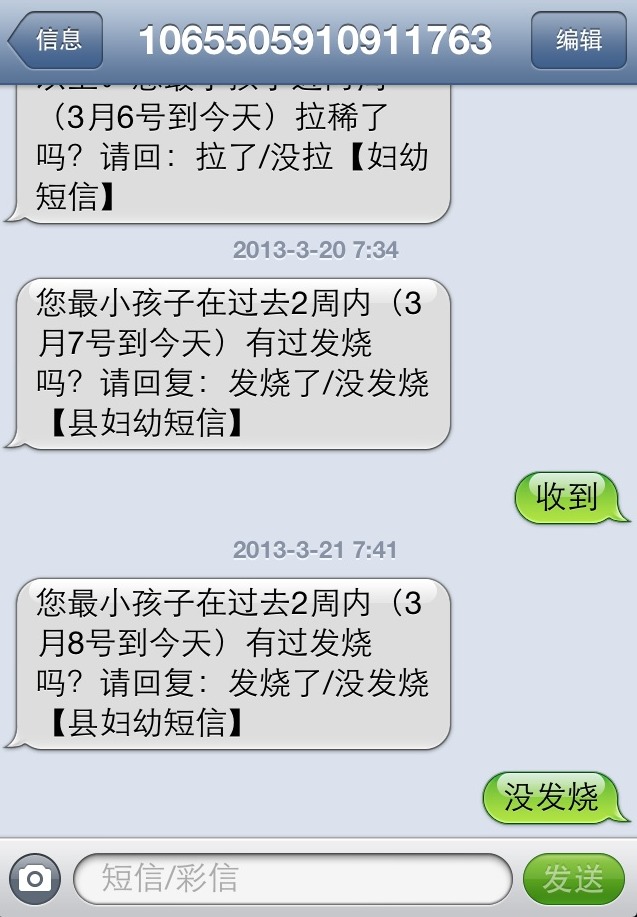
Screen shot of mobile phone. A screen shot of a supervisor’s mobile phone when testing sending of text messages in the morning before the fieldwork of the cross–over study started. The shot is courtesy of Xiaozhen Du, personal collection.

The number of the text messaging system contained 16 numbers (1065–5059–1091–1763). This was a special number, because normal Chinese mobile phone numbers have only 11 digits without area codes. We checked the functionality of the text messaging system during the fieldwork. We asked the text message system company for a report of successfully sent text messages. Also, every morning before sending text messages, we sent text messages to eight mobile phone numbers of researchers in our team, which included the three major telecom operators in China, China mobile, China Unicom, and China Telecom, and asked them to reply (**Figure 5**). If we did not receive their reply, we made phone calls to the researchers to confirm whether they had received a text message.

In *interviews on participants’ reasons for differences in responses*, at the end of the face–to–face interviews in group 2, we asked participants who participated in both the text messaging and face–to–face interview structured questions about reasons for giving a different response when comparing the face–to–face and text messaging answers. Before the face–to–face interview, the first text messaging researcher (YL) sent the responses of the text messages to the supervisors in the field (WW and XD). Directly after the face–to–face interview, the interviewers compared the responses to the face–to–face questions and text message question and marked differences in responses. The supervisors conducted the interviews and recorded one of the different answer options with pen–and–paper.

***8. Data analysis and outcomes:*** We used SPSS version 16.0 [77] and SAS version 9.2 for the statistical analysis of the quantitative data. We compared characteristics between groups 1 and 2 with the Pearson chi–square test and Fisher Exact Test for nominal variables and Mann-Whitney U/ Wilcoxon W test for not normally distributed continuous variables and ordinal variables. We considered *P* values less than 0.05 significant. We present data from the diarrhoea and cough and fever questions for all participants in group 1 and for those who returned to the village clinic and completed the modules in group 2. We did not impute missing data.

*Data equivalence and the amount of information:* We assessed data equivalence (intrarater agreement); the degree to which the responses to the face–to–face questions and text messages were identical [85]. Kappa is a useful statistic for measuring agreement and to test measurement equivalence [86]. Cohen’s kappa can be used to indicate the strength of agreement for a nominal scale used on separate occasions [87]. Cohen’s kappa compares the observed agreement with agreement that is expected by chance alone, which makes it a chance–corrected index of agreement. A kappa value of 0 means that there is no agreement beyond chance, while a kappa value of 1 indicates that there is perfect agreement. There is no accepted standard for rating the different values for kappa. Kappa values higher than 0.60, 0.70 or 0.80 are generally considered to be the minimum standard for group–level comparisons or for research purposes. However, these strengths of agreement do not indicate the practical relevance of results. There are a number of interpretations, which all are arbitrary [88,89]. We used the Landis and Koch [88] interpretation, because it has the most detailed description of agreement: <0.00 poor, 0.00–0.20 slight, 0.21–0.40 fair, 0.41–0.60 moderate, 0.61–0.80 substantial, and 0.81–1.00 almost perfect.

Disagreements between different ratings are not equally important for ordinal data. To account for this, Cohen introduced weights for the calculation of a weighted kappa [90]. Weighted kappa takes account of the distance between disagreements and is therefore appropriate for ordinal scales with more than two categories. There are different weights given to kappa, but the most commonly used ones are the Cicchetti–Allison [91] and Fleiss–Cohen weights [92]. Fleiss–Cohen gives quadrant weights and can be similar to the intraclass correlation coefficient [92,93]. Cicchetti–Allison gives linear weights and is more appropriate for questions with many answer options [94]. The linearly weighted kappa coefficient can be simply derived from K–1 embedded 2x2 classification tables [95]. Our survey included two ordinal questions, with five answer options. The value of weighted kappa is sensitive to the choice of weights [96]. As the number of answer options was relatively high, Cicchetti–Allison was the most appropriate choice for the weights. However, also this is arbitrary and therefore we presented both Fleiss–Cohen and Cicchetti–Allison weights.

Our survey on care–seeking for childhood diarrhoea and pneumonia included 17 questions that could be compared between the two methods: (i) 10 questions with a nominal scale (dichotomous “yes” or “no” answers); (ii) 5 questions with a nominal non–dichotomous scale for which we calculated kappa values when possible; and (iii) 2 questions with an ordinal scale (answers varying from “none” to “more”) for which we calculated weighted Cicchetti–Allison and Fleiss–Cohen weights kappa values. We calculated the results for group 1 and 2 combined and compared of kappa values and 95% confidence intervals (CI) for the two groups separately.

In addition, we analysed the amount of information by the number of places caregivers reported for question 13 and 20 (places where caregivers sought care) by comparing the number of places given between the face–to–face method and the text message method.

We reported a combination of kappa statistics (including kappa values, 95% Confidence Intervals (CI), *P* values) and the percentage of agreement, which allowed for a detailed impression of data agreement [97]. For the proportion of agreement, we did not present the proportion when the number of participants was less than ten.

*Participants’ reasons for differences in responses:* We calculated proportions for the reasons for differences between face–to–face and text messaging responses.

*Item response rate and overall response rate:* We defined item response rate as the proportion of participants responding to each question and the overall response rate as the proportion of participants who completed the text messaging survey. For the proportion of participants completing the survey, the number of questions participants had to answer depended on the responses that they gave to questions about the condition of their child. There were five conditions that determined the questions participants had to answer: diarrhoea, complementary feed, fever, cough and fast or difficult breathing. We created 24 “statuses” that represent all combinations of these 5 conditions. We calculated the number of participants completing each of the 24 different statuses. However, we could only calculate the proportion of participants that completed the entire survey for all of the 24 different statuses combined. We could not calculate proportions for each different status, because participants who did not reply did not provide information on their status, and therefore could not be classified.

*Characteristics of responders vs non–responders:* In group 1 we compared responders with non–responders for the first question and for the complete survey. We used the same tests as we used for the comparison of characteristics between group 1 and group 2.

*Error rate of the text messaging method:* We evaluated the error rate of the text messaging method by incorrect text message questions that we sent and incorrect text message answers that we received from participants. For the face–to–face method, this was not relevant, because the smartphone was programmed to avoid errors. The smartphone guided the interviewer through the interview and when a value was missing or out of range, the interviewer could not continue the survey without entering a valid response. However, despite our efforts to minimize errors in the text messaging survey, this could not be eliminated due to the manual process of sending text messages. We defined incorrect questions sent as text messages that were not sent or not sent in the right format because of researcher errors. We defined incorrect text message answers as responses of the participants that were empty, unclear, or out of range and that had to be assessed, and those that needed a follow–up text message. We presented an overview of the text messages sent and received with proportions of incorrect text message questions sent and answers received.

## CONCLUSION

This paper described the objectives, field site and methods of a large mHealth project that is part of collaboration between researchers in China and the UK. The mixed methods study evaluating factors that influence sample size calculations could help future studies with estimating reliable sample sizes. The cross–over study comparing face–to–face and text message survey data collection could help future studies with developing their mHealth tools.
