# Supplementary material for: mHealth Series: mHealth project in Zhao County, rural China – Description of objectives, field site and methods
Source: J Glob Health. 2013 Dec;3(2):020401. doi: 10.7189/jogh.03.020401 (PMC3868818; doi:10.7189/jogh.03.020401)
Supplement: Online Supplementary Document [file jogh-03-020401-s001.pdf]

Online Supplementary Document

van Velthoven et al. mHealth series paper 1, mHealth project in Zhao County, rural China: Description of objectives, field site and methods.
Journal of Global Health 2013;3:020401

Contents

Field site photographs 3

Study 1: Factors influencing sample size calculations for mHealth-based health surveys, a mixed methods study 5

Part 1: Surveys 5

Survey 1 on demographics and mobile phone use - English version 5

Survey 1 on demographics and mobile phone use - Chinese version 14

Survey 2 on demographics and mobile phone use - English version 26

Survey 2 on demographics and mobile phone use - Chinese version 30

Part 2: Interviews 33

Topic guides for semi-structured interviews with caregivers 33

Topic guide for semi-structured interviews with village doctors 38

Questionnaires for interviews with caregivers participating in the mHealth cross-over study 40

Study 2: Comparison of text messaging vs. face-to-face interviews for health surveys, a cross-over study 45

Detailed description of development of text messaging survey 45

Figure S1 Flow diagram of question development 53

Table S1 Overview of questions during development of face-to-face and text message survey 54

Table S2 Text messages with changes marked green, based on cognitive interviews round 1 (C1) 74

Table S3 Text messages with changes marked blue, based on cognitive interviews round 2 day 1 (C2.1) 85

Table S4 Text messages with changes marked pink, based on cognitive interviews round 2 day 2 (C2.2) 92

Table S5 Text messages with changes marked red, based on pilot 98

Table S6 Face-to-face and text message questions used in cross-over study 105

Local terminology study 116
Table S7 Results of step 1: assess current information on local terms 129
Table S8 Results of step 2: validate local terms through interviews with caregivers 135
Table S9 Results of step 3: select local terms to adapt the questionnaire 136
Guide for cognitive interviews 137
Questionnaire for interviews about reasons for different responses 140
Description of sending text messages 141
References 142

Field site photographs

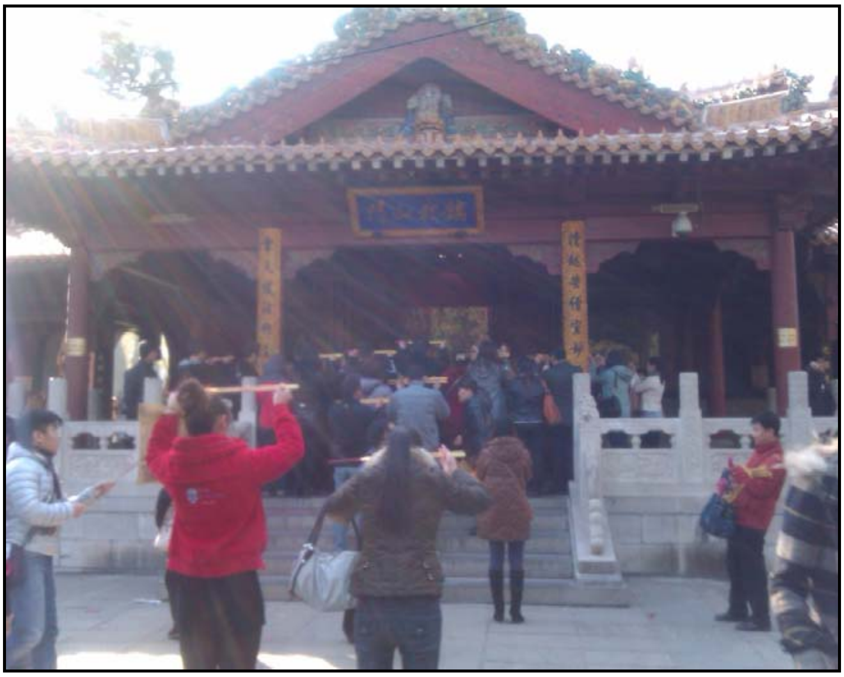

People praying at Bailin (Cypress Grove) Temple 柏林禅寺

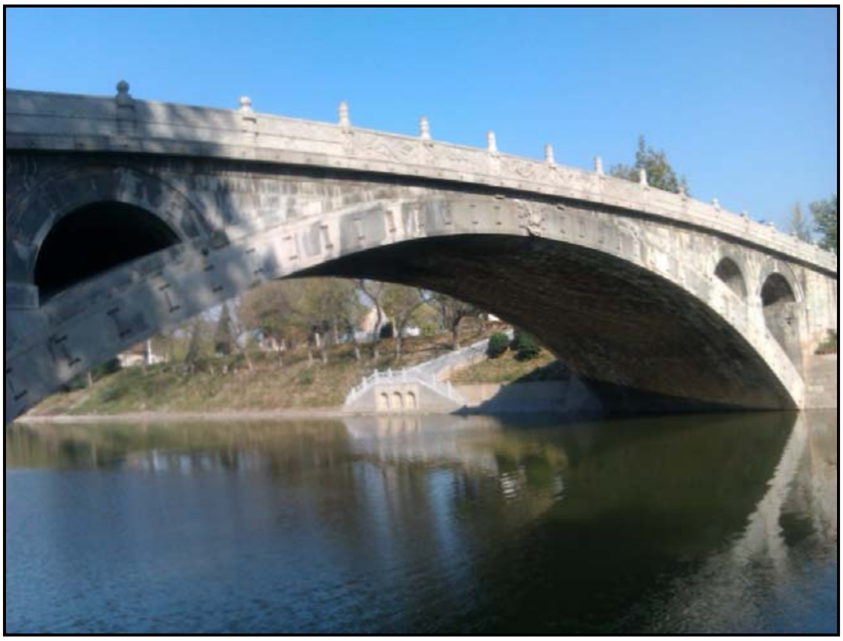

Zhaozhou “Arch” (Anji) Bridge 赵州桥

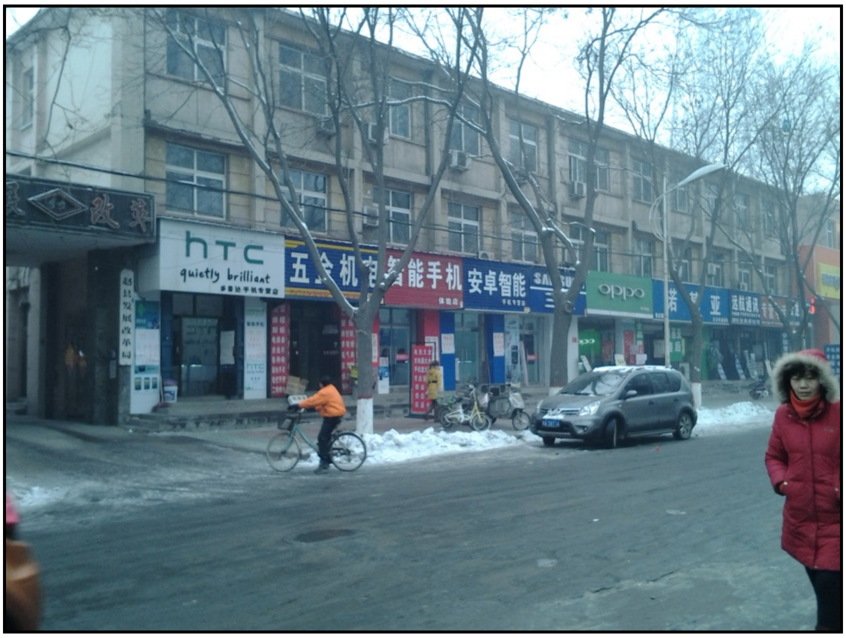

Street in Zhaozhou city

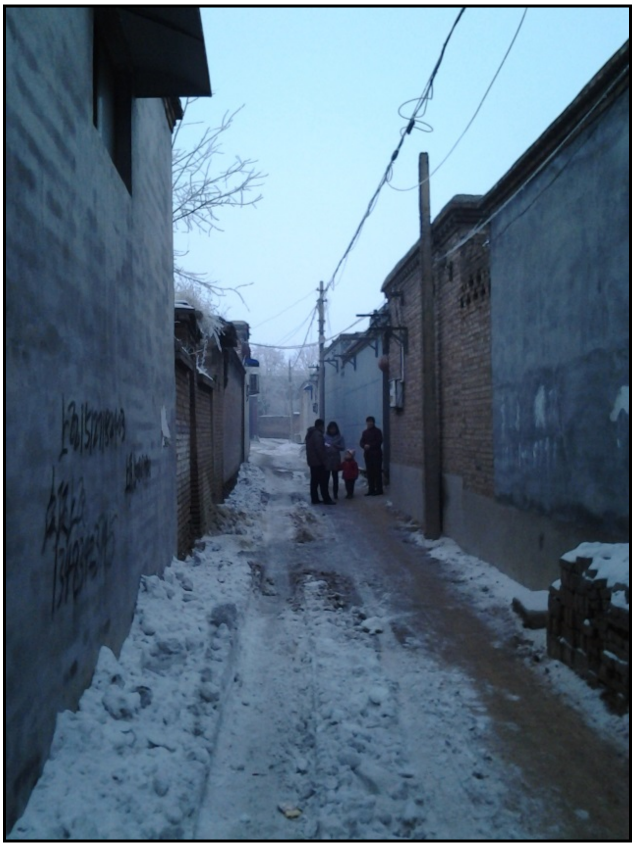

Street in rural Zhao County

Photographs: the courtesy of Michelle Helena van Velthoven, personal collection

Study 1: Factors influencing sample size calculations for mHealth-based health surveys, a mixed methods study

Part 1: Surveys

Survey 1 on demographics and mobile phone use - English version

Question nr	Question	Answer options	Referral to other questions
**Identification (ID)**			
ID1a	The name (code) of the township	_________	
ID1b	The name (code) of the village	_________	
ID1c	The ID of the child	_________	
ID2	The name (code) of the interviewee	_________	
ID3	Relationship to the child	1.Mother<br>2.Father<br>3.Grandfather/Grandmother<br>8.Other____	
ID4	Who is the primary caregiver for this child?	1.Mother<br>2.Father<br>3.Grandfather/Grandmother<br>8.Other____	
ID5	The name of the child *(whose age is 6-23 months)*		
ID6	Gender of the child	1.Male<br>2.Female	
ID7	Date of birth of the child *(solar calendar; yyyy/mm/dd)*	________	
ID8	Weight at birth	________g<br>8888=Do not know	
ID9	Length at birth	________cm<br>88=Do not know	
ID10	How many pregnancies has the mother of the child had, up to and including this child?	8=Do not know	
ID10a	How many children has the mother had before this child? *(fill in "7" if there are more than 7 children)*	___________<br>8=Do not know	
ID10b	Is the child a twin/triplet etc.?	1.No<br>2.Twin<br>3.Triplet or more<br>8.Do not know	

ID11	What was the method of delivery for this child?	1.Vaginal delivery<br>2.Delivery with instrumental assistance<br>3.Caesarean section<br>8.Do not know	
ID12	Gestation period for this child in weeks *(round down to nearest week)*	______________<br>88.Do not know	
ID13	Type of Hukou *(household register)*	1.Urban<br>2.Rural	
ID14	Investigator ID	____________	
ID15	Date of the investigation *(solar calendar; yyyy/mm/dd)*	____________	
**Mobile phone (MP) use**			
MP1	Do you use a mobile phone?	1.Yes<br>2.No	If MP1=2 go to MP17
MP2	What is the brand and type of the mobile phone you use?	___________________<br>8.Do not know	
MP3	Is the mobile phone you use a smartphone? *(demonstrate a smartphone, based on Android, Symbian or iOS system)*	1.Yes<br>2.No<br>8.Do not know	
MP4	What is the usual location of the mobile phone you use, when you leave the house?	1.Carry the phone with me<br>2.Leave the phone in the house<br>3.Someone else carries the phone<br>8.Other____	
MP5	Do you primarily use a mobile phone for making calls or sending text messages?	1.Making calls<br>2.Sending text messages<br>3.Both in equal measure<br>8.Other____	
MP5a	Do you primarily send text messages or use QQ?	1.QQ<br>2.Text messages<br>3.Both in equal measure<br>4.Cannot use neither<br>5.Other____<br>8.Do not know	
MP6	How many phone calls do you make in an average week?	__________________<br>88=Do not know<br>99=Cannot do it	
MP7	How many phone calls do you receive in an average week?	__________________<br>88= Do not know<br>99=Cannot do it	

MP8	How many text messages do you sent in an average week?	___________________<br>88=Do not know<br>99=Cannot do it
MP9	How many text messages do you receive in an average week?	___________________<br>88=Do not know<br>99=Cannot do it
MP10	How much is your average mobile phone bill in one month?	___________________<br>888=Do not know
MP11	How often did you change your mobile phone number in the last year?	1.Never<br>2.Once<br>3.Twice<br>4.Three times<br>5.Four times or more<br>8.Do not remember
MP12	Is your mobile phone currently functioning correctly i.e. can it be used for making a phone call and sending a text message? *(including a mobile phone without sufficient credit)*	1.The phone can be used to make a call and send a text message<br>2.The phone can be used to make a call but cannot send a text message<br>3.The phone can be used to send a text message, but cannot make a call<br>4.The phone can neither send a text message nor make a phone call<br>5.Other____
MP13	Do you or anyone in your household have the telephone number of the following *(including mobile number)*:	
	1.County hospital or above *(including county hospital, county children's hospital, private hospital)*	1.Yes<br>2.No<br>8.Do not know
	2.Township hospital	1.Yes<br>2.No<br>8.Do not know
	3.Village clinic	1.Yes<br>2.No<br>8.Do not know

MP14	How often have you used your mobile phone to receive information about your own health or your child's health in the past 3 months? *(including text message and calls)*	1.Never<br>2.Once<br>3.Twice<br>4.Three times<br>5.More than three times	If MP14=1, go to MP15<br>If MP14=2, 3,4 or 5, go to MP16
MP15	Would you like to use your mobile phone to receive health information?	1.Yes<br>2.No<br>3.Other____	
MP16	Where do you usually buy your mobile phone?	1.In the village<br>2.In the town<br>3.In the county<br>4.In the city<br>5.Other____<br>8.Do not know	
MP17	Does anyone else in your household use a mobile phone?	1.Yes<br>2.No, only I have a mobile phone<br>3.No, nobody in our household has got a mobile phone<br>8.Do not know	If MP17=3, go to QQ1
MP18a	Regarding the child's mother: Does she use a mobile phone?	1.Yes<br>2.No<br>8.Do not know	If MP18a=2 or 8, go to MP19a
MP18b	Who owns the mobile phone?	1=Herself<br>2=Father<br>3=Grandmother<br>4=Grandfather<br>5=Other____	If MP18b=2 ,3,4,5 go to MP18d1
MP18c	Mobile phone number	________________________	
MP18d1	Does she know how to make a phone call with a mobile phone?	1.Yes<br>2.No<br>8.Do not know	
MP18d2	Does she know how to send a text message?	1.Yes<br>2.No<br>8.Do not know	
MP19a	Regarding the child's father: Does he use a mobile phone?	1.Yes<br>2.No<br>8.Do not know	If MP19a=2 or 8, go to MP20a

MP19b	Who owns the mobile phone?	1=Mother<br>2=Himself<br>3=Grandmother<br>4=Grandfather<br>5=Other____	If MP19b=1, 3, 4, or 5 then go to MP19d1
MP19c	Mobile phone number	________________________	
MP19d1	Does he know how to make a phone call with a mobile phone?	1.Yes<br>2.No<br>8.Do not know	
MP19d2	Does he know how to send a text message?	1.Yes<br>2.No<br>8.Do not know	
MP20a	Regarding the grandmother:<br>Does she use a mobile phone?	1.Yes<br>2.No<br>8.Do not know	If MP20a=2 or 8, go to question MP21a
MP20b	Who owns the mobile phone?	1=Mother<br>2=Father<br>3=Herself<br>4=Grandfather<br>5=Other____	If MP20b=1, 2, 4, or 5 then go to MP20d1
MP20c	Mobile phone number	________________________	
MP20d1	Does she know how to make a phone call with a mobile phone?	1.Yes<br>2.No<br>8.Do not know	
MP20d2	Does she know how to send a text message?	1.Yes<br>2.No<br>8.Do not know	
MP21a	Regarding the grandfather:<br>Does he use a mobile phone?	1.Yes<br>2.No<br>8.Do not know	If MP21a=2 or 8, go to MP22a
MP21b	Who owns the mobile phone?	1=Mother<br>2=Father<br>3=Grandmother<br>4=Himself<br>5=Other____	If MP21b=1, 2, 3, or 5 go to MP21d1
MP21c	Mobile phone number	________________________	
MP21d1	Does he know how to make a phone call with a mobile phone?	1.Yes<br>2.No<br>8.Do not know	

MP21d2	Does he know how to send a text message?	1.Yes<br>2.No<br>8.Do not know	
MP22a	Are there other adult household members who have interacted frequently with the child?	1.Yes<br>2.No<br>8.Do not know	If MP22a=2 or 8, go to QQ1
MP22b	How does the child refer to him/her?	______________________	
MP22c	Does she/he use a mobile phone?	1.Yes<br>2.No<br>8.Do not know	If MP22c=2 or 8, go to QQ1
MP22d	Who owns the mobile phone?	1=Mother<br>2=Father<br>3=Grandmother<br>4=Grandfather<br>5=Him/herself<br>6=Other____	If MP22d=1, 2, 3, 4 or 6 go to MP22f1
MP22e	Mobile phone number	_______________________	
MP22f1	Does she/he know how to make a phone call with a mobile phone?	1.Yes<br>2.No<br>8.Do not know	
MP22f2	Does she/he know how to send a text message?	1.Yes<br>2.No<br>8.Do not know	
**Use of internet (QQ)**			
QQ1a	Do you use your computer in your household to access the Internet?	1.Yes<br>2.No<br>8.Do not know	
QQ2	Do you use your mobile phone to access the Internet?	1.Yes<br>2.No<br>3.Do not have a mobile phone<br>8.Do not know	
**Household (HH)**			
HH4a	Is it possible to collect information about the mother?	1.Yes<br>2.No, remarried<br>3.No, lost<br>4.No, passed away	
HH4	What is the age of the mother?	__________________years<br>88=Do not know	

HH6	What is the mother's education level?	1.No education<br>2.Primary school<br>3.Junior high school<br>4.Senior high school/technical school<br>5.Vocational/technical secondary school<br>6.College<br>7.University or above<br>8.Do not know
HH7	How many years of education has the mother had?	___________________years
HH8	What is the mother's occupation?	1.Housework<br>2.Head of an enterprise, organisation or business unit<br>3.Technical<br>4.Receptionist, clerk, secretary<br>5.Commercial, business and service industry<br>6.Owner of individual business<br>7.Farmer engaged in non-agricultural industry<br>8.Industrial workers (factory production worker or transporter, mining, construction) with non-agricultural Hukou<br>9. Agriculture, forestry, or fishing water conservation<br>10.Military<br>11.Other____<br>88.Do not know
HH10a	Is it possible to collect information about the father?	1.Yes<br>2.No, remarried<br>3.No, lost<br>4.No, passed away
HH10	What is the age of the father?	___________________years<br>88=Do not know

HH12	What is the father's education level?	1.No education<br>2.Primary school<br>3.Junior high school<br>4.Senior high school/technical school<br>5.Vocational/technical secondary school<br>6.College<br>7.University or above<br>8.Do not know
HH12a	How many years of education has the father had?	__________________years
HH13	What is the father's occupation?	1.Housework<br>2.Head of an enterprise, organisation or business unit<br>3.Technical<br>4.Receptionist, clerk, secretary<br>5.Commercial, business and service industry<br>6.Owner of individual business<br>7.Farmer engaged in non-agricultural industry<br>8.Industrial workers (factory production worker or transporter, mining, construction) with non-agricultural Hukou<br>9. Agriculture, forestry, or fishing water conservation<br>10.Military<br>11.Other____<br>88.Do not know
HH16a	What is the age of the primary caregiver? *Only ask if the caregiver is not the mother or the father.*	__________________years

HH16b	What is the caregiver's education level?	1.No education<br>2.Primary school<br>3.Junior high school<br>4.Senior high school/technical school<br>5.Vocational/technical secondary school<br>6.College<br>7.University or above<br>8.Do not know
HH16c	How many years' of education does the caregiver have?	__________________years
HH16d	What is the caregiver's occupation?	1.Housework<br>2.Head of an enterprise, organisation or business unit<br>3.Technical<br>4.Receptionist, clerk, secretary<br>5.Commercial, business and service industry<br>6.Owner of individual business<br>7.Farmer engaged in non-agricultural industry<br>8.Industrial workers (factory production worker or transporter, mining, construction) with non-agricultural Hukou<br>9. Agriculture, forestry, or fishing water conservation<br>10.Military<br>11.Other____<br>88.Do not know
HH20	Family net income in the last year *(after subtracting cost of production)*	________Yuan<br>888888=Do not know
HH21	Family living expenses in the last year *(living expenses include food, clothing, daily consumable, transport, communication, mortgage/rent, household bills, education, cultural entertainment, hospital bills etc.)*	________Yuan<br>888888=Do not know

Survey 1 on demographics and mobile phone use - Chinese version

家庭编号信息（**ID)**

**ID.1a**	乡（镇）/街道：____________	乡（镇）/街道编号<br>**ID.1a□**
**ID.1b**	村/居委会：____________	村/居委会编号<br>**ID.1b□□**
**ID.1c**	儿童编号____________	儿童编号 **ID.1c□□**
**ID.2**	被访者姓名：____________	**ID.2**
**ID.3**	与儿童的关系：	**ID.3□**
	1.母亲 2.父亲 3.（外）祖父母 8．其他：______	
**ID.4**	儿童主要养育人是谁？	**ID.4.1□**
	1.母亲 2.父亲 3.（外）祖父母 8．其他：______	
**ID.5**	家中6-23月儿童的姓名：___________________	**ID.5**
**ID.6**	该儿童的性别：1.男 2.女	**ID.6□**
**ID.7**	该儿童的出生日期（阳历）：<br>__ __ __ __年__ __月__ __日	**ID.7** □□□□ __ _
**ID.8**	出生体重：__ __g (500g=1斤,50g=1两, *不知道=8888*)	**ID.8□□□□**
**ID.9**	出生身长：__ __.__cm（*不知道=88*）	**ID.9□□**
**ID.10**	生这个孩子是妈妈第几次怀孕？<br>__ *次（如果≥7次，填"7，8.不知道）*	**ID.10□**
**ID.10a**	妈妈之前生过几个孩子？<br>__ *次（如果≥7次，填"7，8.不知道）*	**ID.10a□**

**ID.10b**	这个孩子是双胞胎或者多胞胎吗？	**ID.10b□**
	1. 否(单胎) 2.双胞胎 3.三胞胎及以上 8.不知道	
**ID.11**	母亲生这个孩子的分娩方式？	**ID.11□**
	1.阴道分娩 2.器械助产 3.剖宫产 8.不知道	
**ID.12**	这个孩子是怀了多久生的？（例: 38周5天记为38周）<br>____周（88.不知道）	**ID.12□□**
**ID.13**	孩子的户口类型？ 1.城镇户口 2.非城镇户	**ID.13□**
**ID.14**	调查员编号___________	调查员编号<br>**ID.14□□**
**ID.15**	调查日期：__ __ __ __年__ __月__ __日	**ID.15□□□□5__ __**

手机使用偏好(MP)

**MP.1**	您平时用手机吗？	**MP.1□**
	1. 用 2. 不用———**转到 MP.17**	
**MP.2**	这个手机是什么品牌和型号的？	**MP.2**
	品牌+型号____________（*8=不知道*）	
**MP.3**	您用的手机是智能手机吗？（展示手机，解释基于安卓、塞班、iOS 系统）	1. **MP.3□**
	1.是 2.否 8.不知道	**2.**
**MP.4**	当您外出时，您平时用的这个手机经常放在什么地方？	**3. MP.4□**
	1. 自己随身携带 2. 放在家<br>3. 其他人带着手机 4. 其他___________	**4.**
**MP.5**	您主要是用手机打电话还是发短信？	**MP.5□**
	1.电话 2. 短信 3. 差不多 4.其他_________	

**MP.5a**	您平常习惯使用手机上QQ还是发短信？	**MP.5a**□
	1.QQ 2.短信 3.差不多 4.都不会用 5.其他_________ 8.不知道	
**MP.6**	您平均每周拨出电话的数量？ ______ (88.不知道 99.不会打电话)	**MP.6**□□
**MP.7**	您平均每周接到电话的数量？ ______ (88.不知道 99.不会接电话)	**MP.7**□□
**MP.8**	您平均每周发出短信的数量？ ______ (88.不知道 99.不会发短信)	**MP.8**□□
**MP.9**	您平均每周收到短信的数量？ ______ (88.不知道 99.不会收短信)	**MP.9**□□
**MP.10**	您用的这部手机平均每个月的话费是多少钱？<br>_________元（888.不知道）	**MP.10**□<br>□□
**MP.11**	您过去一年更换过几次电话号码？	**MP.11**□
	1. 没有换过 2. 换过1次 3. 换过2次 4.换过3次<br>5. 换过4次及以上 8. 不记得	
**MP.12**	现在手机是否能正常使用？（指手机的性能,包括因欠费导致的功能不正常）	**MP.12**□
	1.能打电话和发短信 2.仅能打电话不能发短信 3.仅能发短信不能打电话 4.不能打电话也不能发短信 5.其他状况_________	

**MP.13**	您家里面有人有下列人员的的电话号码吗？（包括座机和手机）		
	1.县级医院或以上<br>（包括县级医院，县妇幼保健院，私立医院）	1．是 2.否 8.不知道	**MP.13.a** □□
	2.乡镇卫生院	1．是 2.否 8.不知道	**MP.13.b** □□
	3.村卫生室	1．是 2.否 8.不知道	**MP.13.c** □□
**MP.14**	在过去的 3 个月中，您有几次通过手机得到关于您自己或孩子健康方面的相关信息？（短信和电话都算）		**MP.14**□
	1.从来没有过 2.一次— **转到 MP.16** 3. 两次——两**转到 MP.16**<br>4. 三次—— **转到 MP.16** 5.大于三次——大**转到 MP.16**		
**MP.15**	你是否愿意通过手机获得健康信息？		**MP.15**□
	1.是 2.否 3.其他		
**MP.16**	您一般在哪里购买手机？		**MP.16**□
	1.村附近 2.镇上 3.县城里 4.市区 5.其他_______ 6.不知道		
**MP.17**	您家里的其他人平时用手机吗？		**MP.17**□
	1. 用 2.家里只有我用手机 3 家里所有人都不用手机—— **转到 QQ.1**<br>8.不知道其他人的状况		

**MP.18.a**	母亲有用手机的习惯吗？		**MP.18.a** □
	1．有 2.没有——没**转到MP.19.a** 8.不知道—— **转到 MP.19a**		
**MP.18.b**	母亲使用的是谁的手机？		**MP.18.b** □
	1.母亲 2.父亲— **转到MP.18.d1** 3. 奶奶（姥姥）— **转到 MP.18.d1**<br>4.爷爷（姥爷）—**转到MP.18.d1** 5.其他— **转到 MP.18.d1**		
**MP.18.c**	母亲手机的电话号码 (88. 不知道)		**MP.18.c** □□□□□□□□□□□
	母亲能够用手机完成		
**MP.18.d1**	打电话	1．能 2.不能 8.不知道	**MP.18.d1**□
**MP.18.d2**	发短信	1．能 2.不能 8.不知道	**MP.18.d2**□
**MP.19.a**	父亲有用手机的习惯吗？		**MP.19.a** □
	1．有 2.没有——**转到MP.20.a** 8.不知道——**转到 MP.20.a**		

**MP.19.b**	父亲使用的是谁的手机？		**MP.19.b** □
	1.母亲—**.转到MP.19.d** 2.父亲<br>3. 奶奶（姥姥）—**转到MP.19.d1** 4.爷爷（姥爷）—**转到MP.19.d**1<br>5.其他—**转到MP.19.d1**		
**MP.19.c**	父亲手机的电话号码 (88. 不知道)		**MP.19.c** □□□□□□□□□□□
	父亲能够用手机完成		
**MP.19.d1**	打电话	1．能 2.不能 8.不知道	**MP.19.d1**□
**MP.19.d2**	发短信	1．能 2.不能 8.不知道	**MP.19.d2**□
**MP.20.a**	**奶奶/姥姥**有用手机的习惯吗？		**MP.20.a** □
	1．有 2.没有——没**转到MP.21.a** 8.不知道——**转到MP.21.a**		
**MP.20.b**	奶奶/姥姥使用的是谁的手机？		**MP.20.b** □
	1. 母亲—**.转到MP.20.d1** 2.父亲— **转到MP.20.d1** 3.奶奶（姥姥）<br>4.爷爷（姥爷）—**转到MP.20.d1** 5.其他—**转到MP.20.d1**		

**MP.20.c**	奶奶/姥姥所使用手机的电话号码 （88.不知道）		**MP.20.c**<br>□□□□□□□□□□□
	奶奶/姥姥能够用手机完成		
**MP.20.d1**	打电话	1．能 2.不能 8.不知道	**MP.20.d1**□
**MP.20.d2**	发短信	1．能 2.不能 8.不知道	**MP.20.d2**□
**MP.21.a**	**爷爷/姥爷**有用手机的习惯吗？		**MP.21.a**<br>□
	1．有 2.没有——**转到MP.22.a** 8.不知道——**转到MP.22.a**		
**MP.21.b**	爷爷/姥爷使用的是谁的手机？		**MP.21.b**<br>□
	1.母亲—**转到MP.21.d1** 2.父亲— **转到MP.21.d1**<br>3.奶奶（姥姥）—.**转到MP.21.d1**） 4.爷爷（姥爷）5.其他—.**转到MP.21.d1**		
**MP.21.c**	爷爷/姥爷所使用手机的电话号码 （88. 不知道）		**MP.21.c**<br>□□□□□□□□□□□
	爷爷/姥爷能够用手机完成		
**MP.21.d1**	打电话	1．能 2.不能 8.不知道	**MP.21.d1**□
**MP.21.d2**	发短信	1．能 2.不能 8.不知道	**MP.21.d2**□

**MP.22.a**	家中还有其他大人和孩子长期在一起吗？		**MP.22.a**<br>□
	1.有 2.没有——没**转到QQ.1** 8.不知道——**转到QQ.1**		
**MP.22.b**	孩子对他/她的称呼是？ ________ 88.不知道		**MP.22.b**
**MP.22.c**	他/她有用手机的习惯吗？		**MP.22.c**<br>□
	1．有 2.没有——没**转到QQ.1** 8.不知道——8**转到QQ.1**		
**MP.22.d**	他/她使用的是谁的手机？		**MP.22.d**<br>□
	1.母亲—**转到MP.22.f1** 2.父亲— **转到MP.22.f1**<br>3.奶奶（姥姥）—**转到MP.22.f1** 4.爷爷（姥爷）—姥**转到MP.22.f1**<br>5.自己 6.其他—.**转到MP.22.f1**		
**MP.22.e**	他/她所使用手机的电话号码 （88. 不知道）		**MP.22.e**<br>□□□□□□□□□□□
	他/她能够用手机完成		
**MP.22.f1**	打电话	1．能 2.不能 8.不知道	**MP.22.f1**<br>□
**MP.22.f2**	发短信	1．能 2.不能 8.不知道	**MP.22.f2**<br>□

QQ使用偏好(QQ)

**QQ.1a**	你用家里电脑上网吗？	**QQ.1a□**
	1.用<br>2.不用<br>8.不知道	
**QQ.2**	你用手机上网吗？	**QQ.2□**
	1.用<br>2.不用<br>3.没有手机<br>8.不知道	

家庭基本信息（**HH)**

**HH.4a**	是否可以记录母亲信息		**HH.4a□**
	1.可以 2.再婚**——** ***转到HH.10a***<br>3.下落不明**——*不转到HH.10a*** 4.去世**——*.转到 HH.10 a***		
**HH.4**	母亲的年龄：__ __周岁 （88-不知道）		**HH.4a□□**
**HH.6**	母亲的文化程度		**HH.6□**
	1.没上过学**——*没转到HH.8***<br>2.小学<br>3.初中<br>4.高中/技校	5. 中专/中技<br>6. 大专<br>7. 大学以上<br>8. 不清楚	
**HH.7**	母亲上了多少年学？		**HH.7□□**
	__ __ 年 （0.没有上过学，88. 不知道）		

<table>
<tr><td>HH.8</td><td colspan="2">母亲的工作</td><td>HH.8□</td></tr>
<tr><td></td><td>1.家务
2.机关、企事业单位负责人
3.专业技术人员
4.一般办事人员
5.商业服务业人员
6.个体工商户</td><td>7.从事非农劳动的农民
8.非农业户口的产业工人
9. 农林牧渔水利业生产人员
10.军人
11.其他
____
88.不知道</td><td></td></tr>
<tr><td>HH.10a</td><td colspan="2">是否可以记录父亲信息</td><td>HH.10a□</td></tr>
<tr><td></td><td colspan="2">1.可以　　　　　2.再婚—— 转到 HH.15
3.下落不明——不转到 HH.15　4.去世——.转到 HH.15</td><td></td></tr>
<tr><td>HH.10</td><td colspan="2">父亲年龄 __ __周岁（88-不知道）</td><td>HH.10□□</td></tr>
<tr><td>HH.12</td><td colspan="2">父亲的文化程度</td><td>HH.12□</td></tr>
<tr><td></td><td>1.没上过学——没转到 HH.13
2.小学
3.初中
4.高中/技校</td><td>5. 中专/中技
6. 大专
7. 大学以上
8. 不清楚</td><td></td></tr>
<tr><td>HH.12a</td><td colspan="2">父亲上了多少年学？</td><td>HH.12a□□</td></tr>
<tr><td></td><td colspan="2">__ __ 年（0.没有上过学，88. 不知道）</td><td></td></tr>
</table>

<table>
<tr><td>HH.13</td><td colspan="2">父亲的工作</td><td>HH.13□</td></tr>
<tr><td></td><td>1.家务
2.机关、企事业单位负责人
3.专业技术人员
4.一般办事人员
5.商业服务业人员
6.个体工商户</td><td>7.从事非农劳动的农民
8.非农业户口的产业工人
9. 农林牧渔水利业生产人员
10.军人
11.其他____________________
88.不知道</td><td></td></tr>
<tr><td>HH.16a</td><td colspan="2">父母以外的主要养育人年龄？ __ __ 周岁</td><td>HH.16a□□</td></tr>
<tr><td>HH.16b</td><td colspan="2">父母以外的主要养育人的文化程度</td><td>HH.16b□</td></tr>
<tr><td></td><td>1.没上过学——没转到 HH.16d
2.小学
3.初中
4.高中/技校</td><td>5. 中专/中技
6. 大专
7. 大学以上
8. 不知道</td><td></td></tr>
<tr><td>HH.16c</td><td colspan="2">父母以外的主要养育人上了多少年学？</td><td>HH.16c□□</td></tr>
<tr><td></td><td colspan="2">__ __ 年 （0.没有上过学，88. 不知道）</td><td></td></tr>
<tr><td>HH.16d</td><td colspan="2">父母以外的主要养育人的职业</td><td>HH.16d□</td></tr>
<tr><td></td><td>1.家务
2.机关、企事业单位负责人
3.专业技术人员
4.一般办事人员
5.商业服务业人员
6.个体工商户</td><td>7.从事非农劳动的农民
8.非农业户口的产业工人
9. 农林牧渔水利业生产人员
10.军人
11.其他____________________
88.不知道</td><td></td></tr>
</table>

**HH.20**	您的家庭去年纯收入为（元）__ __ __ __ __ __（*家庭去年扣去生产成本后各种收入的总和*）不知道填”888888”	**HH.20**□□□□□□
**HH.21**	您的家庭去年生活消费性支出为__ __ __ __ __ __（元）<br><br>不知道填”888888”<br><br>（生活性消费支出包括食品、衣着、日用品、交通、通讯、住房、水电及燃料、教育、文化娱乐、医疗等方面的支出）	**HH.21**□□□□□□

Survey 2 on demographics and mobile phone use - English version

Nr	Question	Answer options
**Identification (ID)**		
ID1a	The name (code) of the village	________
ID1c	The ID of the child	________
ID2	The name (code) of the interviewee	________
ID3	Relationship to the child	1.Mother<br>2.Father<br>3.Grandfather<br>4.Grandmother<br>5.Other____
ID3a	What is your mobile phone number?	_________________
ID.4	Is each family member one of the main caregivers? *Read selections one by one and record all of them.*	
ID.4.1	The mother?	1.Yes<br>2.No
ID.4.2	The father?	1.Yes<br>2.No
ID.4.3	The grandmother?	1.Yes<br>2.No
ID4.4	The grandfather?	1.Yes<br>2.No
ID4.5	Anyone else?	1.Yes<br>2.No
ID5	The name of the youngest child	______________
ID6	Gender of the child	1.Male<br>2.Female
ID7	Date of birth for the child *(solar calendar; yyyy/mm/dd)*	______________
ID10a	How many children has the mother had before this child? *(fill in "7" if there are more than 7 children)*	______________<br>8=Do not know
ID10b	Is the child a twin/triplet etc.?	1.No<br>2.Twin<br>3.Triplet or more<br>8.Do not know
ID13	Type of Hukou *(household register)*	1.Urban<br>2.Rural<br>8.Do not know
ID14	Investigator ID	______________
ID15	Date of the investigation	______________

**Mobile phone (MP) use**		
MP3	Is the mobile phone you use a smartphone? *(demonstrate a smartphone, based on Android, Symbian or iOS system)*	1.Yes<br>2.No<br>8.Do not know
MP5	Do you primarily use a mobile phone for making calls or sending text messages?	1.Making calls<br>2.Sending text messages<br>3.Both in equal measure<br>8.Other____
MP5a	Do you usually primarily send text messages or use QQ?	1.QQ<br>2.Text messages<br>3.Both in equal measure<br>4.Use neither<br>5.Other____<br>8.Do not know
MP6	How many phone calls do you make in an average week?	___________________<br>888=Do not know<br>999=Cannot do it
MP7	How many phone calls do you receive in an average week?	___________________<br>888=Do not know<br>999=Cannot do it
MP8	How many text messages do you sent in an average week?	___________________<br>888=Do not know<br>999=Cannot do it
MP9	How many text messages do you receive in an average week?	___________________<br>888=Do not know<br>999=Cannot do it
MP13	Do you or anyone in your household have the telephone number of the following *(including mobile number).*	*Read selections one by one and record all of them.*
MP13.1	County hospital or above *(including county hospital, county children's hospital, private hospital)*	1.Yes<br>2.No<br>8.Do not know
MP13.2	Township hospital	1.Yes<br>2.No<br>8.Do not know
MP13.3	Village clinic	1.Yes<br>2.No<br>8.Do not know
MP14	How many times have you used your mobile phone to receive information about your own health or your child's health in the past 3 months? *(including text message and telephone) If MP14=1, go to MP15*<br>*If MP14=2,3,4 or 5, go to HH4*	1.Never<br>2.Once<br>3.Twice<br>4.Three times<br>5.More than three times
MP15	Would you like to use your mobile phone to receive health information?	1.Yes<br>2.No<br>3.Other____

Household (HH)		
HH4	What is the age of the mother?	___________________years
HH6	What is the mother's education level?	1.No education<br>2.Primary school<br>3.Junior high school<br>4.Senior high school/technical school<br>5.Vocational/technical secondary school<br>6.College<br>7.University or above<br>8.Do not know
HH7	How many years of education has the mother had?	___________________years
HH8	What is the mother's occupation?	1.Housework<br>2.Head of an enterprise, organisation or business unit<br>3.Technical<br>4.Receptionist, clerk, secretary<br>5.Commercial, business and service industry<br>6.Owner of individual business<br>7.Farmer engaged in non-agricultural industry<br>8.Industrial workers (factory production worker or transporter, mining, construction) with non-agricultural Hukou<br>9. Agriculture, forestry, or fishing water conservation<br>10.Military<br>11.Other____<br>88.Do not know
HH10	What is the age of the father?	___________________years
HH12	What is the father's education level?	1.No education<br>2.Primary school<br>3.Junior high school<br>4.Senior high school/technical school<br>5.Vocational/technical secondary school<br>6.College<br>7.University or above<br>8.Do not know
HH12a	How many years of education has the father had?	___________________years

HH13	What is the father's occupation?	1.Housework<br>2.Head of an enterprise, organisation or business unit<br>3.Technical<br>4.Receptionist, clerk, secretary<br>5.Commercial, business and service industry<br>6.Owner of individual business<br>7.Farmer engaged in non-agricultural industry<br>8.Industrial workers (factory production worker or transporter, mining, construction) with non-agricultural Hukou<br>9. Agriculture, forestry, or fishing water conservation<br>10.Military<br>11.Other____<br>88.Do not know
HH20	Family net income in the last year *(after subtracting cost of production)*	________Yuan<br>888888=Do not know
HH21	Family living expenses in the last year *(living expenses include food, clothing, daily consumable, transport, communication, mortgage/rent, household bills, education, cultural entertainment, hospital bills etc.)*	________Yuan<br>888888=Do not know

Survey 2 on demographics and mobile phone use - Chinese version

家庭编号信息 (ID)

**ID.1a**	村/居委会：____________		村/居委会编号 **ID.1a□□**
**ID.1c**	儿童编号____________		儿童编号 **ID.1c□□**
**ID.2**	被访者姓名：____________		**ID.2**
**ID.3**	与儿童的关系：		**ID.3□**
	1. 母亲 2.父亲 3.（外）祖母 4.（外）祖父 5. 其他：______		
**ID.3a**	被访者手机号码：__ __ __ __ __ __ __ __ __ __ __		**ID.3a**
**ID.4**	下列家庭成员是儿童的主要喂养人吗？*逐项读出，并记录每一个选项及答案。*		
	1. 母亲	1.是 2.否	**ID.4.1□**
	2. 父亲	1.是 2.否	**ID.4.2□**
	3. （外）祖母	1.是 2.否	**ID.4.3□**
	4. （外）祖父	1.是 2.否	**ID.4.4□**
	5. 其他__________	1.是 2.否	**ID.4.5□**
**ID.5**	最小儿童姓名：		**ID.5□**
**ID.6**	该儿童的性别：1.男 2.女		**ID.6□**
**ID.7**	该儿童的出生日期（阳历）：__ __ __ __年__ __月__ __日		**ID.7□□□□□□□□/□□**
**ID.10a**	妈妈之前生过几个孩子？__ 次（*如果≥7个，填"7"，8.不知道*）		**ID.10a□**
**ID.10b**	这个孩子是双胞胎或者多胞胎吗？		**ID.10b□**
	1. 否(单胎) 2.双胞胎 3.三胞胎及以上8.不知道		
**ID.13**	孩子的户口类型？ 1.城镇户口 2.非城镇户口 8.不知道		**ID.13□**
**ID.14**	调查员编号____________		**ID.14□□**
**ID.15**	调查日期：__ __ __ __年__ __月__ __日		**ID.15 __ __□D.1**

手机使用偏好（MP）

**MP.3**	您用的手机是智能手机吗？（展示手机，解释基于安卓、塞班、iOS 系统）	**MP.3□**
	1.是 2.否 8.不知道	
**MP.5**	您主要是用手机打电话还是发短信？	**MP.5□**
	1.电话 2. 短信 3. 差不多 4.其他__________	
**MP.5a**	您平常习惯使用手机上QQ还是发短信？	**MP.5a□**
	1.QQ 2.短信 3.差不多 4.都不用 5.其他__________ 8.不知道	
**MP.6**	您平均每周拨出电话的数量？______ (888.不知道 999.不会打电话)	**MP.6□□**

MP.7	您平均每周接到电话的数量？______ (888.不知道 999.不会接电话)		MP.7□□
MP.8	您平均每周发出短信的数量？______ (888.不知道 999.不会发短信)		MP.8□□
MP.9	您平均每周收到短信的数量？______ (888.不知道 999.不会收短信)		MP.9□□
MP.13	您家里面有人有下列人员的的电话号码吗？（包括座机和手机）***逐项读出，并记录每一个选项及答案***		
	1 县级医院或以上<br>（包括县级医院，县妇幼保健院，私立医院）	1．是 2. 否 8.不知道	MP.13.1□
	2.乡镇卫生院	1．是 2. 否 8.不知道	MP.13.2□
	3.村卫生室	1．是 2. 否 8.不知道	MP.13.3□
MP.14	在过去的 3 个月中，您有几次通过手机得到关于您自己或孩子健康方面的相关信息？（短信和电话都算）		MP.14□
	1．从来没有过　2. 一次—>**转下一部分** 3. 两次—>**转下一部分**<br>4. 三次—>**转下一部分** 5. 大于三次—>**转下一部分**		
MP.15	你是否愿意通过手机获得健康信息？		MP.15□
	1.是　2.否　3.其他		

家庭基本信息 (HH)

HH.4	母亲的年龄：__ __周岁		HH.4□□
HH.6	母亲的文化程度		HH.6□
	1.没上过学<br>2.小学<br>3.初中<br>4.高中/技校	5. 中专/中技<br>6. 大专<br>7. 大学以上<br>8. 不清楚	
HH.7	母亲上了多少年学？		HH.7□□
	__ __ 年 （0.没有上过学，88. 不知道）		
HH.8	母亲的工作		HH.8□□
	01.家务<br>02.机关、企事业单位管理者<br>03.专业技术人员<br>04.一般办事人员<br>05.商业服务业人员<br>06.个体工商户	07.从事非农劳动的农民<br>08.非农业户口的产业工人<br>09. 农林牧渔水利业生产人员<br>10.军人<br>11.其他______________<br>88.不知道	
HH.10	父亲年龄 __ __周岁		HH.10□□

**HH.12**	父亲的文化程度		**HH.12□**
	1.没上过学<br>2.小学<br>3.初中<br>4.高中/技校	5. 中专/中技<br>6. 大专<br>7. 大学以上<br>8. 不清楚	
**HH.12a**	父亲上了多少年学？		**HH.12a□□**
	__ __ 年（0.没有上过学，88. 不知道）		
**HH.13**	父亲的工作		**HH.13□□**
	01.家务<br>02.机关、企事业单位管理者<br>03.专业技术人员<br>04.一般办事人员<br>05.商业服务业人员<br>06.个体工商户	07.从事非农劳动的农民<br>08.非农业户口的产业工人<br>09. 农林牧渔水利业生产人员<br>10.军人<br>11.其他__________<br>88.不知道	
**HH.20**	您的家庭去年纯收入为（元）__ __ __ __ __ __（*家庭去年扣去生产成本后各种收入的总和*）不知道填"888888"		**HH.20□□□□□□**
**HH.21**	您的家庭去年生活消费性支出为__ __ __ __ __ __（元）（生活性消费支出包括食品、衣着、日用品、交通、通讯、住房、水电及燃料、教育、文化娱乐、医疗等方面的支出）不知道填"888888"		**HH.21□□□□□□**

Part 2: Interviews

Topic guides for semi-structured interviews with caregivers

Topic guide 1 (first round of interviews)

**Research questions**
1. How do people use a mobile phone?   2. What is people's experience with seeking information for their child's health?

GENERAL SECTION.

To start off, I would like to find out a little about you and your family:

1. What do you usually do during the day?
2. Can you please describe your family members and their relationship to you?
3. Who will take care of the child when he or she is ill?

SECTION 1.

We will continue to talk about your mobile phone use:

4. Can you show me your mobile phone? *(if participant cannot show the mobile phone ask what sort of mobile phone it is)* Do you use any other mobile phones?
5. Where do you keep your mobile phone?
6. Why do you use a mobile phone?

7. Which functions of the mobile phone do you use?
8. Which do you prefer: sending text messages or making phone calls? Why?
9. Sometimes people have problems with using their mobile phone. For example a phone stops working, someone deletes a text message accidently by pressing the wrong button, or someone realizes that there is not enough credit on the phone. Have you ever seen something like this happening?
10. How much money do you spend on using your mobile phone?
11. How long have you used a mobile phone?
12. How often do you change your mobile phone number? Why?
13. Did you purchase this mobile phone? If not, who purchased the mobile phone? On what occasion did he or she give you the mobile phone?
14. Can you tell me about other people in your household using a mobile phone?

Before I ask you more questions, I just want to briefly sum what you told me about using your mobile phone. You said………………, is that right?

SECTION 2.

I would now like to ask you a bit about seeking information or advice when your child has a health problem:

15. Who do you first ask for help when your child has a health problem?
16. Have you sought any information about your child's health from a doctor working in a village clinic or township hospital in the past year? Please tell me about the most recent time you sought information about your child's health.

17. Have you contacted a doctor working in a village clinic or township hospital by a mobile phone in the past year? Please tell me about your most recent contact with a health worker via your mobile phone.
18. Do you use health-related applications on your mobile phone? If so, please tell me more about this.
19. Would you be interested in participating in follow-up research? *(if so, please record contact details carefully)*

Before we end this interview, I just want to briefly sum what you told me about seeking health information. You said……………… , is that right?

Thank you very much for taking part (stress confidentiality and anonymity again).

Topic guide 2 (second round of interviews)

**Research questions**
1. Which factors influence whether people respond to text messages?<br>2. What is people's experience with seeking information for their child's health via their mobile phone?

SECTION 1.

We will talk about your mobile phone use:

1. When did you use a mobile phone for the first time?
2. Why do you use a mobile phone?
3. Which functions of the mobile phone do you use?
4. Which function do you like best? Why?
5. Now I would like to talk more about mobile phone text messaging. How long does it normally take for you to notice receiving a text message?
6. When you notice you have received a text message, when will you respond to it?
7. What kind of messages do you respond to? What kind of messages do you ignore? Why?
8. Have you ever received information or questions via mobile phone text messages? If so, please tell me more about this.

Before I ask you more questions, I just want to briefly sum what you told me about using your mobile phone and text messaging. You said……………., is that right?

SECTION 2.

I would now like to ask you a bit about seeking information or advice when your child has a health problem:

9. Who do you first ask for help when your child has a health problem?
10. Have you sought any information about your child's health from a doctor working in a village clinic or township hospital in the past year? Please tell me about the most recent time you sought information about your child's health.
11. Have you contacted a doctor working in a village clinic or township hospital by a mobile phone in the past year? Please tell me about your most recent contact with a health worker via your mobile phone.
12. Do you use health-related applications on your mobile phone? If so, please tell me more about this.
13. Have you sought any health information on the internet? If so, please tell me more about this.
14. Would you be interested in participating in follow-up research? *(if so, please record contact details carefully)*

Before we end this interview, I just want to briefly sum what you told me about seeking health information. You said……………., is that right?

Thank you very much for taking part <u>(stress confidentiality and anonymity again).</u>

Topic guide for semi-structured interviews with village doctors

Research questions:

1. How many caregivers are village doctors able to find?
2. What motivates village doctors to find caregivers?
    b. How much money do they want to receive for finding caregivers? (amount of money per number of caregivers)
    c. What are other ways to motivate village doctors?
3. What factors influence the number of caregivers coming to the village clinic?

SECTION 1.

We will talk a little bit in general about your village clinic:

1. Can you tell me a little bit about your village and your village clinic?
2. What are the biggest problems for the health of young children in your village?

SECTION 2.

I would now like to ask you about asking caregivers to participate in research:

3. Can you give an estimation of how many people in your village have a child younger than five? How many of those people with a young child do you know?

4. What do you think about our research? Why?
5. How do you find helping with research?
6. How did you find asking caregivers to participate in our research?
7. What tasks do you like/dislike to do for research?
8. How did the amount of money you received from us compared to the amount of time you had to work? How does it compare to your income?
9. How could we make it easier for you to find more caregivers?
10. What do you think the main problems are with finding caregivers? Why do you think that is?
11. How do you think we could find more caregivers? What motivates caregivers to come to the village clinic? What makes you say that?

Thank you very much for taking part** **<u>(stress confidentiality and anonymity again).</u>

Questionnaires for interviews with caregivers participating in the mHealth cross-over study

1. Participants who completed the text message survey

Questions:

1. How did you find replying to text messages questions about whether your child was ill in the past 2 weeks?
2. You have participated in the survey about your child's health face-to-face and via text messaging. Which method of answering questions about your child's health do you prefer? (face-to-face, text message, other____) Why? What makes you say that? *Dig deeper into true preferences*
3. What is your preferred time for receiving text messages? (time) Were the times (*state the times here*) that the messages were sent to you acceptable? (yes or no and why)
4. Reminders:
    a. Did you receive any reminders? (yes, no, not sure) How many? (number)
    b. How did you feel about the number of reminders you received? (ok with the number of reminders received, received too many reminders, would have liked more reminders, other____)
5. You received 1 Yuan for your text message fees and 5 Yuan for completing the survey.

a. How did you feel about the amount of money for the text message fees, which is 1 Yuan? (was enough, was not enough, was too much, other___) Why? What makes you say that?

b. How did you feel about the amount of money for completing the survey, which is 5 Yuan? (was enough, was not enough, was too much, other___) Why? What makes you say that?

c. You received a small gift (towel) for participating in the face-to-face survey. What is your preference: receiving 5 Yuan mobile phone credit, a gift, or health information for a survey? (5 Yuan mobile phone credit, small gift, health information, other___) Why? What makes you say that?

6. How many messages with questions about your child's health are you willing to answer at most on one day? (1-2, 3-4, 5-6, 7-8, >8)

7. How many messages with questions about your child's health are you willing to answer in total per survey? (1-2, 3-4, 5-6, 7-8, >8)

8. If we ask about your child's health regularly, how often would you accept this? (once a month, once every two months, once every three months, once every 6 months, once a year)

9. Do you have any other comments? (yes_______,no)

2. Participants who responded to at least one text message question

Questions:

1. You responded to (*insert number of text messages which were responded to*) text message questions from us. You did not reply to the (*insert the number and question of text message which was not responded to*) text message question.
    a. Did you receive this question via a text message? (yes, no, not sure)
    b. Did you receive text message reminders? (yes, no, not sure)
    c. If no or not sure, why do you think you did not receive any messages? (changed the mobile phone number, mobile phone was broken, other____)
2. Why did you stop responding to the text messages questions? (did not bring the phone, phone was powered off, did not have enough credit, did not have time, did not know how to reply, forgot to reply, did not find it useful, did not trust the message, afraid of charging fees, afraid of advertising messages, other____)
3. How did you find responding to the text message(s)?
4. You have participated in the survey about your child's health face-to-face and via text messaging. Which method of answering questions about your child's health do you prefer? (face-to-face, text message, other____) Why? What makes you say that?

5. What would need to change for you, so that you would respond to all text message survey questions about your child's health?
6. You received 1 Yuan for your text message fees.
   a. How did you feel about the amount of money for the text message fees, which is 1 Yuan? (was enough, was not enough, was too much, other____) Why? What makes you say that?
   b. You would have received 5 Yuan for completing the survey. How do you feel about the amount of money for completing the survey, which is 5 Yuan? (was enough, was not enough, was too much, other____) Why? What makes you say that?
   c. You received a small gift (towel) for participating in the face-to-face survey. What is your preference: receiving 5 Yuan mobile phone credit, a gift, or health information for a survey? (5 Yuan mobile phone credit, small gift, health information, other____) Why? What makes you say that? Do you have any other comments? (yes____,no)

3. Participants who did not respond to any text message questions

Questions:

1. Receiving messages:
    a. Did you receive a text message from us? (yes, no, not sure)
    b. Did you receive any reminder messages? (yes, no, not sure) If no or not sure, why do you think you did not receive any messages? (changed mobile phone number, mobile phone was broken, replied but did not receive a reply from us, other____)
2. Why did you not respond to the text messages questions about whether your child was ill in the past two weeks? (did not bring the phone, phone was powered off, did not have enough credit, did not have time, did not know how to reply, forgot to reply, did not find it useful, did not trust the message, afraid of charging fees, afraid of advertising messages, other____)
3. What would need to change for you, so that you would respond to text message survey questions about your child's health?
4. Do you have any other comments?

Study 2: Comparison of text messaging vs. face-to-face interviews for health surveys, a cross-over study

Detailed description of development of text messaging survey

Overview of methods

The survey questions about caregiver's care-seeking behaviour for diarrhoea and pneumonia were based on the diarrhoea module and cough and fever module (used to assess pneumonia) of the World Health Organization (WHO) maternal, newborn and child health (MNCH) survey (Table S1; column headed "Existing WHO MNCH survey"). These questions are similar to the key questions in the Demographic and Health Surveys (DHS) and the Multiple Indicator Cluster Surveys (MICS), which are used in other parts of the world. However, China does not use these surveys, but instead uses its own tools such as the National Health Services Survey (NHSS). The Capital Institute of Pediatrics in Beijing has used the WHO MNCH survey since 2010 (draft version October 2009; unpublished). We selected the most relevant questions from the modules to ask about care-seeking for diarrhoea and suspected pneumonia. In the paragraphs below, we explain how the WHO MNCH survey was adapted to the local context in Zhao County and converted into text message questions; this is illustrated in seven steps in a question development flow diagram (Figure S1). We show an overview of the questions with the changes for each phase in colour (Table S1). We explain the reasons for changes for each phase (Table S2-S5) and present the final questions that we used (Table S6).

Local adaptation of face-to-face questions (step 1)

Some changes and additions were made to the survey based on experiences with conducting the survey in China since 2010 (Table S1; column headed “CIP survey” with blue marks). A number of questions asked the caregiver to recall the past two weeks. To clarify this, we added an extra phrase “please think back from two weeks ago till today” to help caregivers recall information. For question DI.3.a. (drinking during diarrhoea), the answer option “much less or none” was split into “none” and “much less”, and the answer option “about the same or somewhat less” was split into “about the same” and “somewhat less”, because participants indicated that there was a difference between these options. In addition, “illness” was changed into “diarrhoea” to clarify for which illness drinking behaviour was asked. For question DI.3.b (eating during diarrhoea) the answer options “much less” and “none” were merged into “much less or none”, because participants indicated that this was the same. Question DI.4., “Did you seek advice or treatment for the diarrhoea outside the home?”, was changed into “Did you seek advice or treatment for the diarrhoea?”. Also, in the first answer option “outside the home” was added, because participants felt this was clearer. For question DI.4.a (“Why didn’t you seek advice?”) answer option six “not allowed to go” was changed into “not necessary”, because no caregivers said that they were not allowed to go, but some said that it was not necessary to go. For question DI.4.b (“Where did you seek care?”), the answer options were made specific to Zhao County. Similar changes were made for the cough and fever module questions. In addition, we adapted the questionnaire modules on diarrhoea and cough and fever to include local terms (Local terminology study).

Conversion of face-to-face survey questions into text message questions (step 2)

We converted the face-to-face survey questions from the WHO MNCH survey into text messages between July and December 2012. Chinese child survey researchers (YZ, LC, QW, YL and WW), a child health researcher (IR) and mHealth researchers (JC and MV) were involved in this process. The following changes were made to the text messages that we used for the first cognitive interview round (Table S1; column headed "Cognitive interviews 1" with yellow marks). We added an introduction text message to introduce the survey and we added health information to clarify the meaning of the questions. We merged the three questions for question DI.3. (drinking during diarrhoea) to decrease the number of text messages. For the face-to-face interviews this question was asked in three parts, but we thought this could be merged and asked caregivers to identify all the liquids their child drunk in one question. For question CO.2 (illness caused by cough), in the face-to-face survey question CO.2.a (fast or difficult breathing) was not asked when the answer to CO.2 was "no". However, we decided to let participants answer question CO.2.a when the answer to CO.2 was "no" to be able to ask this question to more participants. In question DI.4.b, we changed "seek care" into "seek advice or treatment" to be consistent with previous questions in the survey. The text messages were translated by a Chinese researcher (YL) and checked by a second Chinese researcher (WW). The translations were compared and discussed with a researcher fluent in English (MV). The final translation was checked by the bilingual (Chinese-English) translator (EC).

Text message questions for cognitive interviews (step 3, 4, and 5)

The current survey was an interviewer-administered face-to-face survey, which was different from the self-administered text messaging survey in which caregivers answered questions on their own. Therefore, we had to check understanding of the meaning of the questions by caregivers and cognitive interviewing techniques and usability testing are appropriate for this purpose [1]. Cognitive interviewing is a technique that allows evaluating potential questions and confirming validity of data being collected. This facilitated evaluation of caregivers' understanding of the meaning of questions and checking that meanings were existent across caregivers [2].

We tested the converted survey questions using cognitive interviews to check if the interpretation of the survey questions was similar to different caregivers (Table 1; column headed "Cognitive interviews 1" with yellow marks). A Chinese interviewer (YL) asked caregivers to participate in the interviews and conducted the interviews. The researcher fluent in English (MV) with experience in interviewing trained YL. The aim of the interviews was to better understand: (i) caregivers' understanding of the meaning of questions in text message format, (ii) caregivers' understanding of how to respond to the questions, and (iii) whether information should be sent in one or two text messages. The interview guide included questions around the meaning and understanding of the questions (Guide for cognitive interviews) and was tested in November 2012. We sent text messages to participants during the interview and asked them to respond straight away. Text messages which could have information and a question together were randomly sent separately and together, and we asked

participants which way they preferred. We asked participants to describe in their own words what the information, questions and answer options meant, and to rephrase the content in their own words. We wrote the answers of participants on a standardised sheet including caregiver's understanding of the content, rephrased content in their own words, reply to the text message, experiences and feelings, and notes. In addition, we asked caregivers questions about their willingness to respond to the text messages. We used an interview approach similar to the semi-structured interviews (described in the section "interviewers and data collection" in the methods section of the main article). We specifically probed on the changes that we made in the questions during different cognitive interview rounds.

We planned to interview five to ten caregivers per round of interviews. In the first cognitive interview round (C1), we interviewed eight caregivers during five days. After the interviews, YL and MV analysed the results and discussed the changes that had to be made with the rest of the study team (Table S2). In the second round, we interviewed eight caregivers on two days. On the first day (C2.1), we interviewed four caregivers with the revised questions from the first cognitive interview round. We discussed the findings of the interviews, made changes in the text messages and wrote down reasons for making the changes (Table S3). On the second day (C2.2), we interviewed another four caregivers with the revised questions from the first day. We ended the interviews when we felt that the text messages were understandable and no new issues came up during the interviews. We revised the text messages based on our findings and discussions within the team (Table S4).

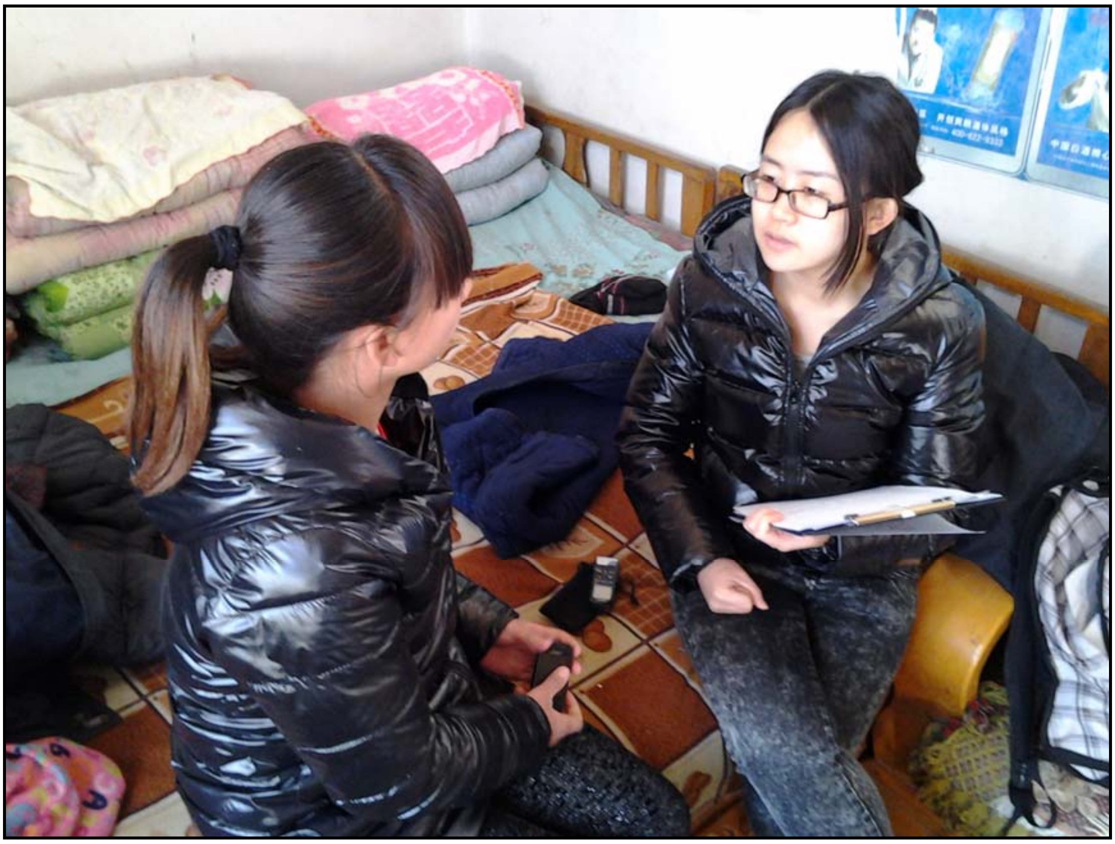

Mother and interviewer during cognitive interview.

Photograph: the courtesy of Michelle Helena van Velthoven, personal collection

Text message questions for pilot (step 6)

We sent the text messages that we revised after the cognitive interviews rounds (Table S4) to a sample of 217 caregivers from Shahedian Township in Zhao County to test sending the text messages. Of those 217 caregivers who we sent text messages to, 88 responded, 86 agreed to participate and 24 completed the survey. Five caregivers had a child with diarrhoea and ten caregivers had a child with fever or cough. We found some additional issues that had to be addressed and made changes in the text messages accordingly (Table S5). One important issue that we found was that no caregivers (out of five participants who had a child with diarrhoea) responded to the second diarrhoea question (blood in diarrhoea); also not after we sent a reminder text message. When we called participants, they said that they had not received the text message. We found that the text messages were not sent, because the text message contained the word "blood", which blocked sending the text messages with the text messaging system. We did not find this earlier in the cognitive interviews, because we then used a mobile phone to sent text messages. We asked the major telecommunication operators in China, China Mobile, China Telecom and China Unicom, for their lists of blocked words, checked them and found no additional words in our text messages that were blocked. Another issue was that the text messaging system could not sent some of the text messages in one text message, because they were too long, despite that we carefully checked that the information in the text messages was less than 65 Chinese characters. We made minor changes to those text messages to make the information fit into one text message.

Text message questions and face-to-face questions for cross-over study (step 7)

We made adjustments based on findings from the pilot and we developed the final questions for the face-to-face and text message surveys (Table S6). Between the pilot and the cross-over study, we were unable to interview caregivers face-to-face and therefore we interviewed caregivers through phone calls. We tested the adjustments (Table S5) by sending text messages with an adjustment to four caregivers and called them to ask about the meaning of the question, and their experiences and feelings when responding to the text messages.

We intended to use the face-to-face questions that we used in Zhao County in January 2013. However, as we made modifications to the text message questions, we also had to make minor modifications to the face-to-face questions to ensure consistency between the face-to-face and text messaging survey (Table S1; column headed “Cross-over study survey” with brown marks). These changes were only made for consistency and did not change the meaning of the questions.

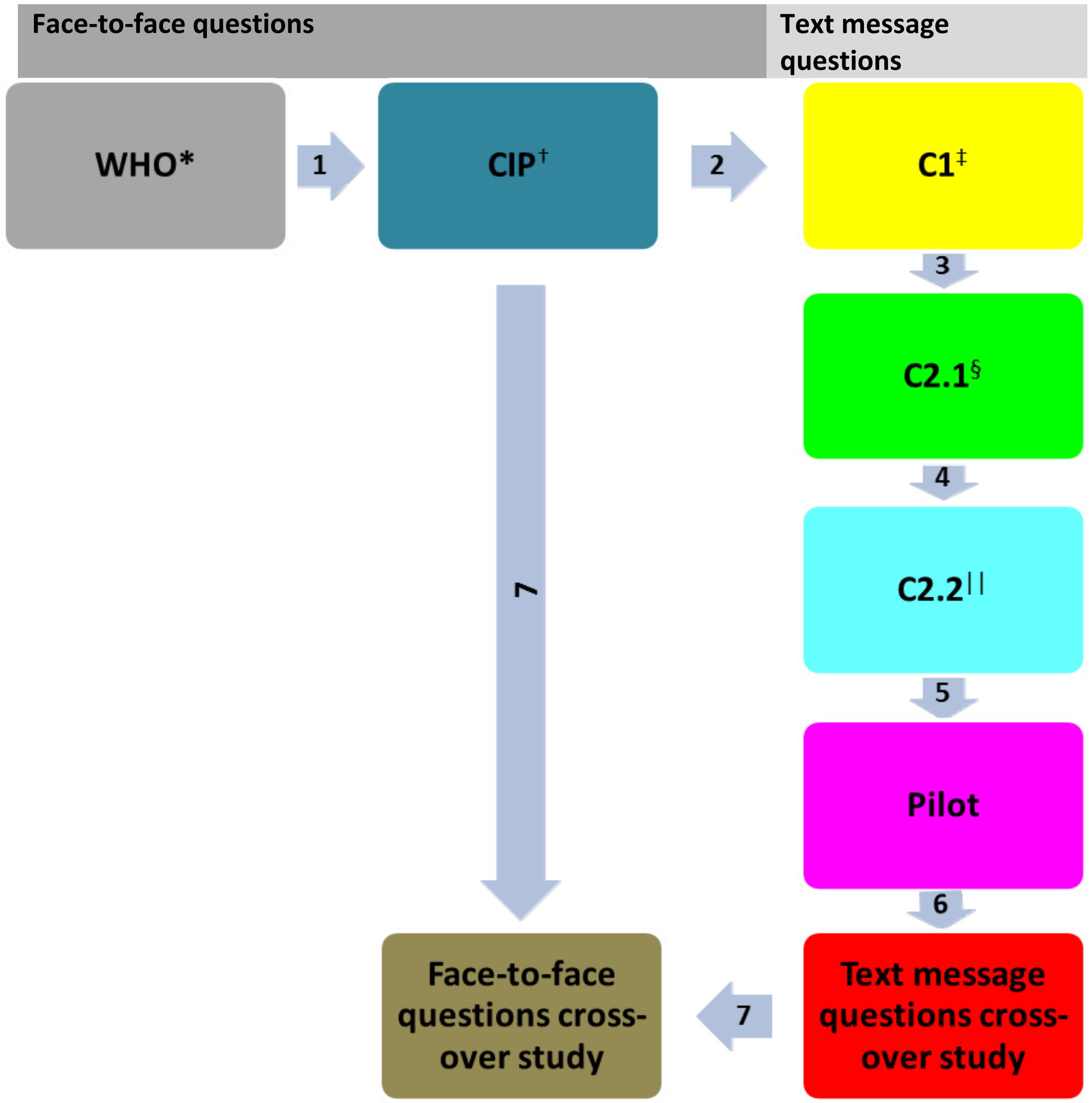

Figure S1 Flow diagram of question development

The superscripts in the figure correspond with the superscripts below:
*World Health Organization (WHO) Maternal, Newborn and Child Health (MNCH) survey draft version from October 2009
†WHO MNCH survey adapted by Capital Institute of Pediatrics (CIP) and used in Zhao County in January 2013
‡Cognitive interview round 1
§Cognitive interview round 2 day 1
||Cognitive interview round 2 day 2

(Source: figure created with Microsoft Word 2010 SmartArt)

Table S1 Overview of questions during development of face-to-face and text message survey

Face-to-face			Text messaging					
Existing WHO MNCH survey	CIP survey	Cross-over study survey	Cognitive interviews 1	Cognitive interviews 2.1	Cognitive interviews 2.2	Pilot	Cross-over study	
**Developed**								
NA	October 2009 – December 2012	January – March 2013	July – December 2012	28 January – 1 March 2013	4 March 2013	6-11 March 2013	13-15 March 2013	
**Used**								
NA	1 – 13 January 2013	16-24 March 2013	21-25 January 2013	4 March 2013	5 March 2013	12 March 2013	17-24 March 2013	
**NA**	**Changes in blue**	**Changes in brown**	**Changes* in yellow**	**Changes* in green**	**Changes* in blue**	**Changes* in pink**	**Changes* in red**	**Nr**
			1.Hello, this is Maternal and Child Health Hospital and the Capital Institute of Pediatrics. We want to ask you questions about your child's health via mobile phone text messages. Your information can help us improving health services. Are you willing to answer those questions? Text us the number of your answer: 1.Yes 2.No	Hello, this is Zhao County's Maternal and Child Health Hospital. We want to ask you questions about your youngest child's health. Your responses to our questions are meaningful to us, it can help us improving health services. [Zhao County's Maternal and Child Health Hospital message]	Hello, this is Zhao County's Maternal and Child Health Hospital. We want to ask you questions about your youngest child's health. Your responses to our questions are meaningful to us, it can help us improving child health. [Zhao County's Maternal and Child Health Hospital message]	Hello, this is Zhao County's Maternal and Child Health Hospital. We want to ask you questions about your youngest child's health. Your responses to our questions are meaningful to us, it can help us improving child health. [Zhao County's Maternal and Child Health Hospital message]	Hello, this is Zhao County's Maternal and Child Health Hospital. We want to ask you questions about your youngest child's health. Your responses to our questions are meaningful to us, it can help us improving child health. [Zhao County's Maternal and Child Health Hospital message]	1

	You do not have to pay extra fees and you will be paid back for replying to messages. In addition, if you answer all the questions, you will receive 5 Yuan extra recharged on your mobile phone credit within 2 weeks. Are you willing to answer questions? Please respond: I am willing/I am not willing [Zhao County's Maternal and Child Health Hospital message]	You do not have to pay extra fees and you will be paid back 1 Jiǎo for replying to messages. In addition, if you answer all the questions, you will receive 5 Yuan extra recharged on your mobile phone credit within 2 weeks. Are you willing to answer questions? Please respond: I am willing/I am not willing [Zhao County's Maternal and Child Health Hospital message]	You do not have to pay extra fees and you will be paid back 1 Jiǎo for replying to messages. In addition, if you answer all the questions, you will receive 5 Yuan extra recharged on your mobile phone credit within 2 weeks. Are you willing to answer questions? Please respond: I am willing/I am not willing [Zhao County's Maternal and Child Health Hospital message]	You do not have to pay extra fees and you will be paid back 1 Jiǎo for replying to messages. In addition, if you answer all the questions, you will receive 5 Yuan extra recharged on your mobile phone credit within 2 weeks. Are you willing to answer questions? Please respond: I am willing/I am not willing [Zhao County's Maternal and Child Health Hospital message]	2

						How does your child call you (the relationship between you and your child)? Please respond: mother/father/grandmother/grandfather/other.....(please specify) [Zhao County's Maternal and Child Health Hospital message]	How does your child call you (the relationship between you and your child)? Please respond: mother/father/grandmother/grandfather/other.....(please specify) [Zhao County's Maternal and Child Health Hospital message]	3
DI.2. Has (*name*) had diarrhoea in the last 2 weeks?<br>1.Yes<br>2.No<br>8.Do not know	DI.1. Has (*name*) had diarrhoea in the last 2 weeks (please think back from two weeks ago till today)?<br>1.Yes<br>2.No<br>8.Do not know	DI.1. Has (*name*)[†] had diarrhoea in the last 2 weeks (please think back from two weeks ago till today, emphasize right date[‡])? Diarrhoea is the passage of 3 or more loose or watery stools, compared to usual, per day.<br>1.Yes<br>2.No<br>8.Do not know	2.Diarrhoea is the passage of 3 or more loose or watery stools per day. Has your child had diarrhoea in the last 2 weeks (day till day)? Text us the number of your answer:<br>1.Yes<br>2.No<br>8.Do not know	Diarrhoea is the passage of 3 or more loose or watery stools per day. Has your youngest child had diarrhoea in the last 2 weeks (*day* till *day*)? Please respond: child had diarrhoea/child didn't have diarrhoea [Zhao County's Maternal and Child Health Hospital message]	Diarrhoea is the passage of 3 or more loose or watery stools per day. Has your youngest child had diarrhoea in the last 2 weeks (*day* till *day*)? Please respond: child had diarrhoea/child didn't have diarrhoea [Zhao County's Maternal and Child Health Hospital message]	Diarrhoea is the passage of 3 or more loose or watery stools per day. Has your youngest child had diarrhoea in the last 2 weeks (*month*/*day* till *day*)? Please respond: child had diarrhoea/child didn't have diarrhoea [Zhao County's Maternal and Child Health Hospital message]	Diarrhoea is the passage of 3 or more loose or watery stools, compared to usual, per day. Has your youngest child[†] had diarrhoea in the last 2 weeks (from *month/day* till *today*[§])? Please respond: child had diarrhoea/child didn't have diarrhoea [Zhao County's Maternal and Child Health Hospital message]	4

DI.3. Did (*name*) have blood in the stools?<br>1.Yes<br>2.No<br>8.Do not know	DI.2. Did (*name*) have blood in the stools?<br>1.Yes<br>2.No<br>8.Do not know	DI.2. Did (*name*)[†] have blood in the stools? *(explain that the blood should be caused by diarrhoea)*<br>1.Yes<br>2.No<br>8.Do not know	3.Did your child have blood in the stools? Text us the number of your answer:<br>1.Yes<br>2.No<br>8.Do not know	Did your youngest child have blood in the stools (caused by diarrhoea)? Please respond: child had blood in stools/child did not have blood in stools [Zhao County's Maternal and Child Health Hospital message]	Did your youngest child have blood in the stools (caused by diarrhoea)? Please respond: child had blood in stools/child did not have blood in stools [Zhao County's Maternal and Child Health Hospital message]	Did your youngest child have blood in the stools (caused by diarrhoea)? Please respond: child had blood in stools/child did not have blood in stools [Zhao County's Maternal and Child Health Hospital message]	Did your youngest child have blood in the stools (caused by diarrhoea)? Please respond: child had blood in stools/child did not have blood in stools [Zhao County's Maternal and Child Health Hospital message]	5

DI.4. During this last episode of diarrhoea, did (*name*) drink any of the following:<br>*Read each item aloud and record response before proceeding to the next.*<br>A fluid made from a packet called [*Local ORS name*]?<br>1. Yes 2. No	DI.3. During this last episode of diarrhoea, did (*name*) drink any of the following:<br>*Read each item aloud and record response before proceeding to the next.*<br>1.A fluid made from a packet called ORS?<br>1.Yes<br>2.No<br>8.Do not know	DI.3. During this last episode of diarrhoea, did (*name*)[†] drink any of the following:<br>*Read each item aloud and record response before proceeding to the next.*<br>1.A fluid made from a packet called ORS?<br>*(explain ORS is drug for diarrhoea treatment)*<br>1.Yes<br>2.No<br>8.Do not know	4.During this last episode of diarrhoea, did your child drink any of the following:<br>1.a fluid made from a packet called ORS<br>2.a recommended homemade fluid such as tap water, mineral water or soup<br>3.other homemade fluids such as tea or drinks. Please text the all the numbers (1, 2, 3) related to the fluid(s) you gave your child.	During this last episode of diarrhoea, did your youngest child drink a fluid made from a packet called ORS (drug which is a mixture of clean water, salt and sugar)? Please respond: child had ORS/child did not have ORS [Zhao County's Maternal and Child Health Hospital message]	During this last episode of diarrhoea, did your youngest child drink a fluid made from a packet called ORS (drug for diarrhoea treatment)? Please respond: child had ORS/child did not have ORS [Zhao County's Maternal and Child Health Hospital message]	During this last episode of diarrhoea, did your youngest child drink a fluid made from a packet called ORS (drug for diarrhoea treatment)? Please respond: child had ORS/child did not have ORS [Zhao County's Maternal and Child Health Hospital message]	During this last episode of diarrhoea, did your youngest child drink a fluid made from a packet called ORS (drug for diarrhoea treatment)? Please respond: child had ORS/child did not have ORS [Zhao County's Maternal and Child Health Hospital message]	6
A recommended homemade fluid [recommended fluid]?<br>1. Yes 2. No	2. A recommended homemade fluid [tap water, mineral water, rice water or soup]?<br>1.Yes<br>2.No<br>8.Do not know	2.One of the following fluids: breast milk, formula, tap water, mineral water, rice water or soup?<br>1.Yes<br>2.No<br>8.Do not know		During this last episode of diarrhoea, did your youngest child drink one of the following fluids: breast milk, formula, tap water, mineral water, rice water or soup?<br>Please respond: child drank one or more of those	During this last episode of diarrhoea, did your youngest child drink one of the following fluids: breast milk, formula, tap water, mineral water, rice water or soup?<br>Please respond: child drank one or more of those	During this last episode of diarrhoea, did your youngest child drink one of the following fluids: breast milk, formula, tap water, mineral water, rice water or soup?<br>Please respond: child drank one or more of those	During this last episode of diarrhoea, did your youngest child drink one of the following fluids: breast milk, formula, tap water, mineral water, rice water or soup?<br>Please respond: child drank one or more of those fluids/child did not drink those fluids	7

				fluids/child did not drink those fluids [Zhao County's Maternal and Child Health Hospital message]	fluids/child did not drink those fluids [Zhao County's Maternal and Child Health Hospital message]	fluids/child did not drink those fluids [Zhao County's Maternal and Child Health Hospital message]	[Zhao County's Maternal and Child Health Hospital message]	
Other homemade fluids [SSS or other not recommended]?<br>1. Yes 2. No	3.Other homemade fluids [tea or drinks]?<br>1.Yes<br>2.No<br>8.Do not know	3.Other fluids such as tea, drinks, water with honey or any sugary drinks?<br>1.Yes<br>2.No<br>8.Do not know		During this last episode of diarrhoea, did your youngest child drink other fluids such as tea and drinks?<br>Please respond: child drunk other fluids/child did not drink other fluids [Zhao County's Maternal and Child Health Hospital message]	During this last episode of diarrhoea, did your youngest child drink other fluids such as tea and drinks?<br>Please respond: child drunk other fluids/child did not drink other fluids [Zhao County's Maternal and Child Health Hospital message]	During this last episode of diarrhoea, did your youngest child drink other fluids such as tea and drinks?<br>Please respond: child drunk other fluids/child did not drink other fluids [Zhao County's Maternal and Child Health Hospital message]	During this last episode of diarrhoea, did your youngest child drink other fluids such as tea, drinks, water with honey or any sugary drinks?<br>Please respond: child drunk other fluids/child did not drink other fluids [Zhao County's Maternal and Child Health Hospital message]	8

DI.5. During (*name's*) illness, did he/she drink much less, about the same, or more than usual? *If less probe*: Was he/she offered much less than usual to drink or somewhat less?<br>1.Much less or none<br>2.About the same or somewhat less<br>3.More<br>8.Do not know	DI.3.a During (*name's*) diarrhoea, did he/she drink much less, about the same, or more than usual? *If less probe*: Was he/she offered much less than usual to drink or somewhat less?<br>1.None<br>2.Much less<br>3.Somewhat less<br>4.About the same<br>5.More<br>8.Do not know	DI.3.a During (*name's*)† diarrhoea, did he/she drink much less, about the same, more than usual or none? *If less probe*: Was he/she offered much less than usual to drink or somewhat less?<br>1.None<br>2.Much less<br>3.Somewhat less<br>4.About the same<br>5.More<br>8.Do not know	5.During your child's diarrhoea, did he/she drink much less, about the same, or more of any liquid than usual? Text us the number of your answer:<br>1.None<br>2.Much less<br>2.Little less<br>3.About the same<br>4.More<br>8.Do not know	During your youngest child's diarrhoea, how much did he/she drink (liquids, breast milk or formula) compared to usual?<br>Please respond: none/much less /somewhat less/about the same/more [Zhao County's Maternal and Child Health Hospital message]	During your youngest child's diarrhoea, how much did he/she drink (liquids, breast milk or formula) compared to usual?<br>Please respond: none/much less /somewhat less/about the same/more [Zhao County's Maternal and Child Health Hospital message]	During your youngest child's diarrhoea, how much did he/she drink (liquids, breast milk or formula) compared to usual?<br>Please respond: none/much less /somewhat less/about the same/more [Zhao County's Maternal and Child Health Hospital message]	During your youngest child's diarrhoea, how much did he/she drink compared to usual (anything the child can drink, including breast milk or formula)?<br>Please respond: none/much less /somewhat less/about the same/more [Zhao County's Maternal and Child Health Hospital message]	9
DI.6. When (*name*) had diarrhea, did he/she eat less, about the same, or more food than usual? *If less probe*: Much less or a little less?<br>1.None<br>2.Much less<br>3.Somewhat less<br>4.About the same<br>5.More	DI.3b. When (*name*) had diarrhoea, did he/she eat less, about the same, or more food than usual? *If less probe*: Much less or a little less?<br>1.Much less or none<br>2.Somewhat less<br>3.About the same<br>4.More	DI.3.b When (*name*)† had diarrhoea, did he/she eat less, about the same, more food than usual or none? *If less probe*: Much less or a little less?<br>1.None<br>2.Much less<br>3.Somewhat less<br>4.About the same	6.When your child had diarrhoea, did he/she eat less, about the same, or more food than usual? Text us the number of your answer:<br>1.Much less or none<br>2.Little less<br>3.About the same<br>4.More<br>5.Child never	When your youngest child had diarrhoea, how much did he/she eat (including solid and semi-solid foods, breast milk and formula) compared to usual?<br>Please respond: none/much less/somewhat	When your youngest child had diarrhoea, how much did he/she eat (including solid and semi-solid foods, breast milk and formula) compared to usual?<br>Please respond: none/much less/somewhat	When your youngest child had diarrhoea, how much did he/she eat (including solid and semi-solid foods, breast milk and formula) compared to usual?<br>Please respond: none/much less/somewhat	Has your youngest child ever been introduced to foods such as rice, noodles, manto, meat, eggs, vegetables, fruits (excluding breast milk or formula)?<br>Please respond: child received foods before/child never received foods before [Zhao	10

6.Child never received solid or semi-solid foods<br>8.Do not know	5.Child never received solid or semi-solid foods<br>8.Do not know	5.More<br>6.Child never received solid or semi-solid foods<br>8.Do not know	received solid or semi-solid foods<br>8.Do not know	less/about the same/more [Zhao County's Maternal and Child Health Hospital message]	less/about the same/more [Zhao County's Maternal and Child Health Hospital message]	less/about the same/more [Zhao County's Maternal and Child Health Hospital message]	County's Maternal and Child Health Hospital message]<br>*For children who have been introduced to complementary foods sent:*<br>When your youngest child had diarrhoea, how much did he/she eat (including all foods, excluding breast milk and formula) compared to usual?<br>Please respond: none/much less/somewhat less/about the same/more [Zhao County's Maternal and Child Health Hospital message]

DI.7. Did you seek advice or treatment for the diarrhoea outside the home?<br>1.Yes<br>2.No<br>8.Do not know	DI.4. Did you seek advice or treatment for the diarrhoea?<br>1.Yes, seek advice or treatment outside home<br>2.No, managed at home<br>8.Do not know	DI.4. During this last episode of diarrhoea in *(name)*[†], did you seek advice or treatment for the diarrhoea outside the home? *(explain that this means asking anyone who is not a family member living with them)*<br>1.Yes, outside the home<br>2.No, managed at home<br>8.Do not know	7.Did you seek advice or treatment for the diarrhoea outside the home? Text us the number of your answer:<br>1.Yes, outside the home<br>2.No, at home<br>8.Do not know	Did you seek advice or treatment when your youngest child had diarrhoea outside the home (ask anyone who is not a family member)? Please respond: yes, outside the home/no, at home [Zhao County's Maternal and Child Health Hospital message]	During this last episode of diarrhoea in your youngest child, did you seek advice or treatment outside the home (ask anyone who is not a family member)? Please respond: yes, outside the home/no, at home [Zhao County's Maternal and Child Health Hospital message]	During this last episode of diarrhoea in your youngest child, did you seek advice or treatment outside the home (ask anyone who is not a family member)? Please respond: yes, outside the home/no, at home [Zhao County's Maternal and Child Health Hospital message]	During this last episode of diarrhoea in your youngest child, did you seek advice or treatment outside the home (ask anyone who is not a family member living with you)? Please respond: yes, outside the home/no, at home [Zhao County's Maternal and Child Health Hospital message]	11
DI.8. Why didn't you seek advice? *Only one answer allowed.*<br>01.Mild disease/did not need outside help<br>02.Geographical access (too far from facility)<br>03.Costs (had to pay for visit or transportation)<br>04.Facility	DI.4.a. Why didn't you seek advice? *Only one answer allowed.*<br>01.Mild disease/did not need outside help<br>02.Geographical access (too far from facility)<br>03.Costs (had to pay for visit or transportation)<br>04.Facility	DI.4.a. During this last episode of diarrhoea in *(name)*[†], why didn't you seek advice? *Only one answer allowed.*<br>01.Mild disease/did not need outside help<br>02.Geographical access (too far from facility)<br>03.Costs (had to	8.Why did you not you seek advice or treatment for the diarrhoea outside the home?	Why did you not seek advice when your youngest child had diarrhoea outside the home? Please respond by giving us one reason, the most important one. [Zhao County's Maternal and Child Health Hospital message]	During this last episode of diarrhoea in your youngest child, why did you not seek advice diarrhoea outside the home? Please respond by giving us one reason, the most important one. [Zhao County's Maternal and	During this last episode of diarrhoea in your youngest child, why did you not seek advice diarrhoea outside the home? Please respond by giving us one reason, the most important one. [Zhao County's Maternal and	During this last episode of diarrhoea in your youngest child, why did you not seek advice diarrhoea outside the home? Please respond by giving us one reason, the most important one. [Zhao County's Maternal and Child Health Hospital	12

closed/staff not available<br>05.Poor quality of care at facility<br>06.Not allowed to go, harmful traditional healer etc.<br>07.Religious beliefs<br>08.Other: specify ________<br>88.Do not know	closed/staff not available<br>05.Poor quality of care at facility<br>06.Not necessary<br>07.Religious beliefs<br>08.Other: specify ________<br>88.Do not know	pay for visit or transportation)<br>04.Facility closed/staff not available<br>05.Poor quality of care at facility<br>06.Not necessary<br>07.Religious beliefs<br>08.Other: specify ________<br>88.Do not know			Child Health Hospital message]	Child Health Hospital message]	message]	
DI.9. Where did you seek care?<br>*Record all sources mentioned.*<br>*Prompt:*<br>*"Anywhere else?"*<br>Public health sector<br>1.Yes 2.No<br>Hospital<br>1.Yes. 2.No<br>Health center/dispensary<br>1.Yes. 2.No<br>Outreach/mobile clinic<br>1.Yes. 2.No<br>Other public<br>1.Yes. 2.No<br>Specify:________	DI.4.b Where did you seek care?<br>*Record all sources mentioned.*<br>*Prompt:*<br>*"Anywhere else?"*<br>1.Relative or friend<br>11.Own family<br>1.Yes 2.No<br>12.Friends or neighbours<br>1.Yes 2.No<br>2.Health facility<br>21.County level hospital or above (excluding MCH hospital)<br>1.Yes 2.No<br>22.County level	DI.4.b. Where did you seek care when (*name*)[†] had diarrhoea?<br>*Record all sources mentioned.*<br>*Prompt:*<br>*"Anywhere else?"*<br>1.Relative or friend<br>11.Own family<br>1.Yes 2.No<br>12.Friends or neighbours<br>1.Yes 2.No<br>2.Health facility<br>21.County level hospital or above (excluding MCH hospital)	9.Where did you seek advice or treatment?	Where did you seek advice or treatment when your youngest child had diarrhoea?<br>Please respond by telling us all the places you went to. [Zhao County's Maternal and Child Health Hospital message]	Where did you seek advice or treatment when your youngest child had diarrhoea?<br>Please respond by telling us all the places you went to. [Zhao County's Maternal and Child Health Hospital message]	Where did you seek advice or treatment when your youngest child had diarrhoea?<br>Please respond by telling us all the places you went to. [Zhao County's Maternal and Child Health Hospital message]<br><br>*After response, prompt:*<br>"Did you go anywhere else during the last episode of	Where did you seek advice or treatment when your youngest child had diarrhoea?<br>Please respond by telling us all the places you went to. [Zhao County's Maternal and Child Health Hospital message]<br><br>*After response, prompt:*<br>"Did you go anywhere else during the last episode of diarrhoea in your	13

Private health sector<br>1.Yes 2.No<br>Hospital<br>1.Yes. 2.No<br>Private dispensary/health center<br>1.Yes 2.No<br>Pharmacy<br>1.Yes 2.No<br>Other private<br>1.Yes. 2.No<br>Specify: ________<br>Community<br>1.Yes 2.No<br>Community health worker<br>1.Yes. 2.No<br>Traditional healer/Traditional birth attendant<br>1.Yes. 2.No<br>Other:<br>1.Yes 2.No<br>Specify: ________	MCH hospital<br>1.Yes 2.No<br>23.Community health centre<br>1.Yes 2.No<br>24.Township hospital<br>1.Yes 2.No<br>25.Community health station<br>1.Yes 2.No<br>26.Village clinic<br>1.Yes 2.No<br>3.Private health facility<br>31.Private hospital<br>1.Yes 2.No<br>32.Private clinic<br>1. Yes. 2.No<br>33.Pharmacy<br>1.Yes 2.No<br>4.Community<br>41.Midwife<br>1.Yes 2.No<br>42.Staff for family planning<br>1.Yes 2.No<br>5.Other: ________<br>1.Yes 2.No<br>Specify: ________	1.Yes 2.No<br>22.County level MCH hospital<br>1.Yes 2.No<br>23.Community health centre<br>1.Yes 2.No<br>24.Township hospital<br>1.Yes 2.No<br>25.Community health station<br>1.Yes 2.No<br>26.Village clinic<br>1.Yes 2.No<br>3.Private health facility<br>31.Private hospital<br>1.Yes 2.No<br>32.Private clinic<br>1. Yes. 2.No<br>33.Pharmacy<br>1.Yes 2.No<br>4.Community<br>41.Midwife<br>1.Yes 2.No<br>42.Staff for family planning<br>1.Yes 2.No<br>5.Other: ________<br>1.Yes 2.No<br>Specify: _______	diarrhoea in your youngest child? Please respond by telling us all the places you want to. [Zhao County's Maternal and Child Health Hospital message]"	youngest child? Please respond by telling us all the places you went to. [Zhao County's Maternal and Child Health Hospital message]"

CO.3. Has (*name*) been ill with a fever at any time in the last 2 weeks, that is, since (DAY OF THE WEEK) of the week before last?<br>1.Yes<br>2.No<br>8.Do not know	CO.1. Has (*name*) been ill with a fever at any time in the last 2 weeks (please think back from two weeks ago till today)?<br>1.Yes<br>2.No<br>8.Do not know	CO.1. Has (*name*)[†] been ill with a fever at any time in the last 2 weeks, (please think back from two weeks ago till today, emphasize right date[‡])?<br>1.Yes<br>2.No<br>8.Do not know	10.Children have fever when they have a high temperature equal to or above 37.5 degrees of Celsius. Has your child had fever at any time in the last 2 weeks (from day till day)? Text us the number of your answer:<br>1.Yes<br>2.No<br>8.Do not know	Has your youngest child had fever at any time in the last 2 weeks (*day* till *day*)?<br>Please respond: child had fever/child did not have fever [Zhao County's Maternal and Child Health Hospital message]	Has your youngest child had fever at any time in the last 2 weeks (*day* till *day*)?<br>Please respond: child had fever/child did not have fever [Zhao County's Maternal and Child Health Hospital message]	Has your youngest child had fever at any time in the last 2 weeks (*month*/*day* till *day*)?<br>Please respond: child had fever/child did not have fever [Zhao County's Maternal and Child Health Hospital message]	Has your youngest child had fever at any time in the last 2 weeks (from *month/day* till *today*[§])?<br>Please respond: child had fever/child did not have fever [Zhao County's Maternal and Child Health Hospital message]	14
CO.4. Has (*name*) had an illness with a cough at any time in the last 2 weeks, that is, since (*day of the week*) of the week before last?<br>1.Yes<br>2.No<br>8.Do not know	CO.2. Has (*name*) had an illness with a cough at any time in the last 2 weeks (please think back from two weeks ago till today)?<br>1.Yes<br>2.No<br>8.Do not know	CO.2. Has (*name*)[†] had an illness with a cough at any time in the last 2 weeks (please think back from two weeks ago till today, emphasize right date[‡])?<br>1.Yes<br>2.No<br>8.Do not know	11.Has your child had cough caused by illness at any time in the last 2 weeks (from day till day)? Text us the number of your answer:<br>1.Yes<br>2.No<br>8.Do not know	Has your youngest child had cough caused by illness at any time in the last 2 weeks (*day* till *day*)?<br>Please respond: child had cough caused by illness/child did not have cough caused by illness [Zhao County's Maternal and Child Health Hospital message]	Has your youngest child had cough caused by illness at any time in the last 2 weeks (*day* till *day*)?<br>Please respond: child had cough caused by illness/child did not have cough caused by illness [Zhao County's Maternal and Child Health Hospital message]	Has your youngest child had cough caused by illness at any time in the last 2 weeks (*month* /*day* till *day*)?<br>Please respond: child had cough caused by illness/child did not have cough caused by illness [Zhao County's Maternal and Child Health Hospital message]	Has your youngest child had cough caused by illness at any time in the last 2 weeks (from *month/day* till *today*[§])?<br>Please respond: child had cough caused by illness/child did not have cough caused by illness [Zhao County's Maternal and Child Health Hospital message]	15

CO.5. When (*name*) had an illness with a cough, did he/she breathe faster than usual with short, fast breaths or have difficulty breathing?<br>1.Yes<br>2.No<br>8.Do not know	CO.2.a When (*name*) had an illness with a cough, did he/she breathe faster than usual with short, fast breaths or have difficulty breathing?<br>1.Yes<br>2.No<br>8.Do not know	CO.2.a Did (*name*)[†] breathe faster than usual with short, fast breaths or have difficulty breathing *(local terms)* in the last 2 weeks (please think back from two weeks ago till today, emphasize right date[‡])?<br>1.Yes<br>2.No<br>8.Do not know	12.Count the number of breaths your child has in one minute. When your child is younger than 1 year, fast breathing is when your child has more than 50 breaths in one minute. When your child is older than 1 year, fast breathing is when your child has more than 40 breaths in one minute.<br>Did your child breathe faster than usual with short, fast breaths or have difficulty breathing in the last 2 weeks (from day till day)? Text us the number of your answer:<br>1.Yes<br>2.No<br>8.Do not know	Did your youngest child breathe faster than usual with short, fast breaths or have difficulty breathing in the last 2 weeks (day till day)?<br>Please respond: child had/child didn't have [Zhao County's Maternal and Child Health Hospital message]	Did your youngest child breathe faster than usual with short, fast breaths or have difficulty breathing in the last 2 weeks (*day* till *day*)?<br>Please respond: child had/child didn't have [Zhao County's Maternal and Child Health Hospital message]	Did your youngest child breathe faster than usual with short, fast breaths or have difficulty breathing in the last 2 weeks (*month*/*day* till *day*)?<br>Please respond: child had/child didn't have [Zhao County's Maternal and Child Health Hospital message]	Did your youngest child breathe faster than usual with short, fast breaths or have difficulty breathing in the last 2 weeks (from *month/day* till *today*[§])?<br>Please respond: child had/child didn't have [Zhao County's Maternal and Child Health Hospital message]	16

CO.6. Were the symptoms due to a problem in the chest or a blocked nose?<br>1.Problem in the chest<br>2.Blocked nose<br>3.Both<br>4.Other, specify: ____<br>8.Do not know	CO.2.b Were the symptoms due to a problem in the chest or a blocked nose?<br>1.Problem in the chest<br>2.Blocked nose<br>3.Both<br>4.Other, specify: ____<br>8.Do not know	CO.2.b Were the symptoms due to a problem in the chest or a blocked nose?<br>1.Problem in the chest<br>2.Blocked nose<br>3.Both<br>4.Other, specify: ____<br>8.Do not know	13.Were the fast breathing or difficult breathing due to a problem in the chest or a blocked nose? Text us the number of your answer:<br>1.Problem in the chest<br>2.Blocked nose<br>3.Both problem in the chest and blocked nose<br>4.Other reason<br>8.Do not know	What's the reason for the fast breathing or difficult breathing? Please respond: problem in the chest/blocked nose/both problem in the chest and blocked nose/other reason……(please give reason) [Zhao County's Maternal and Child Health Hospital message]	What's the reason for the fast breathing or difficult breathing? Please respond: problem in the chest/blocked nose/both problem in the chest and blocked nose/other reason……(please give reason) [Zhao County's Maternal and Child Health Hospital message]	What's the reason for the fast breathing or difficult breathing? Please respond: problem in the chest/blocked nose/both problem in the chest and blocked nose/other reason……(please give reason) [Zhao County's Maternal and Child Health Hospital message]	What's the reason for the fast breathing or difficult breathing? Please respond: problem in the chest/blocked nose/both problem in the chest and blocked nose/other reason……(please give reason) [Zhao County's Maternal and Child Health Hospital message]	17

CO.7 Did you seek advice or treatment for the fever/cough outside the home?<br>1.Yes<br>2.No<br>8.Do not know	CO.4.a Did you seek advice or treatment for the fever/cough?<br>1.Yes, seek advice or treatment outside home<br>2.No, managed at home<br>8.Do not know	CO.4.a During this last episode of fever or cough in (*name*)[†], did you seek advice or treatment for the fever/cough outside the home? *(explain that this means asking anyone who is not a household family member living with them)*<br>1.Yes<br>2.No<br>8.Do not know	14. Did you seek advice or treatment for fever or cough outside the home?<br>Text us the number of your answer:<br>1.Yes<br>2.No<br>8.Do not know	Did you seek advice or treatment for fever or cough outside the home (ask anyone who is not a family member)?<br>Please respond: yes, outside the home/no, at home. [Zhao County's Maternal and Child Health Hospital message]	During this last episode of fever or cough in your youngest child, did you seek advice or treatment outside the home (ask anyone who is not a family member)?<br>Please respond: yes, outside the home/no, at home. [Zhao County's Maternal and Child Health Hospital message]	During this last episode of fever or cough in your youngest child, did you seek advice or treatment outside the home (ask anyone who is not a family member)?<br>Please respond: yes, outside the home/no, at home. [Zhao County's Maternal and Child Health Hospital message]	During this last episode of fever or cough in your youngest child, did you seek advice or treatment outside the home (ask anyone who is not a family member living with you)?<br>Please respond: yes, outside the home/no, at home. [Zhao County's Maternal and Child Health Hospital message]	18

CO.8. Why didn't you seek advice? *Only one answer allowed.*<br>01.Mild disease/did not need outside help<br>02.Geographical access (too far from facility)<br>03.Costs (had to pay for visit or transportation)<br>04.Facility closed/staff not available<br>05.Poor quality of care at facility<br>06.Not allowed to go, harmful traditional healer etc.<br>07.Religious beliefs<br>08.Other: specify<br>_________<br>88.Do not know	CO.4. Why didn't you seek advice? *Only one answer allowed.*<br>01.Mild disease/did not need outside help<br>02.Geographical access (too far from facility)<br>03.Costs (had to pay for visit or transportation)<br>04.Facility closed/staff not available<br>05.Poor quality of care at facility<br>06.Not necessary<br>07.Religious beliefs<br>08.Other: specify<br>_________<br>88.Do not know	CO.4. During this last episode of fever or cough in (*name*)[+], why didn't you seek advice? *Only one answer allowed.*<br>01.Mild disease/did not need outside help<br>02.Geographical access (too far from facility)<br>03.Costs (had to pay for visit or transportation)<br>04.Facility closed/staff not available<br>05.Poor quality of care at facility<br>06.Not necessary<br>07.Religious beliefs<br>08.Other: specify<br>_________<br>88.Do not know	15.Why did you not seek advice for the fever or cough?	Why did you not seek advice for the fever or cough outside the home?<br>Please respond by giving us one reason, the most important one. [Zhao County's Maternal and Child Health Hospital message]	During this last episode of fever or cough in your youngest child, why did you not seek advice outside the home?<br>Please respond by giving us one reason, the most important one.<br>[Zhao County's Maternal and Child Health Hospital message]	During this last episode of fever or cough in your youngest child, why did you not seek advice outside the home?<br>Please respond by giving us one reason, the most important one.<br>[Zhao County's Maternal and Child Health Hospital message]	During this last episode of fever or cough in your youngest child, why did you not seek advice outside the home?<br>Please respond by giving us one reason, the most important one.<br>[Zhao County's Maternal and Child Health Hospital message]	19

CO.09. Where did you seek advice or treatment?<br>*Record all sources mentioned.*<br>*Prompt:*<br>*"Anywhere else?"*<br>Public health sector<br>1.Yes 2.No<br>Hospital<br>1.Yes. 2.No<br>Health center/dispensary<br>1.Yes. 2.No<br>Outreach/mobile clinic<br>1.Yes. 2.No<br>Other public<br>1.Yes. 2.No<br>Specify:_________<br>Private health sector<br>1.Yes 2.No<br>Hospital<br>1.Yes. 2.No<br>Private dispensary/health center<br>1.Yes 2.No<br>Pharmacy<br>1.Yes 2.No<br>Other private	CO.4.a Where did you seek advice or treatment?<br>*Record all sources mentioned.*<br>*Prompt:*<br>*"Anywhere else?"*<br>1.Relative or friend<br>11.Own family<br>1.Yes 2.No<br>12.Friends or neighbours<br>1.Yes 2.No<br>2.Health facility<br>21.County level hospital or above (excluding MCH hospital)<br>1.Yes 2.No<br>22.County level MCH hospital<br>1.Yes 2.No<br>23.Community health centre<br>1.Yes 2.No<br>24.Township hospital<br>1.Yes 2.No<br>25.Community health station<br>1.Yes 2.No<br>26.Village clinic	CO.4.a Where did you seek advice or treatment when (*name*)[†] had fever or cough?<br>*Record all sources mentioned.*<br>*Prompt:*<br>*"Anywhere else?"*<br>1.Relative or friend<br>11.Own family<br>1.Yes 2.No<br>12.Friends or neighbours<br>1.Yes 2.No<br>2.Health facility<br>21.County level hospital or above (excluding MCH hospital)<br>1.Yes 2.No<br>22.County level MCH hospital<br>1.Yes 2.No<br>23.Community health centre<br>1.Yes 2.No<br>24.Township hospital<br>1.Yes 2.No<br>25.Community health station	16.Where did you seek advice or treatment for the fever or cough?	Where did you seek advice or treatment when your youngest child had fever or cough?<br>Please respond by telling us all the places you went to. [Zhao County's Maternal and Child Health Hospital message]	Where did you seek advice or treatment when your youngest child had fever or cough?<br>Please respond by telling us all the places you went to. [Zhao County's Maternal and Child Health Hospital message]	Where did you seek advice or treatment when your youngest child had fever or cough?<br>Please respond by telling us all the places you went to. [Zhao County's Maternal and Child Health Hospital message]<br><br>*After response, prompt:*<br>Did you go anywhere else during the last episode of fever and cough in your youngest child?<br>Please respond by telling us all the places you went to. [Zhao County's Maternal and Child Health Hospital message]	Where did you seek advice or treatment when your youngest child had fever or cough?<br>Please respond by telling us all the places you went to. [Zhao County's Maternal and Child Health Hospital message]<br><br>*After response, prompt:*<br>Did you go anywhere else during the last episode of fever and cough in your youngest child?<br>Please respond by telling us all the places you went to. [Zhao County's Maternal and Child Health Hospital message]	20

1.Yes. 2.No<br>Specify:<br>______________<br>Community<br>1.Yes 2.No<br>Community health worker<br>1.Yes. 2.No<br>Traditional healer/Traditional birth attendant<br>1.Yes. 2.No<br>Other:<br>1.Yes 2.No<br>Specify: ________	1.Yes 2.No<br>3.Private health facility<br>31.Private hospital<br>1.Yes 2.No<br>32.Private clinic<br>1. Yes. 2.No<br>33.Pharmacy<br>1.Yes 2.No<br>4.Community<br>41.Midwife<br>1.Yes 2.No<br>42.Staff for family planning<br>1.Yes 2.No<br>5.Other:<br>1.Yes 2.No<br>Specify: _________	1.Yes 2.No<br>26.Village clinic<br>1.Yes 2.No<br>3.Private health facility<br>31.Private hospital<br>1.Yes 2.No<br>32.Private clinic<br>1. Yes. 2.No<br>33.Pharmacy<br>1.Yes 2.No<br>4.Community<br>41.Midwife<br>1.Yes 2.No<br>42.Staff for family planning<br>1.Yes 2.No<br>5.Other:<br>1.Yes 2.No<br>Specify: __

	This is the end of the survey. Thank you very much for participating! You will receive the reimbursement for your text messages and 5 Yuan for participating automatically on your mobile phone credit within two weeks. [Zhao County's Maternal and Child Health Hospital message]	This is the end of the survey. Thank you very much for participating! You will receive the reimbursement for your text messages and 5 Yuan for participating automatically on your mobile phone credit within two weeks. [Zhao County's Maternal and Child Health Hospital message]	This is the end of the survey. Thank you very much for participating! You will receive 1 Jiǎo per text message and 5 Yuan for participating automatically on your mobile phone credit within two weeks. [Zhao County's Maternal and Child Health Hospital message]	This is the end of the survey. Thank you very much for participating! You will receive 1 Jiǎo per text message and 5 Yuan for participating automatically on your mobile phone credit within two weeks. [Zhao County's Maternal and Child Health Hospital message]	21

*For the text message questions, the questions for each phase (one phase=one column) were the text messages that were used for that phase. The coloured changes were the changes that we made in the previous phase. The reasons for the changes are specified in Table S2-S5 below; the changes were made based on what caregivers said during interviews in the previous phase and on discussions in our multidisciplinary team of experts in child health and mHealth. During the interviews, we paid extra attention to the highlighted changes to ensure that they did not alter the meaning of the original questions and that they improved clarity, so that caregivers could understand the questions.

†For the face-to-face questions, the smartphone programme could insert the name of the child automatically in each question. We wanted to do this in a similar way for the text messaging survey, because it could prevent confusion about which child was meant in the question (when the caregiver had more than one child). In addition, it could have made the text messages more personal. However, this was technically not possible. Therefore, we identified the child in the text messaging survey by the words "your youngest child", because when a caregiver had two children under five, we wanted to ask about the youngest child. Although China has a one-child policy, especially in rural areas there are a significant proportion of families who have more than one child. Therefore, by asking each question about the youngest child, this prevented that caregivers answered the questions for an older child.

‡For the text message questions, after trying different formats for the time, we found that the format (from *month/day* till *today*) was the clearest and shortest in Chinese characters. For the face-to-face questions, this format was technically not possible. Therefore, in the face-to-face survey, we asked for “in the last 2 weeks (please think back from two weeks ago till today, emphasize right date)”. Based on previous fieldwork experiences, this was the clearest way of asking caregivers to recall information.

§For the cross-over study, “today” was changed into “yesterday” in group 1, because the face-to-face survey took place the day before the text messaging survey and we wanted to ask for information in the same recall period.

Table S2 Text messages with changes marked green, based on cognitive interviews round 1 (C1)

These text messages were used for cognitive interviews round 2, day 1 (C2.1)

Nr	Text messages in English and Chinese with marked changes	Changes	Explanation for change based on findings and reasoning
All	See text messages below	See below	We aimed to make text messages that were clear, meaningful and could be trusted, because caregivers indicated in the cognitive interviews that these were important themes for them. Where possible, we kept these findings in mind when making changes in the text messages.
All	See text messages below	Combine information where possible in 1 text message/65 Chinese characters	We decided to combine questions and answer options together in one text message to reduce the number of text messages when possible. We were able to do this for all the questions apart from the first question that asked whether caregivers were willing to participate (see text message 1 below). Caregivers considered this information important for being reminded about the survey information and we could not shorten this information to make it fit into one text message. We asked caregivers about their preference for receiving text messages that contained much information together or separately. During the first round of cognitive interviews, we tested this by randomly sending the text messages together and separately. Caregivers did not have a consistent preference for one or the other. However, one caregiver said to be focused on the first part of the text message and did not read the second part of the text message when it was very long. Also, if we sent text messages that contained more than 65 Chinese characters (one text message), text messages were split. Sometimes it happened that the second part of the text message was delayed and this confused caregivers.
All	See text messages below	Provide additional information to clarify the meaning of text message survey questions, but remove complicated information on disease signs and symptoms.	We decided to add information to clarify the meaning of questions. In the text messaging survey it was not possible to directly clarify the meaning of the question as we did in the face-to-face survey. Therefore, we decided to add information to the some of the questions to clarify the meaning of the question. However, we could not explain everything, because adding too much information on certain topics (such as fever and fast and difficult breathing) confused caregivers.

All	See text messages below	Adding local terms	We added the local terms for diarrhoea, blood in stools, cough, fever and fast and difficult breathing based on the local terminology study (Local terminology study).
All	See text messages below	Provide survey answer options in text messages	We decided to provide caregivers the answer options in the text message survey. In the face-to-face survey, the answer options were not given to caregivers; the interviewer asked the question, listened to the caregiver's response and selected the most appropriate answer. If we did not given caregivers the answer options in the text message survey, they could respond with any answer and it was time-consuming to categorize their answers and to follow-up ambiguous answers.
All	See text messages below	"Return" between question and answer options	We decided to start the answer options on a new line where possible. One caregiver said that it was clearer to read the answer options when they started on a new line.
All	See text messages below "~~Do not know/~~"	Omit answer option "do not know"	We decided to not give caregivers the option "do not know", because this may have encouraged caregivers to chose this option. The answer "do not know" was not desirable as it was a missing value and this may have caused bias. Therefore, we only coded the data as "do not know" when the caregiver wrote this in the text message.
All	See text messages below	Respond by Chinese characters, instead of responding by numbers	We decided to give the answer options in Chinese characters for each question. The first reason for this was that during the cognitive interviews several caregivers ignored our request to respond by numbers. Caregivers said that it was easier to respond by Chinese characters with a Chinese mobile phone, because caregivers had to change the setting in their mobile phone to insert numbers. The second reason was that when using the text messaging system, the responses were not automatically organised. Therefore, it may not have been directly clear to which question a caregiver gave a response if the answers were all the same. By asking caregivers to respond with the answer in Chinese characters, it was clearer for which question they gave an answer. For example for the question "Are you willing to respond to those questions?"' The answer was not just "yes", but "I am willing" or "I am not willing. Thereby, the answer could be distinguished from answers to another question like: "Did your child have diarrhoea?", because answers were: "have diarrhoea" (this means yes) or "do not have" (this means no).*

All	See text messages below	Omit text message number in the text message	We decided to omit the text message number in the text messages. During pilot cognitive interviews, caregivers said that they liked to see which question number they received, because then they knew how many questions they answered. We added numbers in the first cognitive interview round, but then caregivers said that this was sometimes confusing. Adding numbers was also prone to error and time-consuming, because the number of the question depended on the question algorithm and the text message researcher had to insert this manually.
All	See text messages below	Small changes in Chinese characters to fit the information into 65 Chinese characters	In China, a text message can contain up to 65 Chinese characters. Sometimes we had to change some of the Chinese characters to make the information fit in 65 characters. This was done by a native Chinese speaker and checked by a second native Chinese speaker, who were both involved in the study.
All	See the green marking in the text messages below<br>"[Zhao County's Maternal and Child Health Hospital message]<br>【县妇幼短信】"	Add "[Zhao County's Maternal and Child Health Hospital message]"	We decided to add a "label" to each text message that said "Zhao County's Maternal and Child Health Hospital". The text message software company asked us to do this, so that they could ensure that the text messages were successfully sent out. Also, we thought that the label gave the text messages more trustworthiness. Caregivers said that they wanted to know who sent the text messages. They were familiar with Zhao County's Maternal and Child Health Hospital and therefore we hoped that caregivers recognized the label and trusted the text messages.
All	See the green marking in the text messages below<br>"Youngest", "最小的"	Add "youngest" before "child" in all text messages	We decided to add "youngest" before "child" in all text messages, because families could have more than one child younger than five.

1	Hello, this is Zhao County's Maternal and Child Health Hospital. We want to ask you questions about your youngest child's health. Your responses to our questions are meaningful to us, it can help us improving health services. [Zhao County's Maternal and Child Health Hospital message]<br><br>您好，我们是赵县妇幼，想了解您家里最小的那个孩子健康有关情况。您的回复对我们很重要，可以改进卫生保健服务。【县妇幼短信】	Omit "and the Capital Institute of Pediatrics"	We decided to omit that the questions were sent by the Capital Institute of Pediatrics in Beijing, because most caregivers were not familiar with the Capital Institute of Pediatrics in Beijing. The previous sentence said *"Hello, this is Maternal and Child Health Hospital and the Capital Institute of Pediatrics."* While we gave this information to the caregivers, they may have forgotten about this by the time they received the text message.
		Add "Zhao County's"	We decided to add "Zhao County's" before "Maternal and Child Health Hospital" to clarify which hospital we meant. All caregivers were familiar with Zhao County's Maternal and Child Health Hospital.
		Separate introduction (1) and question about willingness to participate (2)	We decided to combine questions and answer options together in one text message to reduce the number of text messages when possible (see changes for all text messages above). We were not able to do this for the first question that asked about whether caregivers were willing to participate. The previous text message (Table S1) said: "*Hello, this is Maternal and Child Health Hospital and the Capital Institute of Pediatrics. We want to ask you questions about your child's health via mobile phone text messages. Your information can help us improving health services. Are you willing to answer those questions? Text us the number of your answer: 1.Yes 2.No*". However, this information was not sufficient and we had to add more information, which could not fit in one text message.
		Omit "mobile phone text messaging"	We decided to change "*We want to ask you questions about your child's health via mobile phone text messages.*", into simply "*We want to ask you questions about your youngest child's health.*", because it was obvious that this was via mobile phone text messaging and these words could be deleted to shorten the text message.
		Add that caregiver's response is meaningful and important	We decided to add that the caregiver's response was meaningful to motive caregivers to respond.

2	You do not have to pay extra fees and you will be paid back for replying to messages. In addition, if you answer all the questions, you will receive 5 Yuan extra recharged on your mobile phone credit within 2 weeks. Are you willing to answer questions?<br>Please respond: I am willing/I am not willing [Zhao County's Maternal and Child Health Hospital message]<br><br>回短信无额外费用，并将得话费补偿。答完所有问题还将得 5 元话费。2 周内充到您手机。您愿意回答吗？<br>请回复：愿意/不愿意【县妇幼短信】	Add information on paying back money for text message fees and receiving an incentive for completing the survey<br><br>Add "within 2 weeks"	We decided to add information about paying back text message fees, because one caregiver worried about the costs of 0.1 Yuan per text message. Also, we decided to give caregivers a small incentive of 5 Yuan for completing the survey; that was similar to the towel given for the face-to-face interview, which was also worth 5 Yuan.<br><br>We added "within 2 weeks", because we thought that it should be clear when the money was given.
3	*Text message 3 was added at a later stage*	NA	NA
4	Diarrhoea is the passage of 3 or more loose or liquid stools per day. Has your youngest child had diarrhoea in the last 2 weeks (from *day* till *day*)?<br>Please respond: child had diarrhoea/child didn't have diarrhoea [Zhao County's Maternal and Child Health Hospital message]<br><br>拉肚子/拉稀指稀便或水样便一天三次或以上。您家最小的孩子过去两周内（*从…到…*）拉肚子/拉稀了吗？<br>请回复：拉了/没拉【县妇幼短信】	Information and question together	See changes "Combine information where possible in 1 text message/65 Chinese characters" above.
5	Did your youngest child have blood in the stools (caused by diarrhoea)?<br>Please respond: child had blood in stools/child did not have blood in stools [Zhao County's Maternal and Child Health Hospital message]<br><br>您家最小的孩子有过因拉肚子/拉稀导致的大便里边带血吗？<br>请回复：带血/不带血【县妇幼短信】	Add information to clarify meaning of question	We decided to add the information "(caused by diarrhea)" to clarify the meaning of the question. For this question the WHO guidelines said that only blood associated with diarrhoea was meant. Other causes of blood in stools, such as constipation, were not meant. We thought about adding "(not including blood caused by constipation)", but as this may have confused caregivers, and therefore we did not use this phrase.

6	During this last episode of diarrhoea, did your youngest child drink a fluid made from a packet called ORS (drug which is a mixture of clean water, salt and sugar)?<br>Please respond: child had ORS/child did not have ORS [Zhao County's Maternal and Child Health Hospital message]<br><br>您最小的孩子最近一次拉肚子/拉稀期间，喝了口服补液盐吗(糖、盐与水混合的药)？<br>请回复：喝了口服补液盐/没喝口服补液盐【县妇幼短信】	Separate question in three text messages	We decided to separate the question about drinking during diarrhoea into three questions, as specified in the WHO guidelines. We considered combining these three questions in one text message to reduce the number of text messages that had to be sent. However, caregivers did not understand the questions correct during the cognitive interviews. We realized that the meaning of the question changed by combining the three questions in one text message. Therefore, we asked the three questions in three separate text messages.
		Clarify the meaning of ORS	We decided to clarify the meaning of ORS, because caregivers said that they did not knew what ORS was.
7	During this last episode of diarrhoea, did your youngest child drink one of the following fluids: breast milk, formula, tap water, mineral water, rice water or soup?<br>Please respond: child drank one or more of those fluids/child did not drink those fluids [Zhao County's Maternal and Child Health Hospital message]<br><br>您最小孩子最近一次拉肚子/拉稀期间，是否喝了以下任一种液体：母乳、配方奶粉、白开水、矿泉、水、米汤、菜汤？<br>请回：喝过这些/都没喝过【县妇幼短信】	New text message	See text message 6.
		Omit "recommended"	We decided to omit the word "recommended" to not confuse caregivers. Caregivers asked why it said "recommended". One caregiver did not knew which drinks were recommended, but thought that this meant that other homemade fluids such as tea and drinks were less good, because they were not recommended.
		Add breast milk and formula	We completed the list of locally recommended fluids by adding breast milk and formula.

8	During this last episode of diarrhoea, did your youngest child drink other fluids such as tea and drinks?<br>Please respond: child drunk other fluids/child did not drink other fluids [Zhao County's Maternal and Child Health Hospital message]<br><br>您家最小的孩子最近一次拉肚子/拉稀期间，是否喝了其他液体，如茶水或饮料等？<br>请回复：喝过其他液体/没喝其他液体【县妇幼短信】	New text message	See text message 6.
		Omit "homemade"	We decided to omit the word "homemade", because this was not necessary for understanding the question and omitting it reduced the number of Chinese characters in the text message.
9	During your youngest child's diarrhoea, how much did he/she drink (liquids, breast milk or formula) compared to usual?<br>Please respond: none/much less/somewhat less/about the same/more [Zhao County's Maternal and Child Health Hospital message]<br><br>您最小孩子那次拉肚子/拉稀期间，喝的液体量（汤水、母乳和配方奶）比平常怎样？<br>请回：一点没喝/少得多/少些/一样/多些【县妇幼短信】	Rephrase question	We decided to rephrase this question; we moved information in the question to the answer options. Caregivers said the previous question was unclear, because the answer options (less, about the same, or more) were in the question: *"During your child's diarrhoea, did he/she drink much less, about the same, or more of any liquid than usual?"*. We omitted the answer options in the question and replaced this by "how much compared to usual".
		Add "liquids, breast milk, or formula" between brackets	We decided to add the information "liquids, breast milk or formula" between brackets to clarify the meaning of the question. The WHO guidelines specified that drinking included all liquids.
10	When your youngest child had diarrhoea, how much did he/she eat (including solid and semi-solid foods, excluding breast milk and formula) compared to usual?<br>Please respond: none/much less/somewhat less/about the same/more [Zhao County's Maternal and Child Health Hospital message]<br><br>您最小孩子那次拉肚子/拉稀期间，吃固体、半固体、母乳和配方奶的量比平常怎样？<br>请回：没吃/少很多/少些/一样/多些/？【县妇幼短信】	Rephrase question	We decided to rephrase this question; we moved information in the question to the answer options. Caregivers said that the previous question was unclear, because the answer options (less, about the same, or more) were in the question: *"During your child's diarrhoea, did he/she eat much less, about the same, or more of any liquid than usual?"*. We omitted the answer options in the question and replaced this by "how much compared to usual".
		Separate the answer options "none" and "much less"	We decided to split the answer options "none" and "much less" to be consistent with the WHO guidelines.
		Remove the answer option "never received solid or semi-solid foods"	We decided to remove the answer option "never received solid or semi-solid foods", because this could be confused with "none".
		Add information to clarify question meaning	We decided to add "(including solid and semi-solid foods, breast milk and formula)", which was specified in the WHO guidelines.

11	Did you seek advice or treatment when your youngest child had diarrhoea outside the home (ask anyone who is not a family member)?<br>Please respond: yes, outside the home/no, at home [Zhao County's Maternal and Child Health Hospital message]<br><br>您最小孩子拉肚子/拉稀时，向家里以外的人寻求过指导或治疗了吗(问家里人不算)?<br>请回复：寻求过 /没有寻求 【县妇幼短信】	Add information to clarify question meaning	We decided to add extra information between brackets, because caregivers often only thought about going to a doctor for seeking advice or treatment (this question meant to ask about seeking advice to anyone outside the family, also neighbours or friends).
12	Why did you not seek advice when your youngest child had diarrhoea outside the home?<br>Please respond by giving us one reason, the most important one [Zhao County's Maternal and Child Health Hospital message]<br><br>您最小孩子拉肚子/拉稀时您为什么没有到家庭外寻求指导或治疗?<br>请回复一个最主要原因【县妇幼短信】	Add "Please respond by giving us one reason, the most important one"	We decided to add "please respond by giving us one reason, the most important one", because caregivers may otherwise have given many reasons for why they did not seek advice outside the home. The WHO guidelines specified to record only the most important reason. In Chinese we had to add "one" to make this clear.
13	Where did you seek advice or treatment when your youngest child had diarrhoea?<br>Please respond by telling us all the places you went to [Zhao County's Maternal and Child Health Hospital message]<br><br>您最小孩子拉肚子/拉稀时你到哪里寻求指导和治疗？<br>请回复去的所有地方。【县妇幼短信】	Add "Please respond by telling us all the places you went to"	We decided to add "please respond by telling us all the places you went to", because caregivers may only have given one answer and the WHO guidelines specified to list all the places caregivers went too.
14	Has your youngest child had fever at any time in the last 2 weeks (from *day* till *day*)?<br>Please respond: child had fever/child did not have fever [Zhao County's Maternal and Child Health Hospital message]<br><br>您最小孩子在过去 2 周内 （*从...到...*）有过发烧吗？<br>请回复：发烧了/没发烧【县妇幼短信】	Delete information	We decided to omit the information on fever, because this was confusing for caregivers. All caregivers knew what fever was, but they found the information about the temperature degree confusing. Caregivers said that they did not measure the body temperature of their child. Some had incorrect ideas about what the temperature of fever was. One caregiver said that it should be when the temperature of the child was higher than 36.5 degrees (normal temperature according to this caregiver).

15	Has your youngest child had cough caused by illness at any time in the last 2 weeks (from *day* till *day*)?<br>Please respond: child had cough caused by illness/child did not have cough caused by illness [Zhao County’s Maternal and Child Health Hospital message]<br><br>您最小孩子在过去 2 周内 *（从...到...）* 有过因为生病引起咳嗽吗？<br>请回复：有过生病引起的咳嗽/没有过生病引起的咳嗽【县妇幼短信】	No change	One caregiver responded that the child had cough because of “上火 Shàng huǒ”, which is a term from Traditional Chinese Medicine. While most Chinese people know this term, there is no clear consensus what it means. There is no equivalent term for “上火 Shàng huǒ” in Western Medicine, but a translation could be “being under the weather”. We decided not to change anything, because if a caregiver mentioned that cough was caused by “上火 Shàng huǒ”, this was likely to be less serious than a common cough.
16	Did your youngest child breathe faster than usual with short, fast breaths or have difficulty breathing in the last 2 weeks (from *day* till *day*)?<br>Please respond: child had/ child didn’t have [Zhao County’s Maternal and Child Health Hospital message]<br><br>您最小孩子 2 周内有过呼吸比平时快而短，或者有喘不上气/憋得慌吗？（从...到...）<br>请回复：有过/没有【县妇幼短信】	Omit information about fast and difficult breathing	We decided to omit the information about fast and difficult breathing, because caregivers found the information on counting breaths confusing. Fast and difficult breathing were difficult concepts for caregivers and no one counted breaths or paid attention to it. Some caregivers said that they were willing to count breaths, but others did not want to do this.
		Change terms for fast breathing and difficult breathing	We added the local terms for fast and difficult breathing. One caregiver thought difficult and fast breathing were the same. This finding was confirmed in the local terminology study. However, this did not bias the answer to this question, because the question did not differentiate between fast and difficult breathing.
		Change answer options	We decided to change the answer options, because in Chinese it was not clear to say “fast or difficult breathing”. The answer options had to be specified as “child had/didn't have”.
17	What’s the reason for the fast breathing or difficult breathing? Please respond: problem in the chest/blocked nose/both problem in the chest and blocked nose/other reason……(please give reason) [Zhao County’s Maternal and Child Health Hospital message]<br><br>您的孩子呼吸快或喘不上气/憋得慌是因为什么？<br>请回复： 肺部问题/鼻腔堵塞/两者都有/其他原因（请列出）【县妇幼短信】	Rephrase question	We decided to rephrase this question; we moved information in the question to the answer options. Caregivers said the previous question was unclear, because the answer options (a problem in the chest or a blocked nose) were in the question: *“Were the fast breathing or difficult breathing due to a problem in the chest or a blocked nose?”*. Therefore, we thought that it was better to omit the answer options in the question. This also made the question shorter.

18	Did you seek advice or treatment for fever or cough outside the home (ask anyone who is not a family member)?<br>Please respond: yes, outside the home/no, at home [Zhao County's Maternal and Child Health Hospital message]<br><br>孩子发烧/咳嗽时，你为孩子寻求指导或治疗吗？<br>请回复：向家里以外的人寻求指导或治疗了吗？请回复：寻求过/没有寻求【县妇幼短信】	Add information to clarify question meaning	We decided to add extra information between brackets, because caregivers often only thought about going to a doctor for seeking advice or treatment (this question meant to ask about seeking advice to anyone outside the family, also neighbours or friends).
19	Why did you not seek advice for the fever or cough outside the home?<br>Please respond by giving us one reason, the most important one [Zhao County's Maternal and Child Health Hospital message]<br><br>孩子发烧/咳嗽时您为什么没有到家庭外寻求指导或治疗?<br>请回复一个最主要原因【县妇幼短信】	Add "outside the home"	We decided to add "outside the home", because this was clearer and consistent with text message question 12.
		Add "Please respond by giving us one reason, the most important one"	We decided to add "please respond by giving us one reason, the most important one", because caregivers may otherwise have given many reasons for why they did not seek advice outside the home. The WHO guidelines specified only to record the most important reason. In the Chinese language we had to add "one" to make this clear.
20	Where did you seek advice or treatment when your youngest child had fever or cough?<br>Please respond by telling us all the places you went to [Zhao County's Maternal and Child Health Hospital message]<br><br>孩子发烧/咳嗽时到哪里寻求指导或治疗？<br>请回复列出去的所有地方。 【县妇幼短信】	Add "Please respond by telling us all the places you went to"	We decided to add "please respond by telling us all the places you went to", because caregivers may only have given one answer and the WHO guidelines specified to list all the places caregivers went too.

21	This is the end of the survey. Thank you very much for participating! You will receive the reimbursement for your text messages and 5 Yuan for participating automatically on your mobile phone credit within two weeks. [Zhao County's Maternal and Child Health Hospital message]<br><br>调查结束，非常感谢您的参与！您将在两周内得到短信费及 5 元话费的补偿，将直接充到您的手机上。【县妇幼短信】	Add "end question"	We decided to add a final text messages that indicated the end of the survey.

*In Chinese, there is no fixed "yes" or "no"; the answer depends on the question asked. When saying "yes", people will confirm what is asked. When saying "no", this can either be "no" (bù) or "don't have" (méi yǒu).

Table S3 Text messages with changes marked blue, based on cognitive interviews round 2 day 1 (C2.1)

These text messages were used for cognitive interviews round 2, day 2 (C2.2)

Nr	Text messages in English and Chinese with marked changes	Changes	Explanation for change based on findings and reasoning
1	Hello, this is Zhao County's Maternal and Child Health Hospital. We want to ask you questions about your youngest child's health. Your responses to our questions are meaningful to us, it can help us improving child health. [Zhao County's Maternal and Child Health Hospital message]<br><br>您好，我们是赵县妇幼，想了解您家里最小的那个孩子健康有关情况。您的回复对我们很重要，可以帮助我们改善儿童健康状况。【县妇幼短信】	Change "health services" into "child health"	We decided to change "health services" into "child health", because one caregiver said that she could not understand the words "health services". She suggested us to use "child health".
2	You do not have to pay extra fees and you will be paid back 1 Jiǎo for replying to messages. In addition, if you answer all the questions, you will receive 5 Yuan extra recharged on your mobile phone credit within 2 weeks. Are you willing to answer questions?<br>Please respond: I am willing/I am not willing [Zhao County's Maternal and Child Health Hospital message]<br><br>回短信无额外费用，并会返还短信费 1 角/条。答完所有问题可另得 5 元话费。2 周内充到您手机。您愿意回答吗？<br>请回复：愿意/不愿意【县妇幼短信】	Add "1 Jiǎo"	We decided to add 1 Jiǎo (0.1 Yuan) to the sentence about paying back money for the text message fees, because caregivers thought they only received 5 Yuan.
3	*Text message 3 was added at a later stage*	NA	NA

4	Diarrhoea is the passage of 3 or more loose or liquid stools per day. Has your youngest child had diarrhoea in the last 2 weeks (day till day)?<br>Please respond: child had diarrhoea/child didn’t have diarrhoea [Zhao County’s Maternal and Child Health Hospital message]<br><br>拉肚子（拉稀）指稀便或水样便一天三次或以上。您家最小孩子过去两周内 *（…到…）* 拉肚子/拉稀了吗？<br>请回：拉了/没拉【县妇幼短信】		
5	Did your youngest child have blood in the stools (caused by diarrhoea)?<br>Please respond: child had blood in stools/child did not have blood in stools [Zhao County’s Maternal and Child Health Hospital message]<br><br>您家最小的孩子有过因拉肚子/拉稀导致的大便里边带血吗？<br>请回复：带血/不带血【县妇幼短信】		
6	During this last episode of diarrhoea, did your youngest child drink a fluid made from a packet called ORS (drug for diarrhoea treatment)?<br>Please respond: child had ORS/child did not have ORS [Zhao County’s Maternal and Child Health Hospital message]<br><br>您最小孩子最近一次拉肚子/拉稀期间，喝了口服补液盐吗？(一种治疗拉稀的药)请回复：喝了口服补液盐/没喝口服补液盐【县妇幼短信】	Change “drug which is a mixture of clean water, salt and sugar” into “drug for diarrhoea treatment”	We decided to change the explanation from “drug which is a mixture of clean water, salt and sugar” into “drug for diarrhoea treatment”, because caregivers did not understand the first explanation. Not all caregivers had heard about ORS; most caregivers who knew about ORS had a previous experience with treating their child’s diarrhoea with ORS. The caregivers who knew about ORS suggested us to use “drug for diarrhoea treatment”.

7	During this last episode of diarrhoea, did your youngest child drink one of the following fluids: breast milk, formula, tap water, mineral water, rice water or soup?<br>Please respond: child drank one or more of those fluids/child did not drink those fluids [Zhao County's Maternal and Child Health Hospital message]<br><br>您最小孩子最近拉肚子/拉稀期间，是否喝了以下液体：母乳、配方奶、白开水、矿泉水、米汤、菜汤？<br>请回：喝过这些/都没喝过【县妇幼短信】	
8	During this last episode of diarrhoea, did your youngest child drink other fluids such as tea and drinks?<br>Please respond: child drunk other fluids/child did not drink other fluids [Zhao County's Maternal and Child Health Hospital message]<br><br>您家最小的孩子最近一次拉肚子/拉稀期间，是否喝了其他液体，如茶或饮料等？<br>请回复：喝过其他液体/没喝其他液体【县妇幼短信】	
9	During your youngest child's diarrhoea, how much did he/she drink (liquids, breast milk or formula) compared to usual?<br>Please respond: None/ Much less /Somewhat less/About the same/More [Zhao County's Maternal and Child Health Hospital message]<br><br>您最小孩子那次拉肚子/拉稀期间，喝的液体量（汤水、母乳和配方奶）比平常怎样？<br>请回：一点没喝/少得多/少些/一样/多些【县妇幼短信】	

10	When your youngest child had diarrhoea, how much did he/she eat (including solid and semi-solid foods, breast milk and formula) compared to usual? Please respond: none/much less/somewhat less/about the same/more [Zhao County's Maternal and Child Health Hospital message]<br><br>您最小孩子那次拉肚子/拉稀期间，吃固体、半固体、母乳和配方奶的量比平常怎样？<br>请回：没吃/少很多/少些/一样/多些？【县妇幼短信】		
11	During this last episode of diarrhoea in your youngest child, did you seek advice or treatment outside the home (ask anyone who is not a family member)?<br>Please respond: yes, outside the home/no, at home [Zhao County's Maternal and Child Health Hospital message]<br><br>您最小孩子最近这次拉肚子/拉稀时，向家里以外的人寻求过指导或治疗了吗？<br>请回复：寻求过/没有寻求【县妇幼短信】	Add "during this last episode of diarrhoea in your youngest child"	We decided to add "during this last episode of diarrhoea in your youngest child", because caregivers were unclear about which occasion we asked about. They thought that we asked for seeking care in general for any disease and not specifically for this episode of diarrhoea in their child.
12	During this last episode of diarrhoea in your youngest child, why did you not seek advice diarrhoea outside the home? Please respond by giving us one reason, the most important one. [Zhao County's Maternal and Child Health Hospital message]<br><br>您最小孩子最近这次拉肚子/拉稀时，您为什么没有向家里以外的人寻求指导或治疗?<br>请回复一个最主要原因【县妇幼短信】	Add "during this last episode of diarrhoea in your youngest child"	See text message 11.
13	During this last episode of diarrhoea in your youngest child, where did you seek advice or treatment?<br>Please respond by telling us all the places you went to. [Zhao County's Maternal and Child Health Hospital message]<br><br>您最小孩子最近那次拉肚子/拉稀时，您到哪里寻求指导或治疗？请回复去的所有地方。【县妇幼短信】	Add "during this last episode of diarrhoea in your youngest child"	See text message 11.

14	Has your youngest child had fever at any time in the last 2 weeks (day till day)?<br>Please respond: child had fever/child did not have fever. [Zhao County's Maternal and Child Health Hospital message]<br><br>您最小孩子在过去 2 周内 *（从…到…）*有过发烧吗？<br>请回复：发烧了/没发烧【县妇幼短信】
15	Has your youngest child had cough caused by illness at any time in the last 2 weeks (day till day)?<br>Please respond: child had cough caused by illness/child did not have cough caused by illness [Zhao County's Maternal and Child Health Hospital message]<br><br>您最小孩子在过去 2 周内 *（从…到…）*有过因为生病引起咳嗽吗？<br>请回复：有过生病引起的咳嗽/没有过生病引起的咳嗽【县妇幼短信】
16	Did your youngest child breathe faster than usual with short, fast breaths or have difficulty breathing in the last 2 weeks (day till day)?<br>Please respond: child had / child didn't have [Zhao County's Maternal and Child Health Hospital message]<br><br>您最小孩子 2 周内（从…到…）有过呼吸比平时快而短，或者有喘不上气/憋得慌吗？<br>请回复：有过/没有【县妇幼短信】

17	What's the reason for the fast breathing or difficult breathing?<br>Please respond: problem in the chest/blocked nose/both problem in the chest and blocked nose/other reason……(please give reason) [Zhao County's Maternal and Child Health Hospital message]<br><br>您的孩子呼吸快或喘不上气/憋得慌是因为什么？<br>请回复： 肺部问题/鼻腔堵塞/两者都有/其他原因（请列出）【县妇幼短信】		
18	During this last episode of fever or cough in your youngest child, did you seek advice or treatment outside the home (ask anyone who is not a family member)?<br>Please respond: yes, outside the home/no, at home [Zhao County's Maternal and Child Health Hospital message]<br><br>您最小孩子最近那次发烧/咳嗽时，向家里以外的人寻求指导或治疗了吗？<br>请回复：寻求过/没有寻求【县妇幼短信】	Add "during this last episode of fever or cough in your youngest child"	We decided to add "during this last episode of fever or cough in your youngest child", because caregivers were unclear about which occasion we asked about. They thought that we asked for seeking care in general for any disease and not specifically for this episode of fever or cough in their child.
19	During this last episode of fever or cough in your youngest child, why did you not seek advice for the fever or cough outside the home?<br>Please respond by giving us one reason, the most important one [Zhao County's Maternal and Child Health Hospital message]<br><br>您最小孩子最近那次发烧/咳嗽时您为什么没有向家里以外的人寻求指导或治疗?<br>请回复一个最主要原因【县妇幼短信】	Add "during this last episode of fever or cough in your youngest child"	See text message 18.

20	During this last episode of fever or cough in your youngest child, where did you seek advice or treatment when your youngest child had fever or cough?<br>Please respond by telling us all the places you went to. [Zhao County's Maternal and Child Health Hospital message]<br><br>您最小孩子最近那次发烧/咳嗽时您到哪里寻求指导或治疗?<br>请回复列出去的所有地方。【县妇幼短信】	Add "during this last episode of fever or cough in your youngest child"	See text message 18.
21	This is the end of the survey. Thank you very much for participating! You will receive the reimbursement for your text messages and 5 Yuan for participating automatically on your mobile phone credit within two weeks. [Zhao County's Maternal and Child Health Hospital message]<br><br>调查结束，非常感谢您的参与！您将在 2 星期内得到返还的短信费及 5 元话费的补偿，将直接充到您的手机上。【县妇幼短信】		

Table S4 Text messages with changes marked pink, based on cognitive interviews round 2 day 2 (C2.2)

These text messages were used for the pilot

Nr	Text messages in English and Chinese with marked changes	Changes	Explanation for change based on findings and reasoning
1	Hello, this is Zhao County's Maternal and Child Health Hospital. We want to ask you questions about your youngest child's health. Your responses to our questions are meaningful to us, it can help us improving child health. [Zhao County's Maternal and Child Health Hospital message]<br><br>您好，我们是赵县妇幼，想了解您家里最小的那个孩子健康有关情况。您的回复对我们很重要，可以帮助我们改善儿童健康状况。【县妇幼短信】		
2	You do not have to pay extra fees and you will be paid back 1 Jiǎo for replying to messages. In addition, if you answer all the questions, you will receive 5 Yuan extra recharged on your mobile phone credit within 2 weeks. Are you willing to answer questions? Please respond: I am willing/I am not willing [Zhao County's Maternal and Child Health Hospital message]<br><br>回短信无额外费用，并会返还短信费 1 角/条。答完所有问题可另得 5 元话费。2 周内充到您手机。您愿意回答吗？<br>请回复：愿意/不愿意【县妇幼短信】		
3	How does your child call you (the relationship between you and your child)?<br>Please respond: mother/father/grandmother/grandfather/other.....(please specify) [Zhao County's Maternal and Child Health Hospital message]<br><br>您的孩子管您叫什么（您与孩子的关系）？请回复：妈妈/爸爸/奶奶/爷爷/其他（请列出）【县妇幼短信】	New text message	We decided to add this question to confirm whether the caregiver was the same caregiver as the caregiver who participated in the face-to-face interview.

4	Diarrhoea is the passage of 3 or more loose or watery stools per day. Has your youngest child had diarrhoea in the last 2 weeks (*month*/*day* till *day*)?<br>Please respond: child had diarrhoea/child didn't have diarrhoea [Zhao County's Maternal and Child Health Hospital message]<br><br>拉肚子（拉稀）指稀便或水样便一天三次或以上。您家最小孩子过去两周内 *（…月…日到…日）*拉稀了吗？<br>请回：拉了/没拉【县妇幼短信】	Add "month"	We decided to add the month to all the questions in which we asked about the last two weeks. Earlier we decided not to add the month to save space, because it seemed clear to caregivers. However, one caregiver was unclear about the month and therefore we added the month.<br>In the cross-over study, the two weeks fell in one month (March) and we only had to add one month. However, in future studies, the two weeks may fall in separate months and then the text messages may have to be adapted so that two months can fit in the text message.
5	Did your youngest child have blood in the stools (caused by diarrhoea)? Please respond: child had blood in stools/child did not have blood in stools [Zhao County's Maternal and Child Health Hospital message]<br><br>您家最小的孩子有过因拉肚子/拉稀导致的大便里边带血吗？<br>请回复：带血/不带血【县妇幼短信】		
6	During this last episode of diarrhoea, did your youngest child drink a fluid made from a packet called ORS (drug for diarrhoea treatment)? Please respond: child had ORS/child did not have ORS [Zhao County's Maternal and Child Health Hospital message]<br><br>您最小孩子最近一次拉肚子/拉稀期间，喝了口服补液盐吗？(一种治疗拉稀的药)请回复：喝了口服补液盐/没喝口服补液盐【县妇幼短信】		
7	During this last episode of diarrhoea, did your youngest child drink one of the following fluids: breast milk, formula, tap water, mineral water, rice water or soup?<br>Please respond: child drank one or more of those fluids/child did not drink those fluids [Zhao County's Maternal and Child Health Hospital message]<br><br>您最小孩子最近拉肚子/拉稀期间，是否喝了以下液体：母乳、配方奶、白开水、矿泉水、米汤、菜汤？<br>请回：喝过这些/都没喝过【县妇幼短信】		

8	During this last episode of diarrhoea, did your youngest child drink other fluids such as tea and drinks?<br>Please respond: child drunk other fluids/child did not drink other fluids [Zhao County's Maternal and Child Health Hospital message]<br><br>您家最小的孩子最近一次拉肚子/拉稀期间，是否喝了其他液体，如茶或饮料等？<br>请回复：喝过其他液体/没喝其他液体【县妇幼短信】
9	During your youngest child's diarrhoea, how much did he/she drink (liquids, breast milk or formula) compared to usual? Please respond: none/much less /somewhat less/about the same/more [Zhao County's Maternal and Child Health Hospital message]<br><br>您最小孩子那次拉肚子/拉稀期间，喝的液体量（汤水、母乳和配方奶）比平常怎样？<br>请回：一点没喝/少得多/少些/一样/多些【县妇幼短信】
10	When your youngest child had diarrhoea, how much did he/she eat (including solid and semi-solid foods, breast milk and formula) compared to usual? Please respond: none/much less/somewhat less/about the same/more [Zhao County's Maternal and Child Health Hospital message]<br><br>您最小孩子那次拉肚子/拉稀期间，吃固体、半固体、母乳和配方奶的量比平常怎样？<br>请回：没吃/少很多/少些/一样/多些？【县妇幼短信】
11	During this last episode of diarrhoea in your youngest child, did you seek advice or treatment outside the home (ask anyone who is not a family member)? Please respond: Yes, outside the home/No, at home [Zhao County's Maternal and Child Health Hospital message]<br><br>您最小孩子最近这次拉肚子/拉稀时，向家里以外的人寻求过指导或治疗了吗？<br>请回复：寻求过/没有寻求【县妇幼短信】
12	During this last episode of diarrhoea in your youngest child, why did you not seek advice diarrhoea outside the home? Please respond by giving us one reason, the most important one. [Zhao County's Maternal and Child Health Hospital message]<br><br>您最小孩子最近这次拉肚子/拉稀时，您为什么没有向家里以外的人寻求指导或治疗?<br>请回复一个最主要原因【县妇幼短信】

13	During this last episode of diarrhoea in your youngest child, where did you seek advice or treatment? Please respond by telling us all the places you went to. [Zhao County's Maternal and Child Health Hospital message]<br><br>*After response, prompt:*<br>Did you go anywhere else during the last episode of diarrhoea in your youngest child?<br>Please respond by telling us all the places you want to. [Zhao County's Maternal and Child Health Hospital message]<br><br>您最小孩子最近那次拉肚子/拉稀时您到哪里寻求指导或治疗？请回复所有去的地方。【县妇幼短信】<br><br>最小孩子最近那次拉肚子/拉稀时，您还去过其他地方吗？请回复所有去的地方。【县妇幼短信】	Adding a new text message	We decided to add an additional text message asking about other places where caregivers sought care. Earlier we thought not to prompt on this, because we already asked them to list all places they went to. However, caregivers did not give all places they went to.
14	Has your youngest child had fever at any time in the last 2 weeks (*month*/*day* till *day*)?<br>Please respond: Child had fever/Child did not have fever [Zhao County's Maternal and Child Health Hospital message]<br><br>您最小孩子在过去 2 周内（*…月…日到…日*）有过发烧吗？<br>请回复：发烧了/没发烧【县妇幼短信】	Add "month"	See text message 4.
15	Has your youngest child had cough caused by illness at any time in the last 2 weeks (*month*/*day* till *day*)?<br>Please respond: Child had cough caused by illness/Child did not have cough caused by illness [Zhao County's Maternal and Child Health Hospital message]<br><br>您最小孩子在过去 2 周内（*…月…日到…日*）有过因为生病引起咳嗽吗？<br>请回复：有过生病引起的咳嗽/没有过生病引起的咳嗽【县妇幼短信】	Add "month"	See text message 4.
16	Did your youngest child breathe faster than usual with short, fast breaths or have difficulty breathing in the last 2 weeks (*month*/*day* till *day*)?<br>Please respond: child had/child didn't have [Zhao County's Maternal and Child Health Hospital message]<br><br>您最小孩子 2 周内（*…月…日到…日*）有过呼吸比平时快而短，或者有喘不上气/憋得慌吗？<br>请回复：有过/没有【县妇幼短信】	Add "month"	See text message 4.

17	What's the reason for the fast breathing or difficult breathing?<br>Please respond: problem in the chest/blocked nose/both problem in the chest and blocked nose/other reason……(please give reason) [Zhao County's Maternal and Child Health Hospital message]<br><br>您的孩子呼吸快或喘不上气/憋得慌是因为什么？<br>请回复： 肺部问题/鼻腔堵塞/两者都有/其他原因（请列出）【县妇幼短信】
18	During this last episode of fever or cough in your youngest child, did you seek advice or treatment outside the home (ask anyone who is not a family member)?<br>Please respond: yes, outside the home/no, at home [Zhao County's Maternal and Child Health Hospital message]<br><br>您最小孩子最近那次发烧/咳嗽时， 向家里以外的人寻求指导或治疗了吗？<br>请回复：寻求过/没有寻求【县妇幼短信】
19	During this last episode of fever or cough in your youngest child, why did you not seek advice for the fever or cough outside the home?<br>Please respond by giving us one reason, the most important one. [Zhao County's Maternal and Child Health Hospital message]<br><br>您最小孩子最近那次发烧/咳嗽时您为什么没有向家里以外的人寻求指导或治疗?<br>请回复一个最主要原因【县妇幼短信】

20	During this last episode of fever or cough in your youngest child, where did you seek advice or treatment? Please respond by telling us all the places you went to. [Zhao County's Maternal and Child Health Hospital message]<br><br>*After response, prompt:*<br>Did you go anywhere else during the last episode of fever and cough in your youngest child? Please respond by telling us all the places you went to. [Zhao County's Maternal and Child Health Hospital message]<br><br>您最小孩子最近那次发烧/咳嗽时您到哪里寻求指导或治疗？<br>请回复列出所有去的地方。【县妇幼短信】<br><br>最小孩子最近那次发烧/咳嗽时，您还去过其他地方吗？<br>请回复列出所有去的地方。【县妇幼短信】	Adding a new text message	See text message 13.
21	This is the end of the survey. Thank you very much for participating! You will receive 1 Jiǎo per text message and 5 Yuan for participating automatically on your mobile phone credit within two weeks. [Zhao County's Maternal and Child Health Hospital message]<br><br>调查结束，非常感谢您的参与！您将在 2 星期内得到返还的短信费 1 角/条及 5 元话费的补偿，将直接充到您的手机上。【县妇幼短信】	Add "1 Jiǎo per text message"	We decided to add how much caregivers were paid back for sending their text messages, because it was unclear to caregivers how much money was given.

Table S5 Text messages with changes marked red, based on pilot

Text messages were used for cross-over study

Nr	Text messages in English and Chinese with marked changes	Changes	Explanation for change based on findings and reasoning
1	Hello, this is Zhao County's Maternal and Child Health Hospital. We want to ask you questions about your youngest child's health. Your responses to our questions are meaningful to us, it can help us improving child health. [Zhao County's Maternal and Child Health Hospital message]<br><br>您好，我们是赵县妇幼，想了解您家里最小的那个孩子健康有关情况。您的回复对我们很重要，可以帮助我们改善儿童健康状况。【县妇幼短信】		
2	You do not have to pay extra fees and you will be paid back 1 Jiǎo for replying to messages. In addition, if you answer all the questions, you will receive 5 Yuan extra recharged on your mobile phone credit within 2 weeks. Are you willing to answer questions?<br>Please respond: I am willing/I am not willing [Zhao County's Maternal and Child Health Hospital message]<br><br>回短信无额外费用，并会返还短信费 1 角/条。答完所有问题可另得 5 元话费。2 周内充到您手机。您愿意回答吗？<br>请回复：愿意/不愿意【县妇幼短信】		
3	How does your child call you (the relationship between you and your child)? Please respond: mother/father/grandmother/grandfather/other…..(please specify) [Zhao County's Maternal and Child Health Hospital message]<br><br>您的孩子管您叫什么（您与孩子的关系）？请回复：妈妈/爸爸/奶奶/爷爷/其他（请列出）【县妇幼短信】		
4	Diarrhoea is the passage of 3 or more loose or liquid stools, compared to usual, per day. Has your youngest child had diarrhoea in the last 2 weeks (from *month/day* till today)? Please respond: child had diarrhoea/child didn't have diarrhoea [Zhao County's Maternal and Child Health Hospital message].<br><br>拉肚子（拉稀）指比平常稀的大便或水样便一天三次或以上。您家最小孩子过去两周内（*…月…日到…*今天）拉肚子/拉稀了吗？<br>请回：拉了/没拉【县妇幼短信】	Add "compared to usual"	We decided to add "compared to usual", because for caregivers with young infants it could be difficult to distinguish loose or watery stools from diarrhoea as stools in young infants are usually loose or watery.
		Change date into "from month/day till today"	We decided to change the data format, because this was the shortest in Chinese and clear to caregivers.

5	Did your youngest child have blood in the stools (caused by diarrhoea)?<br>Please respond: child had blood in stools/child did not have blood in stools [Zhao County's Maternal and Child Health Hospital message]<br><br>您家最小的孩子有过因拉肚子/拉稀导致的大 便带血（拉粑粑里边带血）吗？<br>请回复：带血/不带血【县妇幼短信】		
6	During this last episode of diarrhoea, did your youngest child drink a fluid made from a packet called ORS (drug for diarrhoea treatment)?<br>Please respond: child had ORS/child did not have ORS [Zhao County's Maternal and Child Health Hospital message]<br><br>您最小孩子最近一次拉肚子/拉稀期间，喝了口服补液盐吗？(一种治疗拉稀的药)请回复：喝了口服补液盐/没喝口服补液盐【县妇幼短信】		
7	During this last episode of diarrhoea, did your youngest child drink one of the following fluids: breast milk, formula, tap water, mineral water, rice water or soups?<br>Please respond: child drank one or more of those fluids/child did not drink those fluids [Zhao County's Maternal and Child Health Hospital message]<br><br>您最小孩子最近拉肚子/拉稀期间，是否喝了以下液体：母乳、配方奶、白开水、矿泉水、米汤、菜汤？<br>请回：喝过这些/都没喝过【县妇幼短信】		
8	During this last episode of diarrhoea, did your youngest child drink other fluids such as tea, drinks, water with honey or any sugary drinks?<br>Please respond: child drunk other fluids/child did not drink other fluids [Zhao County's Maternal and Child Health Hospital message]<br><br>您家最小的孩子最近一次拉肚子/拉稀期间，是否喝了其他液体，如茶、饮料、蜂蜜水或任何甜水等？<br>请回复：喝过其他液体/没喝其他液体【县妇幼短信】	Add "water with honey or any sugary drinks"	We decided to add "water with honey or any sugary drinks", because these liquids were commonly given to children in Zhao County.

9	During your youngest child’s diarrhoea, how much did he/she drink (anything the child can drink, including breast milk or formula) compared to usual?<br>Please respond: none/much less /somewhat less/about the same/more [Zhao County’s Maternal and Child Health Hospital message]<br><br>您最小孩子那次拉肚子期间，喝的（任何能喝的，包括母乳和配方奶）比平常怎样？<br>请回：一点没喝/少得多/少些/一样/多些【县妇幼短信】	Add “anything the child can drink, including”	We decided to add “anything the child can drink, including”, because caregivers said that they did not understand what liquids were.
10	Has your youngest child ever been introduced to foods such as rice, noodles, manto, meat, eggs, vegetables, fruits (excluding breast milk or formula)?<br>Please respond: child received foods before/child never received foods before [Zhao County’s Maternal and Child Health Hospital message]<br><br>*For children who received complementary foods before sent:*<br>When your youngest child had diarrhoea, how much did he/she eat (including all foods, excluding breast milk and formula) compared to usual? Please respond: none/much less/somewhat less/about the same/more [Zhao County’s Maternal and Child Health Hospital message]<br><br>您最小的孩子吃过米饭、面条、馒头、肉、蛋、蔬菜或水果这样的物件吗？（除母乳及配方奶）请回：吃过物件/从没吃过物件【县妇幼短信】<br><br>对已经添加了辅食的孩子发送：<br>您最小孩子那次拉肚子期间，吃物件（母乳和配方奶不算）的量比平常怎样？<br>请回：没吃/少很多/少些/一样/多些？【县妇幼短信】	Add pre-selection question on whether child has been introduced to complementary food	We decided to add a pre-selection question on whether the child had been introduced to complementary food. The question about how much the child at did not include the answer option “child was not introduced to complementary food”. We thought that caregivers with children who had not been introduced to complementary food could then potentially either fill in “none” or “child was not introduced to complementary food”, because they may not have been clear about which answer option was appropriate. However, without the option “child was not introduced to complementary food”, it was unclear which children had been introduced to complementary food and which children had not. This information was needed to calculate the corresponding indicator. Therefore, by adding this pre-selection question we could select children who had been introduced to complementary food from the children who had not.
		Add “excluding breast milk and formula”	We decided to clarify that we did not mean to ask about breastfeeding and formula by eating. We added a pre-selection question before this question (see comment above), which filtered out children who had not been introduced to complimentary food. Therefore, this question was only asked to caregivers of children who received complementary food. For these children, the previous question about drinking during diarrhoea already asked about breastfeeding and formula.

11	During this last episode of diarrhoea in your youngest child, did you seek advice or treatment outside the home (ask anyone who is not a family member living with you)?<br>Please respond: yes, outside the home/no, at home [Zhao County's Maternal and Child Health Hospital message]<br><br>您最小孩子最近这次拉肚子/拉稀时，除了住在一起的家人，您还寻求过指导或治疗了吗？<br>请回复：寻求过/没有寻求【县妇幼短信】	Add "living with you".	We decided to add "living with you", because the question meant to ask about people outside the household.
12	During this last episode of diarrhoea in your youngest child, why did you not seek advice diarrhoea outside the home? Please respond by giving us one reason, the most important one [Zhao County's Maternal and Child Health Hospital message]<br><br>您最小孩子最近这次拉肚子/拉稀时，除了住在一起的家人，您为什么没有寻求指导或治疗?<br>请回复一个最主要原因【县妇幼短信】		
13	During this last episode of diarrhoea in your youngest child, where did you seek advice or treatment?<br>Please respond by telling us all the places you went to. [Zhao County's Maternal and Child Health Hospital message]<br><br>*After response, prompt:*<br>Did you go anywhere else during the last episode of diarrhoea in your youngest child? Please respond by telling us all the places you went to. [Zhao County's Maternal and Child Health Hospital message]<br><br>您最小孩子最近那次拉肚子/拉稀时您到哪里寻求指导或治疗？请回复去的所有地方。【县妇幼短信】<br><br>回复后，追问：<br>最小孩子最近那次拉肚子/拉稀时，您还去过其他地方吗？请回复列出所有去的地方。【县妇幼短信】		

14	Has your youngest child had fever at any time in the last 2 weeks (from *month/day* till *today*)?<br>Please respond: child had fever/child did not have fever [Zhao County's Maternal and Child Health Hospital message]<br><br>您最小孩子在过去 2 周内 *（…月…日到…今天）*有过发烧吗？<br>请回复：发烧了/没发烧【县妇幼短信】	Change date into "from month/day till today"	See text message 4.
15	Has your youngest child had cough caused by illness at any time in the last 2 weeks (from *month/day* till *today*)?<br>Please respond: child had cough caused by illness/child did not have cough caused by illness. [Zhao County's Maternal and Child Health Hospital message]<br><br>您最小孩子在过去 2 周内 *（…月…日到…今天）*有过因为生病引起咳嗽吗？<br>请回复：有过生病引起的咳嗽/没有过生病引起的咳嗽【县妇幼短信】	Change date into "from month/day till today"	See text message 4.
16	Did your youngest child breathe faster than usual with short, fast breaths or have difficulty breathing in the last 2 weeks (from *month/day* till *today*)?<br>Please respond: child had /child didn't have [Zhao County's Maternal and Child Health Hospital message]<br><br>您最小孩子 2 周内 *（…月…日到…今天）*有过呼吸比平时快而短，或者有喘不上气/憋得慌吗？<br>请回复：有过/没有【县妇幼短信】	Change date into "from month/day till today"	See text message 4.
17	What's the reason for the fast breathing or difficult breathing?<br>Please respond: problem in the chest/blocked nose/both problem in the chest and blocked nose/other reason……(please give reason) [Zhao County's Maternal and Child Health Hospital message]<br><br>您的孩子呼吸快或喘不上气/憋得慌是因为什么？<br>请回复： 肺部问题/鼻腔堵塞/两者都有/其他原因（请列出）【县妇幼短信】		

18	During this last episode of fever or cough in your youngest child, did you seek advice or treatment outside the home (ask anyone who is not a family member living with you)?<br>Please respond: yes, outside the home/no, at home [Zhao County's Maternal and Child Health Hospital message]<br><br>您最小孩子最近那次发烧/咳嗽时，除了住在一起的家人，您还寻求指导或治疗了吗？<br>请回复：寻求过/没有寻求【县妇幼短信】	Add "living with you"	See text message 11.
19	During this last episode of fever or cough in your youngest child, why did you not seek advice for the fever or cough outside the home?<br>Please respond by giving us one reason, the most important one [Zhao County's Maternal and Child Health Hospital message]<br><br>您最小孩子最近那次发烧/咳嗽时除了住在一起的家人，您为什么没有寻求指导或治疗?<br>请回复一个最主要原因【县妇幼短信】		
20	During this last episode of fever or cough in your youngest child, where did you seek advice or treatment?<br>Please respond by telling us all the places you went to. [Zhao County's Maternal and Child Health Hospital message]<br><br>*After response, prompt:*<br>Did you go anywhere else during the last episode of fever and cough in your youngest child? Please respond by telling us all the places you went to. [Zhao County's Maternal and Child Health Hospital message]<br><br>您最小孩子最近那次发烧/咳嗽时您到哪里寻求指导或治疗？<br>请回复列出所有去的地方。【县妇幼短信】<br><br>回复后，追问：<br>最小孩子最近那次发烧/咳嗽时，您还去过其他地方吗？请回复列出所有去的地方。【县妇幼短信】		

21	This is the end of the survey. Thank you very much for participating! You will receive 1 Jiǎo per text message and 5 Yuan for participating automatically on your mobile phone credit within two weeks. [Zhao County's Maternal and Child Health Hospital message]<br><br>调查结束，非常感谢您的参与！您将在 2 星期内得到返还的短信费及 5 元话费的补偿，将直接充到您的手机上。【县妇幼短信】

Table S6 Face-to-face and text message questions used in cross-over study

Module	Face-to-face questions selected from the World Health Organization (WHO) Maternal Newborn and Child Health (MNCH) survey, diarrhoea and cough and fever modules	Text messages with questions (adapted from face-to-face questions)		
	Question number and content in English and Chinese	Text message number and content in English and Chinese		
Intro	NA	1	Hello, this is Zhao County's Maternal and Child Health Hospital. We want to ask you questions about your youngest child's health. Your responses to our questions are meaningful to us, it can help us improving child health. [Zhao County's Maternal and Child Health Hospital message]	您好，我们是赵县妇幼，想了解您家里最小的那个孩子健康有关情况。您的回复对我们很重要，可以帮助我们改善儿童健康状况。【县妇幼短信】
	NA	2	You do not have to pay extra fees and you will be paid back 1 Jiǎo for replying to messages. In addition, if you answer all the questions, you will receive 5 Yuan extra recharged on your mobile phone credit within 2 weeks. Are you willing to answer questions?<br>Please respond: I am willing/I am not willing [Zhao County's Maternal and Child Health Hospital message]	回短信无额外费用，并会返还短信费 1 角/条。答完所有问题可另得 5 元话费。2 周内充到您手机。您愿意回答吗？<br>请回复：愿意/不愿意【县妇幼短信】
	NA	3	How does your child call you (the relationship between you and your child)?<br>Please respond: mother/father/grandmother/grandfather/other.....(please specify) [Zhao County's Maternal and Child Health Hospital message]	您的孩子管您叫什么（您与孩子的关系）？<br>请回复：妈妈/爸爸/奶奶/爷爷/其他（请列出）【县妇幼短信】

Diarrhoea	DI.1.	Has (*name)* had diarrhoea in the last 2 weeks (please think back from two weeks ago till today? Diarrhoea is the passage of 3 or more loose or watery stools, compared to usual, per day.<br>1.Yes<br>2.No →*Skip to* CO.1<br>8.Do not know→*Skip to* CO.1	孩子过去两星期内*（从今天算起往前推 2 星期，并强调日期）*腹泻（拉稀/拉肚子）了吗？<br>腹泻定义为一天稀便(和平常相比)或水样便三次或以上。<br>1.是<br>2.否 *——>结束 DI 部分，转到 CO.1*<br>8.不知道 *——>结束 DI 部分，转到 CO.1*	4	Diarrhoea is the passage of 3 or more loose or watery stools, compared to usual, per day. Has your youngest child had diarrhoea in the last 2 weeks (from *month/day* till *today*)?<br>Please respond: child had diarrhoea/child didn't have diarrhoea [Zhao County's Maternal and Child Health Hospital message]	拉肚子（拉稀）指比平常稀的大便或水样便一天三次或以上。您家最小孩子过去两周内 *(…月…日到…今天）*拉肚子/拉稀了吗？<br>请回：拉了/没拉【县妇幼短信】
	DI.2.	Did (*name*) have blood in the stools?<br>1.Yes<br>2.No<br>8.Do not know	孩子大便中带血吗？(由于腹泻导致的大便带血)<br>1.是<br>2.否<br>8.不知道	5	Did your youngest child have blood in the stools (caused by diarrhoea)?<br>Please respond: child had blood in stools/child did not have blood in stools [Zhao County's Maternal and Child Health Hospital message]	您家最小的孩子有过因拉肚子/拉稀导致的大便带血（拉粑粑里边带血）吗？<br>请回复：带血/不带血【县妇幼短信】
	DI.3.	During this last episode of diarrhoea, did (*name*) drink any of the following: *read each item aloud and record response before proceeding to the next.*<br>A fluid made from a packet called ORS?<br>1.Yes<br>2.No<br>8.Do not know	孩子在最近一次腹泻期间，你是否给了以下液体：*（逐项读出，并记录每一个选项及答案。）*<br>1.口服补液盐（一种治疗拉稀的药物）<br>1.是<br>2.否<br>8.不知道	6	During this last episode of diarrhoea, did your youngest child drink a fluid made from a packet called ORS (drug for diarrhoea treatment)?<br>Please respond: child had ORS/child did not have ORS [Zhao County's Maternal and Child Health Hospital message]	您最小孩子最近一次拉肚子/拉稀期间，喝了口服补液盐吗？(一种治疗拉稀的药)请回复：喝了口服补液盐/没喝口服补液盐【县妇幼短信】
		One of the following fluids: breast milk, formula, tap water, mineral water, rice water or soup?<br>1.Yes<br>2.No<br>8.Do not know	2.母乳、配方奶、白开水、矿泉水、米汤、菜汤<br>1.是<br>2.否<br>8.不知道	7	During this last episode of diarrhoea, did your youngest child drink one of the following fluids: breast milk, formula, tap water, mineral water, rice water or soup?<br>Please respond: child drank one or more of those fluids/child did not drink those fluids [Zhao County's Maternal and Child Health Hospital message]	您最小孩子最近拉肚子/拉稀期间，是否喝了以下液体：母乳、配方奶、白开水、矿泉水、米汤、菜汤？<br>请回：喝过这些/都没喝过【县妇幼短信】

		Other fluids such as tea, drinks, water with honey or any sugary drinks?<br>1.Yes<br>2.No<br>8.Do not know	3. 其他液体，如茶或饮料、蜂蜜、甜水等<br>1.是<br>2.否<br>8.不知道	8	During this last episode of diarrhoea, did your youngest child drink other fluids such as tea, drinks, water with honey or any sugary drinks?<br>Please respond: child drunk other fluids/child did not drink other fluids [Zhao County's Maternal and Child Health Hospital message]	您家最小的孩子最近一次拉肚子/拉稀期间，是否喝了其他液体，如茶、饮料、蜂蜜水或任何甜水等？<br>请回复：喝过其他液体/没喝其他液体【县妇幼短信】
	DI.3.a	During (*name's*) diarrhoea, did he/she drink much less, about the same, more than usual or none? *If less, probe*: Was he/she offered much less than usual to drink or somewhat less?<br>1.None<br>2.Much less<br>3.Somewhat less<br>4.About the same<br>5.More<br>8.Do not know	孩子最近一次腹泻时，孩子喝的汤水和母乳是比平常少、与平常一样多、比平常多还是什么都没喝？（包括任何孩子能喝的东西，如母乳和配方奶）[如果比平常少，继续问：比平常少很多，还是少一点？]<br>1.什么也没喝<br>2.少得多<br>3.少一点<br>4.一样多<br>5.比平常多<br>8.不知道	9	During your youngest child's diarrhoea, how much did he/she drink compared to usual (anything the child can drink, including breast milk or formula)?<br>Please respond: none/much less /somewhat less/about the same/more [Zhao County's Maternal and Child Health Hospital message]	您最小孩子那次拉肚子期间，喝的（任何能喝的，包括母乳和配方奶）比平常怎样？<br>请回：一点没喝/少得多/少些/一样/多些【县妇幼短信】

	DI.3.b	When (*name*) had diarrhoea, did he/she eat less, about the same, more food than usual or none?<br>*If less, probe*: Much less or a little less?<br>1.None<br>2.Much less<br>3.Somewhat less<br>4.About the same<br>5.More<br>6.Child never received solid or semi-solid foods<br>8.Do not know	孩子最近一次腹泻期间，吃的物件是比平常少、与平常一样多、比平常多还是什么都没吃？[如果比平常少，继续问：比平常少很多，还是少一点？如果没有吃，继续问：是从没吃过还是仅腹泻期间没有吃]<br>1.什么也没吃<br>2.少很多<br>3.少一点<br>4.一样多<br>5.比平常多<br>6.孩子还没有吃过任何固体或半固体的食物<br>8.不知道	10<br><br><br><br><br><br><br><br><br><br><br>10a	Has your youngest child ever been introduced to foods such as rice, noodles, manto, meat, eggs, vegetables, fruits (excluding breast milk or formula)?<br>Please respond: child received foods before/child never received foods before [Zhao County's Maternal and Child Health Hospital message]<br><br><br>*For children who have been introduced to complementary foods sent:*<br>When your youngest child had diarrhoea, how much did he/she eat (including all foods, excluding breast milk and formula) compared to usual?<br>Please respond: none/much less/somewhat less/about the same/more [Zhao County's Maternal and Child Health Hospital message]	您最小的孩子吃过米饭、面条、馒头、肉、蛋、蔬菜或水果这样的物件吗？（除母乳及配方奶）请回：吃过物件/从没吃过物件【县妇幼短信】<br><br><br><br>对已经添加了辅食的孩子发送：<br>您最小孩子那次拉肚子期间，吃物件（母乳和配方奶不算）的量比平常怎样？<br>请回：没吃/少很多/少些/一样/多些？【县妇幼短信】

	DI.4	During this last episode of diarrhoea in (*name*), did you seek advice or treatment for the diarrhoea outside the home<br>1.Yes, outside the home→*skip to* DI.4.b<br>2.No, managed at home<br>8.Do not know	孩子最近这次腹泻期间，除了住在一起的家人，您还寻求过指导或治疗吗？<br>1.是，到家庭外寻求指导或治疗 ——>*转到 DI.4.b*<br>（包括询问邻居、去医院、为孩子举行宗教仪式等。如医生到家中给予指导或治疗，也算在内。当母亲外出寻求指导或治疗时，是否带孩子一起去没有关系，如去药店买药没带孩子，也算在内；不要读出）<br>2. 否，在家里自行处理——>*转到 DI.4a*<br>8. 不知道 ——>*结束本部分，转到下一部分*	11	During this last episode of diarrhoea in your youngest child, did you seek advice or treatment outside the home (ask anyone who is not a family member living with you)?<br>Please respond: yes, outside the home/no, at home [Zhao County's Maternal and Child Health Hospital message]	您最小孩子最近这次拉肚子/拉稀时，除了住在一起的家人，您还寻求过指导或治疗了吗？<br>请回复：寻求过/没有寻求【县妇幼短信】
	DI.4.a	During this last episode of diarrhoea in (*name*), why didn't you seek advice? *Only one answer allowed.*<br>01.Mild disease/did not need outside help<br>02.Geographical access (too far from facility)<br>03.Costs (had to pay for visit or transportation)<br>04.Facility closed/staff not available<br>05.Poor quality of care at facility<br>06.Not necessary<br>07.Religious beliefs<br>08.Other: specify ________<br>88.Do not know	孩子最近这次腹泻期间，没有寻求指导或治疗的主要原因？——> *答完此题，结束本部分*<br>01.不严重/不需要别人帮助<br>02.家离卫生机构太远<br>03.费用（没钱支付服务或交通费）<br>04.卫生机构没有人/没有开门<br>05.卫生机构的服务质量差<br>06.没必要<br>07.宗教信仰<br>08.其他：_______<br>88.不知道	12	During this last episode of diarrhoea in your youngest child, why did you not seek advice diarrhoea outside the home?<br>Please respond by giving us one reason, the most important one. [Zhao County's Maternal and Child Health Hospital message]	您最小孩子最近这次拉肚子/拉稀时，除了住在一起的家人，您为什么没有寻求指导或治疗?<br>请回复一个最主要原因【县妇幼短信】

	DI.4.b	Where did you seek care when (*name*) had diarrhoea?<br>*Record all sources mentioned. Prompt: "Anywhere else?"*<br>1.Relative or friend<br>11.Own family<br>1.Yes 2.No<br>12.Friends or neighbours<br>1.Yes 2.No<br>2.Health facility<br>21.County level hospital or above (excluding MCH hospital)<br>1.Yes 2.No<br>22.County level MCH hospital<br>1.Yes 2.No<br>23.Community health centre<br>1.Yes 2.No<br>24.Township hospital<br>1.Yes 2.No<br>25.Community health station<br>1.Yes 2.No<br>26.Village clinic<br>1.Yes 2.No<br>3.Private health facility<br>31.Private hospital<br>1.Yes 2.No<br>32.Private clinic<br>1. Yes. 2.No<br>33.Pharmacy<br>1.Yes 2.No<br>4.Community<br>41.Midwife<br>1.Yes 2.No<br>42.Staff for family planning<br>1.Yes 2.No<br>5.Other: 1.Yes 2.No Specify: __	孩子最近这次腹泻期间，你到哪里寻求指导和治疗？（不要念出选项，记录所有的提到的地方。可提示"还有其他地方吗？"）<br>1.家中亲友<br>11 自己的家人<br>1.是 2.否<br>12 朋友或邻居<br>1.是 2.否<br>2.公立医疗机构<br>21.县级及以上医院（不含妇幼保健院）<br>1.是 2.否<br>22 县级及以上妇幼保健院<br>1.是 2.否<br>23.社区卫生中心<br>1.是 2.否<br>24.乡镇卫生院<br>1.是 2.否<br>25.社区卫生站<br>1.是 2.否<br>26.村卫生室<br>1.是 2.否<br>3.私营医疗机构<br>31.私营医院<br>1.是 2.否<br>32.个体诊所<br>1.是 2.否<br>33.药店/药贩<br>1.是 2.否<br>4．社区<br>41.接生员<br>1.是 2.否<br>42.计生干事<br>1.是 2.否 5.其他：1.是 2.否____	13<br><br><br><br><br><br><br><br><br>13a	During this last episode of diarrhoea in your youngest child, where did you seek advice or treatment when your youngest child had diarrhoea? Please respond by telling us all the places you went to. [Zhao County's Maternal and Child Health Hospital message]<br><br>*After response, prompt:*<br>Did you go anywhere else during the last episode of diarrhoea in your youngest child? Please respond by telling us all the places you went to. [Zhao County's Maternal and Child Health Hospital message]	您最小孩子最近那次拉肚子/拉稀时您到哪里寻求指导或治疗？请回复去的所有地方。【县妇幼短信】<br><br>回复后，追问：<br>最小孩子最近那次拉肚子/拉稀时，您还去过其他地方吗？请回复列出所有去的地方。【县妇幼短信】

Cough and fever	CO.1.	Has (*name*) been ill with a fever at any time in the last 2 weeks, (please think back from two weeks ago till today)?<br>1.Yes<br>2.No<br>8.Do not know	您最小孩子在过去 2 星期内*（从今天算起往前推 2 星期，并强调日期）*发过烧吗？<br>1.是<br>2.否<br>8.不知道	14	Has your youngest child had fever at any time in the last 2 weeks (from *month/day* till *today*)?<br>Please respond: child had fever/child did not have fever [Zhao County's Maternal and Child Health Hospital message]	您最小孩子在过去 2 周内 *（...月... 日到...今天）*有过发烧吗？<br>请回复：发烧了/没发烧【县妇幼短信】
	CO.2.	Has (*name*) had an illness with a cough at any time in the last 2 weeks (please think back from two weeks ago till today)?<br>1.Yes<br>2.No<br>8.Do not know	您最小孩子在过去 2 星期内*（从今天算起往前推 2 星期，并强调日期）*生过病，且生病时有过咳嗽吗？<br>1.是<br>2.否<br>8.不知道	15	Has your youngest child had cough caused by illness at any time in the last 2 weeks (from *month/day* till *today*)?<br>Please respond: child had cough caused by illness/child did not have cough caused by illness [Zhao County's Maternal and Child Health Hospital message]	您最小孩子在过去 2 周内 *（...月... 日到...今天）*有过因为生病引起咳嗽吗？<br>请回复：有过生病引起的咳嗽/没有过生病引起的咳嗽【县妇幼短信】
	CO.2.a.	Did (*name*) breathe faster than usual with short, fast breaths or have difficulty breathing *(local terms)* in the last 2 weeks (please think back from two weeks ago till today)?<br>1.Yes<br>2.No→*skip to* CO.3<br>8.Do not know→ *skip to* CO.3	您最小孩子过去 2 星期内*（从今天算起往前推 2 星期，并强调日期）*，呼吸是否比平时快而短，或有呼吸困难（喘不上气/憋得慌）吗？<br>1.是<br>2.否——>*转到 CO.3 核查* 8.不知道——>*转到 CO.3 核查*	16	Did your youngest child breathe faster than usual with short, fast breaths or have difficulty breathing in the last 2 weeks (from *month/day* till *today*)?<br>Please respond: child had/child didn't have [Zhao County's Maternal and Child Health Hospital message]	您最小孩子 2 周内 *（...月... 日到...今天）*有过呼吸比平时快而短，或者有喘不上气/憋得慌吗？<br>请回复：有过/没有【县妇幼短信】
	CO.2.b	Were the symptoms due to a problem in the chest or a blocked nose?<br>1.Problem in the chest→ CO.3<br>2.Blocked nose→ CO.3<br>3.Both→ *skip to* CO.3<br>4.Other, specify: ____→ CO.3<br>8.Do not know→*skip to* CO.3	这些症状是因为肺部有问题还是因鼻塞引起的？<br>1.肺部问题——>*转到 CO.3 核查*<br>2.鼻腔堵塞——>*转到 CO.3 核查*<br>3.两者都有——>*转到 CO.3 核查*<br>4.其他原因______——>*转到 CO.3 核查*<br>8.不知道——>*转到 CO.3 核查*	17	What's the reason for the fast breathing or difficult breathing?<br>Please respond: problem in the chest/blocked nose/both problem in the chest and blocked nose/other reason……(please give reason) [Zhao County's Maternal and Child Health Hospital message]	您的孩子呼吸快或喘不上气/憋得慌是因为什么？<br>请回复： 肺部问题/鼻腔堵塞/两者都有/其他原因（请列出）【县妇幼短信】

	CO.3	*Check answers in CO.1 for fever and CO.2 for cough.*<br>*"No" for fever and "No" for cough→skip to next*<br>*"Yes" for and/or "Yes" for cough →ask:CO.4*	调查员核查：<br>CO.1 或<br>CO.2<br>1.只要有一项选"1.是"——> *继续问* CO.4 治疗指导；<br>2.都没有选"1.是"——>*结束 CO 部分，转到下一部分*	18	During this last episode of fever or cough in your youngest child, did you seek advice or treatment outside the home (ask anyone who is not a family member living with you)?<br>Please respond: yes, outside the home/no, at home. [Zhao County's Maternal and Child Health Hospital message]	您最小孩子最近那次发烧/咳嗽时，除了住在一起的家人，您还寻求指导或治疗了吗？<br>请回复：寻求过/没有寻求【县妇幼短信】
	CO.4	During this last episode of fever or cough in (*name*), did you seek advice or treatment for the fever/cough outside the home?<br>1.Yes→*skip to* CO. 4.b<br>2.No→*skip to* CO.4.a<br>8.Do not know	您最小孩子最近这次发烧/咳嗽时，除了住在一起的家人，您还寻求过指导或治疗吗？<br>1.是，到家庭外寻求指导或治疗——>*转到 CO.4.b*<br>（"到家庭以外寻求指导或治疗"包括询问邻居、去医院、为孩子举行宗教仪式等。医生到家中给予指导或治疗，也算在内。当母亲外出寻求指导或治疗时，是否带孩子一起去没有关系，如去药店买药没带孩子，也算在内。不要读出）<br>2.否，在家自行处理——>*转到 CO.4.a*<br>8.不知道——>*结束本部分，转到下一部分*			

	CO.4.a	During this last episode of fever or cough in (*name*), why didn't you seek advice? Only one answer allowed.<br>01.Mild disease/did not need outside help<br>02.Geographical access (too far from facility)<br>03.Costs (had to pay for visit or transportation)<br>04.Facility closed/staff not available<br>05.Poor quality of care at facility<br>06.Not necessary<br>07.Religious beliefs<br>08.Other: specify ________<br>88.Do not know	您最小孩子最近这次发烧/咳嗽时，没有寻求指导或治疗的主要原因？*[单选] ——> 答完此题，转到下一部分*<br>01.不严重/不需要别人帮助<br>02.家离卫生机构太远<br>03.费用（没钱支付服务或交通费）<br>04.卫生机构没有人/没有开门<br>05.卫生机构的服务质量差<br>06.没必要<br>07.宗教信仰<br>08.其他：_______<br>88.不知道	19	During this last episode of fever or cough in your youngest child, why did you not seek advice outside the home? Please respond by giving us one reason, the most important one. [Zhao County's Maternal and Child Health Hospital message]	您最小孩子最近那次发烧/咳嗽时除了住在一起的家人，您为什么没有寻求指导或治疗?<br>请回复一个最主要原因【县妇幼短信】

	CO.4.b	Where did you seek advice or treatment when (*name*) had fever or cough? *Record all sources mentioned. Prompt: "Anywhere else?"*<br>1.Relative or friend<br>11.Own family<br>1.Yes 2.No<br>12.Friends or neighbours<br>1.Yes 2.No<br>2.Health facility<br>21.County level hospital or above (excluding MCH hospital)<br>1.Yes 2.No<br>22.County level MCH hospital<br>1.Yes 2.No<br>23.Community health centre<br>1.Yes 2.No<br>24.Township hospital<br>1.Yes 2.No<br>25.Community health station<br>1.Yes 2.No<br>26.Village clinic<br>1.Yes 2.No<br>3.Private health facility<br>31.Private hospital<br>1.Yes 2.No<br>32.Private clinic<br>1.Yes. 2.No<br>33.Pharmacy<br>1.Yes 2.No<br>4.Community<br>41.Midwife<br>1.Yes 2.No<br>42.Staff for family planning<br>1.Yes 2.No<br>5.Other: 1.Yes 2.No Specify: _	您最小孩子最近那次发烧/咳嗽时，到哪里寻求指导或治疗？*[记录提到的所有地点。可提示"还有其他地方吗？"]*<br>1.家中亲友<br>11 自己的家人<br>1.是 2.否<br>12 朋友或邻居<br>1.是 2.否<br>2.公立医疗机构<br>21.县级及以上医院（不含妇幼保健院）<br>1.是 2.否<br>22.县级及以上妇幼保健院<br>1.是 2.否<br>23.社区卫生中心<br>1.是 2.否<br>24.乡镇卫生院<br>1.是 2.否<br>25 社区卫生站<br>1.是 2.否<br>26.村卫生室<br>1.是 2.否<br>3.私营医疗机构<br>31.私营医院<br>1.是 2.否<br>32.个体诊所<br>1.是 2.否<br>33.药店/药贩<br>1.是 2.否<br>4. 社区<br>41.接生员<br>1.是 2.否<br>42.计生干事<br>1.是 2.否<br>5.其他：1.是 2.否________	20	During this last episode of fever or cough in your youngest child, where did you seek advice or treatment when your youngest child had fever or cough?<br>Please respond by telling us all the places you went to. [Zhao County's Maternal and Child Health Hospital message]	您最小孩子最近那次发烧/咳嗽时您到哪里寻求指导或治疗？<br>请回复列出去的所有地方。【县妇幼短信】
				20a	*After response, prompt:*<br>Did you go anywhere else during the last episode of fever and cough in your youngest child? Please respond by telling us all the places you went to. [Zhao County's Maternal and Child Health Hospital message]	回复后，追问：<br>最小孩子最近那次发烧/咳嗽时，您还去过其他地方吗？请回复列出所有去的地方。【县妇幼短信】

End	NA	21	This is the end of the survey. Thank you very much for participating! You will receive 1 Jiǎo per text message and 5 Yuan for participating automatically on your mobile phone credit within two weeks. [Zhao County's Maternal and Child Health Hospital message]	调查结束，非常感谢您的参与！您将在 2 星期内得到返还的短信费及 5 元话费的补偿，将直接充到您的手机上。【县妇幼短信】

Local terminology study

Methods

We used a WHO guidance for identifying and validating local terms in Zhao County [3].

Participants and recruitment

We planned to interview fifteen caregivers and five village doctors. The study took place in Zhao Zhou Township and the findings could be used for the entire study, as local terms in one township are similar to local terms in other townships in Zhao County.

Data collection

We conducted interviews with doctors and caregivers on local terminology for diarrhoea and pneumonia signs and symptoms to ensure that the Chinese characters for those signs and symptoms in survey questions had a similar local meaning. One interviewer (YL), who was familiar with the local Zhao County dialect, conducted the interviews. A second researcher fluent in English (MV) was present during the interviews to make notes. We discussed all our findings with an integrated management of childhood illnesses expert (YZ) who had experience with local terminology studies, and with a Zhao County Maternal and Child Health Hospital doctor (XS).

The steps for identifying and validating the terms to communicate more effectively with caregivers were as follows:

(i) assess current information on local terms, if no consensus was reached in this step, conduct step 2, if consensus was reached proceed to step 3;

(ii) validate local terms through interviews with caregivers;

(iii) select local terms and adapt the questionnaire.

Step 1: Assess current information on local terms

We identified six terms used to describe diarrhoea, and cough and fever in the existing WHO MCNH questionnaire: diarrhoea, blood in stools, fever, cough, fast breathing, and difficult breathing. We aimed to assess the state of current information about those terms from three sources: written materials, interviews with doctors and interviews with caregivers. The interviewer recorded information about participants' demographics (type of caregiver, type of doctor, gender and age) and asked the doctors and caregivers about the words or phrases that caregivers in Zhaozhou Township used for each term. The interviewer emphasized that the aim of the interviews was to identify the terms that families used and encouraged participants to give more than one term when they believed that more than one term was applicable. The interviewer recorded the interviews with a digital tape recorder after asking for permission. We compared the information from these sources to assess the extent of consensus about the most appropriate local terms. If there was consensus for a particular term, there was no need to complete step 2.

Step 2: Validate local terms through interviews with caregivers

We aimed to validate the meaning and use of local terms for symptoms that did not reach consensus during step 1. As the purpose was to validate local terms, the interviewer took care to avoid giving the caregiver any signal to use "medical language". The interviewer used informal simple language, did not wear a coat and had a youthful appearance (caregivers were likely to expect doctors to be older). We planned the conduct the interviews in Zhao County Maternal and Child Health Hospital, because it was required that the data was collected in a setting where these symptoms were available, such as a hospital ward or outpatient clinic.

We aimed to find caregivers who had a child with physical signs, because the words caregivers used had to be compared to the physical signs. Those caregivers were asked questions to describe the condition of the child. However, as we were expecting that it could be difficult to find caregivers who had a child with physically present signs and were willing to participate, we also prepared videos from the WHO to show caregivers children with signs. In case there were no children available, we aimed to ask caregivers to tell us the words they used for the observable signs they saw in the videos. In addition, we asked caregivers to explain the meaning of the terms we found in step 1 to verify the meaning of those terms.

Step 3: Select local terms to adapt the questionnaire

We selected local terms that appeared to be most commonly used and understood based on our findings in step 1 and step 2. We recorded the terms of which we were confident that they were the best terms to use. These were the terms for which there was relative consensus. We discussed the chosen terms with the integrated management of childhood illnesses expert. We selected the terms for which there was relatively high consensus among caregivers who were interviewed. There was space for two of the most appropriate terms for each of the six terms in our survey. Therefore, we considered choosing two terms in case more than one term was appropriate. If there was a disagreement between the terms obtained in step 1 and step 2, we used the terms identified through the validation interviews in step 2. We recorded the terms that we finally selected and adapted the survey questionnaire.

Results

Step 1: assess current information on local terms

Written materials

Firstly, we attempted to find written materials on the local terms that we identified in the existing WHO MNCH questionnaire. We asked the Zhao County Maternal and Child Health Hospital doctor to provide us with written materials. We visited the department of pediatrics and the department of women and child health in Zhaozhou township hospital. We asked staff to provide us with written information, but they were not able to give us with written materials. Therefore, we used the terms that we identified in the existing questionnaire.

Doctors

Secondly, we asked five local doctors working in Zhao County Maternal and Child Health Hospital, Zhaozhou township hospital, and village clinics in Zhaozhou Township to participate in our interviews. All agreed to participate. The township hospital and Maternal and Child Health Hospital doctor were familiar with our study and helped us with contacting village doctors. The village doctors helped us with identifying caregivers. All doctors were male, apart from the township hospital doctor. Despite their busy schedule, the doctors were cooperative and able to find time for an interview. We conducted the interviews on a location of their preference, usually a

quiet room in their clinic. We asked them if the interview could take place at a time at which they were not likely to be interrupted. However, during one interview, a grandmother with her grandson walked in to ask advice for her child, and later a mother with her husband and child walked in. All the other interviews were not interrupted by patients.

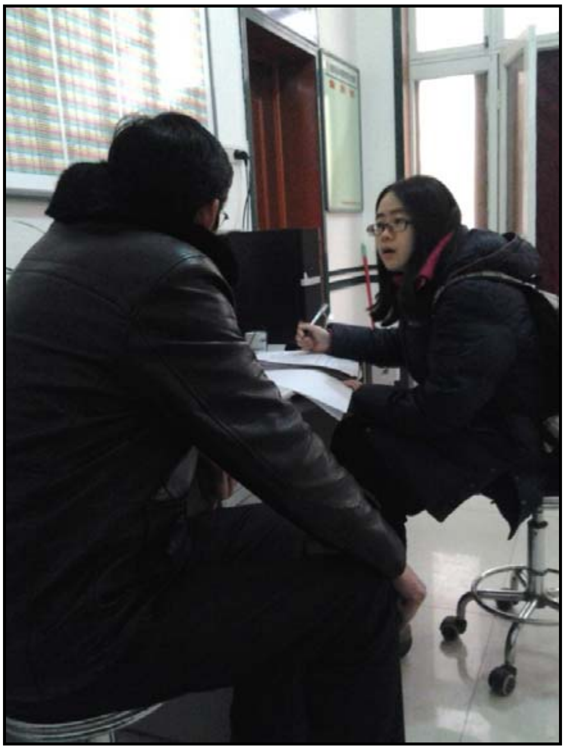

Village doctor and interviewer during local terminology interview

Photograph: the courtesy of Michelle Helena van Velthoven, personal collection

1. *Diarrhoea*

All doctors identified "拉肚子 Lādùzi", one of the two terms used in the existing WHO MNCH questionnaire. The second most used term was "拉稀 Lāxī". However, none of the doctors identified the other term in the existing questionnaire, "腹泻 Fùxiè".

2. *Blood in stools*

We found that “大便带血 Dàbiàn dài xiě” (which was the same as in the existing questionnaire) and “大便里边带血 Dàbiàn lǐbian dài xiě” (which was similar) were the most identified terms.

3. *Fever*

All doctors mentioned “发烧 Fāshāo” (which was the same as in the existing questionnaire), but no doctors mentioned the second term used in the existing questionnaire “发热 Fārè”.

4. *Cough*

For cough, all village doctors mentioned “咳嗽 Ké sou”, the same term that was used in the existing questionnaire.

5. *Fast breathing*

Doctors reached lower consensus on the terms for fast breathing. Three doctors mentioned “喘 Chuǎn”, which was also confusingly used by three doctors for difficult breathing (“喘 Chuǎn” means fast breathing). Two doctors mentioned “呼吸快 Hū xī kuài”, which had the same meaning as the term in the existing questionnaire (“呼吸比平时快而短 Hū xī bǐ píng shí kuài ér duǎn”).

6. *Difficult breathing*

Doctors also reached lower consensus on the terms for difficult breathing. Four doctors mentioned “憋得慌 Biē de huāng”. No one mentioned “呼吸困难 Hū xī kùn nán”, the term in the existing questionnaire.

Caregivers

Thirdly, we interviewed 12 caregivers in Zhao County Maternal and Child Health Hospital that serves a population that is typical for Zhaozhou Township.

1. *Diarrhoea*

Seven caregivers identified “拉肚子 Lādùzi”, one of the two terms used in the existing questionnaire. Also, seven caregivers used the term “拉稀 Lāxī”. However, similar to the doctors, none of the caregivers identified the other terms in the existing questionnaire, “腹泻 Fùxiè”.

2. *Blood in stools*

We found that “大便带血 Dàbiàn dài xiě” (which was the same as in the existing questionnaire) and “大便里边带血 Dàbiàn lǐbian dài xiě” (which was very similar) were the most commonly identified terms. Also, two caregivers mentioned the term “拉粑粑里边带血 Lā bābā lǐbian dài xiě” and one caregiver mentioned a very similar term “粑粑里边带血 Bābā lǐbian dài xiě”.

3. Fever

Nine caregivers mentioned "发烧 Fāshāo" (which was the same as in the existing questionnaire), but no caregivers mentioned the second term used in the existing questionnaire "发热 Fārè".

4. *Cough*

All caregivers identified "咳嗽 Ké sou", the same term that was used in the existing questionnaire.

5. Fast breathing

Similar to the doctors, caregivers also reached lower consensus on the terms for fast breathing. Eight caregivers mentioned a term similar to "呼吸快 Hū xī kuài" (which had a similar meaning as the term in the existing questionnaire "呼吸比平时快而短 Hū xī bǐ píngshí kuài ér duǎn"). We merged seven terms together into "呼吸快 Hū xī kuài" and combined the numbers of caregivers, because these terms were very similar and had the same meaning (Table S7; the results for the seven separate results are also shown). Five caregivers mentioned "喘 Chuǎn", or "喘气 Chuǎn qì", "喘 Chuǎn", and "喘得快 Chuǎn de kuài", which all meant fast breathing. However, these terms were confusingly also used for difficult breathing.

6. Difficult breathing

Caregivers also reached lower consensus on the terms for difficult breathing. Seven caregivers mentioned “喘不上气 Chuǎn bù shàng qì”, which was confusingly also used for difficult breathing. This term meant “cannot breathe out” and had a different meaning (by adding “bù shàng qì”) than “喘 Chuǎn” with the meaning “fast breathing”. No one mentioned the term in the existing questionnaire: “呼吸困难 Hū xī kùn nɑn”.

Conclusion

We selected the terms that were most used by caregivers and that were appropriate based on discussions within our team (Table S7). For two terms, fast and difficult breathing, there was low consensus (Table S8) and we aimed to validate these terms by doing step 2.

Step 2: validate local terms through interviews with caregivers

We interviewed four caregivers in Zhao County Maternal and Child Health Hospital. One caregiver wished to discontinue the interview, because she wanted to see her child and did not answer the questions about fast breathing. We aimed to find caregivers of children with fast or difficult breathing, but despite our efforts to find children, there were no children with these symptoms. Therefore, we used the videos to show caregivers fast and difficult breathing and asked them to tell us the words for these symptoms. However, as the videos were not specifically designed for caregivers but for health workers, caregivers were confused about the content of the videos and they could not explain which words they used for the symptoms that children in the videos had. Then we asked caregivers to explain us the meaning of the most appropriate terms for fast and difficult breathing that we identified in step 1. We found that caregivers could not distinguish fast and difficult breathing ("喘 Chuǎn"). Only one caregiver could explain the difference, but the three other caregivers could only explain what fast breathing was. However, to correctly answer the question related to these terms, caregivers did not have to distinguish between the two terms, because the question asked whether the child had fast or difficult breathing. All caregivers could explain the local terms for fast and difficult breathing that we found in first 1. Therefore, we chose these terms for the questionnaire (Table S8).

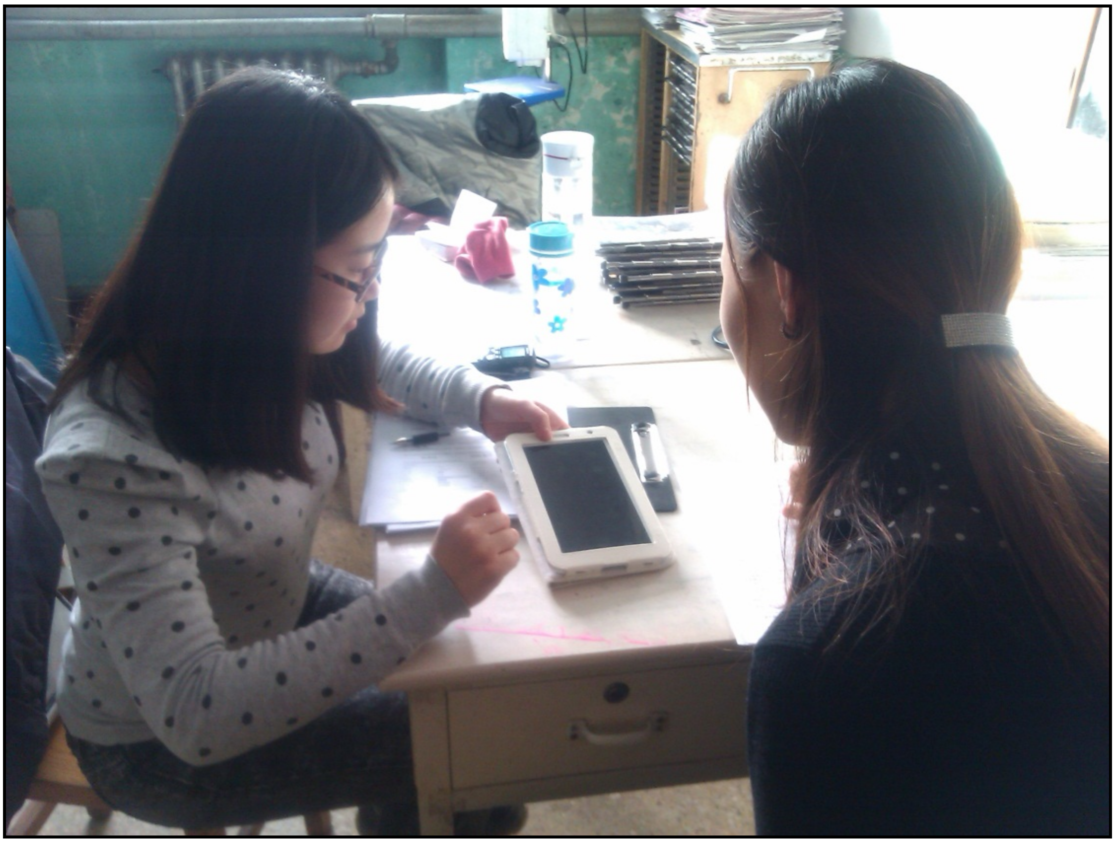

Mother and interviewer during validation interview

Photograph: the courtesy of Michelle Helena van Velthoven, personal collection

Step 3: select local terms to adapt the questionnaire

We selected the most appropriate terms by consulting the integrated management of childhood illnesses expert (Table S9). For four terms, diarrhoea, blood in stools, fever and cough, there was high consensus (Table S7). For diarrhoea we selected “拉肚子 Lādùzi” and “拉稀 Lāxī”. For blood in stools we selected “大便带血 Dàbiàn dài xiě” and “拉粑粑里边带血 Lā bābā lǐbian dài xiě”. For fever we selected “发烧 Fāshāo” and for cough “咳嗽 Ké sou”.

There was low consensus for the terms for fast and difficult breathing in step 1. Therefore, we attempted to validate these terms in step 2. However, this was very difficult, because there were no children with those symptoms and no appropriate videos available. Still we found terms that caregivers could understand. We decided to use the appropriate term “呼吸比平时快而短 Hūxī bǐ píngshí kuài ér duǎn” for fast breathing, which was very similar to “呼吸快 Hū xī kuài”, but more accurate and also understandable to caregivers. For difficult breathing, we decided to use both terms that caregivers identified in step 1: “喘不上气 Chuǎn bù shàng qì” and “憋得慌 Biē de huāng”. We decided not to use “喘 Chuǎn” with the meaning “fast breathing”, because we found in step 1 that caregivers also used this term for difficult breathing.

Table S7 Results of step 1: assess current information on local terms

Nr	Generic term in English	Term in existing WHO MNCH questionnaire in Chinese characters	Term in existing WHO MNCH questionnaire in pinyin	Local term (word or phrase used) in Chinese characters	Local term in pinyin	Meaning	N care-givers using term (12)	N doctors using term (5)	Chosen? *	Reason for choosing or not choosing the term
1	Diarrhoea	拉肚子<br>腹泻	Lādùzi<br>Fùxiè	拉肚子	Lādùzi	Loose stools, more times a day than usual	7	5	Yes	High consensus caregivers and doctors, used existing questionnaire
				拉稀	Lāxī	Loose stools, more times a day than usual	7	2	Yes	Appropriate, high consensus caregivers and doctors
				显拉把	Xiǎn lā bǎ	Loose stools, more times a day than usual	0	1	No	Only mentioned by 1 doctor
				拉粑粑	Lā bāba	Loose stools, more times a day than ususal, also used for normal stools	4	1	No	Confusing term with multiple meanings
				窜稀	Cuàn xī	Loose stools, more times a day than usual	3	1	No	Mentioned by minority of caregivers and doctors
				闹肚子	Nàodùzi	Loose stools, more times day than usual	1	0	No	Only mentioned by 1 caregiver
				跑肚	Pǎo dù	Loose stools, more times day than usual	1	0	No	Only mentioned by 1 caregiver

2	Blood in stools	大便带血	Dàbiàn dài xiě	大便带血	Dàbiàn dài xiě	Blood in stools	4	3	Yes	Mentioned by majority of caregivers and doctors, used in existing questionnaire
				大便里边带血	Dàbiàn lǐbian dài xiě	Blood inside stools	2	2	Yes	Very similar to “dàbiàn dài xiě”; “lǐbian” means inside
				大便里边有血	Dàbiàn lǐbian yǒu xiě	Blood inside stools (yǒu xiě=dài xiě = with blood)	1	1	No	Only mentioned by 1 caregiver and doctor
				大便出血	Dàbiàn chūxiě	Blood inside stools	1	0	No	Only mentioned by 1 caregiver
				大便边上有点血	Dàbiàn biān shàng yǒudiǎn xuè	Blood outside stools	0	1	No	Only mentioned by 1 doctor
				大便里边混有血	Dàbiàn lǐbian hùn yǒu xuè	Blood mixed in stools	0	1	No	Only mentioned by 1 doctor
				大便红	Dàbiàn hóng	Stools are red	0	1	No	Only mentioned by 1 doctor
				大便发红	Dàbiàn fā hóng	Stools look red	0	1	No	Only mentioned by 1 doctor
				拉粑粑里边血	Lā bābā lǐbian dài xiě	Blood inside stools, lā bābā was the local term for stools	2	0	Yes	Mentioned by 2 caregivers
				拉粑粑里边带点血丝	Lā bābā lǐbian dài diǎn xuèsī	Stripes of blood inside stools	1	0	No	Only mentioned by 1 caregiver
				粑粑里边带血	Bābā lǐbian dài xiě	Blood inside stools (bābā= lā bābā)	1	1	No	Only mentioned by 1 caregiver and doctor
				拉血	Lā xiě	Blood in stools	1	0	No	Only mentioned by 1 caregiver

3	Fever	发烧<br>发热	Fāshāo<br>Fārè	发烧	Fāshāo	Fever	9	5	Yes	High consensus among caregivers and doctors, used in existing questionnaire
				烧的慌	Shāo de huāng	Fever	0	1	No	Only mentioned by 1 doctor
				烧	Shāo	Fever	3	1	No	Similar to chosen term
				热呼呼	Rèhūhū	Very warm	1	0	No	Only mentioned by 1 caregiver
				烫	Tàng	Very hot	3	0	No	Mentioned by minority of caregivers and not mentioned by doctors
4	Cough	咳嗽	Ké sou	咳嗽	Ké sou	Cough	5	5	Yes	Very high consensus among caregivers and doctors, used in existing questionnaire

5	Fast breathing	呼吸比平时快而短	Hū xī bǐ píng shí kuài ér duǎn	呼吸快†	Hū xī kuài	The breath is fast	8	2	Yes	Appropriate merged term, mentioned by most caregivers and doctors, similar to term in existing questionnaire
				呼吸快	Hū xī kuài	The breath is fast	4	2		
				吸气快	Hū qì kuài	Breathing in fast	1	0		
				出气快	Chū qì kuài	Breathing out fast	2	0		
				气快	Qì kuài	The breath is fast	1	0		
				呼吸急促	Hū xī jí cù	The breath is short and fast	2	0		
				喘得快	Chuǎn de kuài	The breath is fast	2	0		
				喘气快	Chuǎn qì kuài	The breath is fast	1	0		
				喘†	Chuǎn	The breath is fast	5	3	No	Confusing as term was also used for difficult breathing
				喘	Chuǎn	The breath is short and fast	3	2		
				喘气	Chuǎn qì	The sound of the breath is loud/ breathing deeply	0	1		
				喘粗	Chuǎn cū	The sound of the breath is loud/ breathing deeply	1	0		
				呼歇喘气	Hū xīe chuǎn qì	Difficulty with breathing	1	0		
				吭吭	Kēng kēng	Cough with sound from the throat	0	1	No	Only mentioned by 1 doctor
				憋得慌	Biē de huāng	Feel uncomfortable because of lack of air	0	1	No	Only mentioned by 1 doctor
				喘不过来气	Chuǎn bù guò lái qì	Out of breath	1	0	No	Only mentioned by 1 caregiver
				呼噜	Hūlū	Make sounds from throat	0	1	No	Only mentioned by 1 doctor

				吸气费劲	Xī qì fèi jìn	Difficulty with breathing in	1	0	No	Only mentioned by 1 caregiver
				出气费劲	Chū qì fèi jìn	Difficulty with breathing out	1	0	No	Only mentioned by 1 caregiver
6	Difficult breathing	呼吸困难	Hū xī kùn nán	喘不上气†	Chuǎn bù shàng qì (Hébìng)	Cannot breath out	7	0	Yes	Appropriate merged term, mentioned most caregivers and doctors
				喘不上气	Chuǎn bù shàng qì	Out of breath	2	0		
				喘不过气	Chuǎn bù guò qì	Out of breath	1	0		
				喘气费劲	Chuǎn qì fèi jìn	Difficulty breathing	1	0		
				呼气费劲	Hū qì fèi jìn	Difficulty breathing out	1	0		
				出气费劲	Chū qì fèi jìn	Difficulty breathing out	1	0		
				喘不过气来	Chuǎn bù guò lái qì	Out of breath	1	0		
				喘不上来气	Chuǎn bù shàng lái qì	Out of breath	1	0		
				上不来气	Shàng bù lái qì	Out of breath	1	0		
				憋得慌†	Biē de huāng	Feeling uncomfortable because of lack of air	4	4	Yes	Appropriate merged term, mentioned by most caregivers and doctors
				憋得慌	Biē de huāng	Feeling uncomfortable because of lack of air	4	2		
				堵得慌	Dǔ de huāng	A feeling like something is blocked in the chest	1	1		
				憋气	Biē qì	Cannot breath	0	2		

	喘	Chuǎn	Breathing short and fast	4	2	No	Confusing as term was also used for fast breathing
	喘气	Chuǎn qì	The sound of breathing is loud/ breathing deeply	0	1	No	Only mentioned by 1 doctor
	喘得快	Chuǎn de kuài	Breath is fast	1	0	No	Only mentioned by 1 caregiver
	出气不匀实	Chū qì bù yún shí	The air breath out is not fluency	0	1	No	Only mentioned by 1 doctor
	气短	Qì duǎn	The interval/air of breath it short	2	0	No	Mentioned by minority of caregivers
	吭吭	Kēng kēng	Cough with sound from the throat	0	1	No	Only mentioned by 1 doctor
	发闷	Fā mèn	Feeling of not being able to breath	0	1	No	Only mentioned by 1 doctor

* Green is chosen local term, blue is not chosen local term.

†Merged term of different terms below the dotted line.

Table S8 Results of step 2: validate local terms through interviews with caregivers

Generic term in English	Term in existing questionnaire in Chinese characters	Term in existing questionnaire in pinyin	Local term (word or phrase used) in Chinese characters	Local term in pinyin	Meaning	Number of caregivers confirming meaning of this term (4)	Meaning according to caregivers
5 Fast breathing*	呼吸比平时快而短	Hū xī bǐ píngshí kuài ér duǎn	呼吸快/ 呼吸比平时快而短	Hū xī kuài/ Hū xī bū píngshí kuài ér duǎn	The breath is fast (merged)/ The breath is faster and shorter	3	"breath is fast (chuǎn qì kuài)"<br>"breath is fast"<br>"is "chuǎn" which means breath is fast and with sounds"
			喘	Chuǎn	The breath is fast (merged)	3	Caregivers could not distinguish "hū xī kuài" from "chuǎn"
6 Difficult breathing	呼吸困难	Hū xī kùn nán	喘不上气	Chuǎn bù shàng qì	Out of breath (merged)	4	"someone cannot breath (chuǎn bù shàng lái qì) or difficulty in breathing"<br>"difficulty in breathing because of lack of oxygen"<br>"someone cannot breath or breaths difficult"<br>"same meaning as biē de huāng"
			憋得慌	Biē de huāng	Feeling uncomfortable because of lack of air	4	"difficult to breath out"<br>"difficult to breath, lack of oxygen"<br>"difficult to breath (chuǎn qì kùnnán)"<br>"same meaning as chuǎn bù shàng qì"

*One caregiver did not complete the interview.

Table S9 Results of step 3: select local terms to adapt the questionnaire

Nr	Generic term in English	Local term in Chinese characters	Local term in pinyin
1	Diarrhoea	拉肚子<br>拉稀	Lādùzi<br>Lāxī
2	Blood in stools	大便带血<br>拉粑粑里边带血	Dàbiàn dài xiě<br>Lā bābā lǐbian dài xiě
3	Fever	发烧	Fā shāo
4	Cough	咳嗽	Ké sou
5	Fast breathing	呼吸比平时快而短	Hūxī bǐ píngshí kuài ér duǎn
6	Difficult breathing	喘不上气<br>憋得慌	Chuǎn bù shàng qì<br>Biē de huāng

Guide for cognitive interviews

Research questions

1. Do people understand the meaning of the information in text message format?

2. Do people understand how to respond to the text messages?

3. What do people prefer: receiving the information in one text message or in two separate text messages

Introduction

- Explain that we will try out sending text messages with questions about the health of the child of the participant to see if the questions and answers are clear to the participant
- Explain that we are testing the questions of the survey with people who take care of young children like the participant
- Explain that you will ask the survey questions and that the participant should answer them, just like a regular survey
- However, explain that your goal is here to get a better idea of how the questions are working. Explain that the participant should think aloud when they answer the survey questions, they should tell you everything that they are thinking about the questions when they are answering them
- Explain that sometimes you will ask more questions about the terms or phrases in the questions and what the participant thinks a question is asking about. Also, say that you will be taking notes.
- Explain that the participant should keep in mind that you really want to hear all opinions and reactions. He or she should not hesitate to speak up whenever something seems unclear, is hard to answer, or does not seem to apply to them.
- Tell the participant that you estimate that the interview will last about 30 minutes
- Ask the participant if he or she has any questions before you start.

Procedures

Example question 1: Diarrhoea is the passage of 3 or more loose or liquid stools per day. Has (NAME) had diarrhoea in the last 2 weeks (from… till ……)? Text us the number of your answer: 1.Yes, 2.No, 8.Do not know"

Sent the text message to the participant and ask the following:

1. Please explain to me in your own words what the health information, diarrhoea is the passage of 3 or more loose or liquid stools per day, means to you.
2. Please explain to me in your own words what the question means to you.
3. Please explain to me in your own words what the options mean to you.

Ask the participant to respond to the text message and ask the following:

4. Please think aloud when you reply to the message and tell me how you come to your answer.
5. What is your experience with replying to the message?
6. If you would write this (the information, question and answers) yourself, how would you write it in your own words?
7. Would you prefer this information to be in one text messages or in separate text messages? Why?

Repeat this procedure for all the survey questions

Now I would like to ask you a few questions about providing information on your child's health. I mean by this that you would answer questions like before on child illness signs and symptoms, such as diarrhoea, fever, cough, and fast breathing, by text messaging.

1. Which method would you like best: a face-to-face interview, phone call or text message? Why?
2. Would you be willing to answer questions on whether your child has been ill via text messaging? Why?

   (if this is not acceptable) What would we have to do different to make you willing to participate?
3. How many messages would you be willing to answer on one day?
4. How often would you be willing to provide information on whether your child has been ill?
5. Reminder
   a. Would it be ok to receive a reminder if you forget to answer a question?
   b. When would we have to send this?
   c. How many reminders at most?
   d. Which one do you like best, that the reminder is the original question or a text message in which just remind you to respond?

Questionnaire for interviews about reasons for different responses

Ask for all the questions for which the answer given face-to-face is different from the answer given via text message:

1. Can you think back in time, think about the moment you received the text message question about (*insert here what the question was about*). You answered this question by replying to us via text message. Can you tell me everything you remember about your experience and thoughts when you received the message and how you replied to it?
2. You just answered the same question to me face-to-face. Can you tell me everything about your thoughts about coming to your answer and replying to the question?
3. Why do you think the answer you gave for this question via text message is different from the response you gave face-to-face? (misunderstood text message question, misunderstood face-to-face question, changed mind, put wrong answer in text message, gave no response to text message question, other____)

Description of sending text messages

We chose the time interval for checking incoming messages based on experiences during previous research (unpublished). We found that the response rate was the highest between 9 am and 2 pm; then it dropped in the late afternoon and rose in the evening from 6 pm. Therefore, we checked the messages every 10 minutes between 9 am and 10 am, every 15 minutes between 10 am and 3 pm, and every 30 minutes between 3 pm and 6 pm. At 6 pm, we sent reminder messages to all the participants who did not respond to any of the questions and to participants who responded to one or more questions, but who did not complete the survey. Between 6 and 7 pm, we checked the incoming messages every 10 minutes. Between 7 and 9 pm, we checked the incoming messages every 15 minutes. We sent the final messages on the first day at 9 pm. We sent a second reminder on the next day at 9 am.

References

1. Coons SJ, Gwaltney CJ, Hays RD, Lundy JJ, Sloan JA, Revicki DA, et al. Recommendations on evidence needed to support measurement equivalence between electronic and paper-based patient-reported outcome (PRO) measures: ISPOR ePRO Good Research Practices Task Force report. Value Health. 2009;12(4):419-29. Medline:19900250 doi:10.1111/j.1524-4733.2008.00470.x
2. Willis G. Cognitive Interviewing, A "How To" Guide 1999. Available from: http://www.dfpp.univr.it/documenti/OccorrenzaIns/matdid/matdid823948.pdf. Accessed 9 August 2013.
3. WHO. E: Protocol for Identifying and Validating Local Terms. IMCI adaptation guid: A guide to identifying necessary adaptations of clinical policies and guidelines, and to adapting the charts and modules for the WHO/UNICEF course. Geneva: World Health Organization, Department of Child and Adolescent Health and Development; 2002.
